# *N*-acyl-homoserine lactone-based quorum sensing beyond canonical lineages: insights from Actinomycetota

**DOI:** 10.3389/fmicb.2026.1738013

**Published:** 2026-04-20

**Authors:** Christy R. Handel, Rebecca D. Prescott, Chien-Chi Lo, Timothy J. O’Donnell, Landon Balkwill, Joshua Gurr, Jimmy H. Saw, Gayatri Sharma, Jingjing Wang, Patrick D. Curtis, Alan W. Decho, Philip G. Williams, Patrick S. G. Chain, Stuart P. Donachie

**Affiliations:** 1 School of Life Sciences, University of Hawaiʻi at Mānoa, Honolulu, HI, United States; 2 Department of Environmental Health Sciences, Arnold School of Public Health, University of South Carolina, Columbia, SC, United States; 3 Department of Biology, University of Mississippi, University, MS, United States; 4 Biosciences Division, Los Alamos National Laboratory, Los Alamos, NM, United States; 5 Department of Chemistry, University of Hawaiʻi at Mānoa, Honolulu, HI, United States; 6 Department of Biological Sciences, The George Washington University, Washington, DC, United States; 7 Office of Community Science, ‘Iolani School, Honolulu, HI, United States

**Keywords:** Actinomycetota, *Bradyrhizobium*, *Brenneria*, *N*-acyl-homoserine lactones, *Pseudonocardia*, quorum sensing, *Rhodococcus*

## Abstract

Classically, quorum sensing (QS) is defined as a form of bacterial communication that regulates gene expression in response to population density and other environmental conditions. While a variety of QS systems exist, *N*-acyl homoserine lactone-based quorum sensing (AHL-QS) is the best characterized in Gram-negative Pseudomonadota (formerly Proteobacteria), often in medically relevant taxa. However, it remains unclear what AHL-QS systems look like in underexplored environments, and how often or far the canonical model extends beyond the Pseudomonadota. Here, we investigated AHL production in environmental Bacteria from underexplored environments, including two newly described Pseudomonadota species, *Brenneria uluponensis* K61^T^, from a taro lo‘i in Hawai‘i, and Bradyrhizobium prioriisuperbiae BL16A^T^, from a lava cave on the Island of Hawai‘i, and two Gram-positive Actinomycetota (formerly Actinobacteria), *Pseudonocardia alni* GV4, from a culture of Gloeobacter violaceus, and *Rhodococcus kroppenstedtii* Y88A, from a dehydrated hypersaline mat on San Salvador Island, Bahamas. Custom hidden Markov models (HMMs) were used to identify putative *luxI/R* homologs, followed by analyses of genomic context, luxI/R-family protein phylogeny, and LuxR domain architecture. AHL production was assessed using liquid chromatography-multiple reaction monitoring-mass spectrometry (LC-MRM-MS), and targeted gene disruption was performed in *R. kroppenstedtii* Y88A. Putative luxI/R homologs were identified in all strains. In *R. kroppenstedtii* Y88A, disruption of the sole luxI homolog resulted in loss of all detectable AHLs, indicating that this gene (designated rhdI) is required for AHL production under the tested conditions. Across Actinomycetota genomes, *luxI* genes occurred as solitary elements rather than canonical *luxI/R* pairs and were associated with genes linked to metabolism and redox processes. Phylogenetic analyses revealed that Actinomycetota *luxI/R*-family proteins diverge from canonical systems, with LuxR-family proteins lacking canonical autoinducer-binding domains and instead comprising only helix-turn-helix regulatory architectures. Together, these findings expand the known diversity of AHL-QS systems into underexplored microbial lineages and environments and suggest broader ecological roles for AHL signaling. The canonical *luxI/R* model derived largely from Pseudomonadota may represent only one of several evolutionary architectures for AHL signaling, raising the possibility that AHL production in Actinomycetota operates through regulatory frameworks distinct from classical AHL-QS systems.

## Introduction

Quorum sensing (QS) is a form of microbial intercellular chemical signaling that regulates gene expression at a community level (Fuqua et al., 1994; Miller and Bassler, 2001; Waters and Bassler, 2005). *Bacteria* are known to utilize one or more QS systems that employ different classes of signaling molecules to induce specific responses to environmental conditions (Cornforth et al., 2014). However, the most extensively studied class of signals are *N*-acyl homoserine lactones (AHLs). In classical or canonical *N*-acyl homoserine lactone-based QS (AHL-QS) systems, a *luxI* gene encoding an AHL synthase is paired with a cognate *luxR* gene which encodes an AHL-responsive transcriptional regulator. Together these form an autoinducing circuit in which cells respond to their own signals, described as “self-talk” (Papenfort and Bassler, 2016). Early models of AHL-QS emphasized a sufficient density of cells (i.e., a quorum) in proximity to each other as the primary determinant of signal accumulation to a threshold concentration. Subsequent work has expanded this framework, demonstrating that AHL-QS reflects the interplay between production, diffusion, degradation, spatial structure, regulatory control, and communication not only within individual cells or populations, but also between neighboring cells and distinct populations (Decho et al., 2010; Prescott and Decho, 2020; Spacapan et al., 2023).

It was previously accepted that AHLs were produced only by Gram-negative bacteria, especially the Pseudomonadota (Case et al., 2008; Papenfort and Bassler, 2016; Wellington and Greenberg, 2019), and this is commonly accepted today (Willey et al., 2026). However, recent studies have challenged this idea, with AHLs documented in cultures of Cyanobacteria (Sharif et al., 2008), methanogenic *Archaea* (Zhang et al., 2012), and several Gram-positive bacteria, including *Exiguobacterium* sp. (phylum Bacillota; Biswa and Doble, 2013), *Salinispora arenicola* and *S. pacifica* (phylum Actinomycetota; Bose et al., 2017), and six well-known *Streptomyces* species (phylum Actinomycetota; Salehi-Najafabadi et al., 2024). The detection of AHLs in Gram-positive bacteria challenged the longstanding view that these organisms interact with AHLs only antagonistically (Kaufmann et al., 2005). A bioinformatic analysis also suggested a putative *luxI* homolog in the Gram-positive bacteria *Enterococcus gallinarum* (Subramoni et al., 2015), and recent studies suggest certain Nitrospirota (nitrite-oxidizing bacteria) harbor *luxI* genes, although experimental evidence has not yet been reported. How widespread AHL-QS is outside the Pseudomonadota is unknown, but bioinformatic surveys suggest it may occur in many bacterial lineages (Rajput and Kumar, 2017; Barriuso and Martínez, 2018).

The detection of AHL-QS systems in a range of organisms raises questions about how these systems operate in underexplored ecological settings, such as nutrient-limited, spatially heterogeneous, or chemically extreme environments (Wong et al., 2019; Charlesworth et al., 2019; Urvoy et al., 2022; Peng et al., 2025). In natural environments and some pathogenic contexts, microorganisms exist within diverse interactive communities where signaling is likely not solely antagonistic, but may also support cooperative interactions between Gram-positive and Gram-negative bacteria (Garcia-Betancur and Lopez, 2019; Prescott and Decho, 2020; Moreno-Gámez et al., 2023). These systems are further shaped by environmental conditions that influence signal synthesis, stability, and diffusion (Decho et al., 2011). However, it remains unclear how well the canonical AHL-QS model, derived largely from well-studied Pseudomonadota (previously Proteobacteria), applies beyond this lineage in such environments. Our understanding of AHL-QS is still grounded in classical models, despite the likelihood that intercellular signaling dynamics and regulatory architectures in nature may differ substantially.

To date, no studies have experimentally validated the genetic basis of AHL production through gene disruption, heterologous expression, or related approaches in any Gram-positive bacteria, or in Gram-negative bacteria outside the Pseudomonadota, except for the methanogenic archaeon *Methanothrix harundinacea* 6Ac (Zhang et al., 2012), to the best of the authors’ knowledge. Only 38 LuxI-family proteins are currently listed in UniProt with experimentally confirmed gene function, and nearly all belong to the Pseudomonadota. This limits phylogenetic studies of *luxI/R* genes outside this phylum. Identifying more AHL-QS systems in phylogenetically diverse groups will lead to a better understanding of the evolutionary history of this communication system.

In canonical AHL-QS systems, LuxR-family transcriptional regulators are characterized by the presence of both an N-terminal autoinducer binding domain (ABD) and a C-terminal helix-turn-helix (HTH) DNA-binding domain. Upon entry into the cell, the AHL ligand binds to the ABD of LuxR-family proteins, inducing conformational stabilization or rearrangement. The resulting AHL-LuxR complex binds to a promoter region (*lux* box sites) in the DNA to modulate gene expression (Whitehead et al., 2001; Waters and Bassler, 2005). This architecture can give the impression of strict one-signal, one-receptor specificity. However, LuxR-family proteins can be ligand-flexible, sometimes binding multiple AHLs, including endogenous AHLs or AHLs made by other species of microorganisms, allowing for eavesdropping on others’ signals (Luo et al., 2024; Schwieters and Ahmer, 2025). Evolutionarily, this ligand “promiscuity” is thought to provide plasticity and adaptive value by expanding the sensory space of mixed species communities, supporting interspecies and interkingdom communication (Wellington and Greenberg, 2019).

Surveys of LuxR-family proteins reveal substantial variability in domain architecture, including regulators that retain the conserved HTH domain but lack a detectable ABD. This suggests a broader functional and evolutionary diversity of the LuxR protein family that requires further investigation (Covaceuszach et al., 2013; Subramoni et al., 2015; Hudaiberdiev et al., 2015; Bez et al., 2021). Although the ABD is characteristic of canonical AHL-responsive LuxR proteins, its absence does not preclude classification within the LuxR family when sequences cluster phylogenetically with established LuxR homologs and retain the conserved DNA-binding domain. In this study, proteins lacking recognizable ABD signatures are therefore also referred to as LuxR-family regulators based on their HTH conservation and phylogenetic placement, while acknowledging that their ligand-binding properties may differ from canonical AHL-responsive LuxRs.

Bioinformatic analyses suggest that canonical *luxI/R* architectures represent only one of several genomic possibilities. Some genomes encode multiple *luxI/R* pairs, as well as genes that are unpaired within the local genomic neighborhood, defined here as approximately 3,000 base pairs (bp; following Subramoni and Venturi, 2009; Venturi and Ahmer, 2015). The most commonly described unpaired loci are LuxR-family “solos,” which lack a nearby LuxI homolog. Substitutions in their ABDs are also common, suggesting diversification beyond canonical AHL responsiveness (Patel et al., 2013; Bez et al., 2023; Schwieters and Ahmer, 2025; Fu et al., 2025). Although less common, LuxI-family solos have also been identified and experimentally validated in Pseudomonadota, including *Ruegeria* sp. KLH11 and *Sphingobium* spp., demonstrating that AHL synthesis can occur in the absence of a nearby LuxR-family regulator (Zan et al., 2015; Gan et al., 2015).

Additionally, it is often reported that a specific bacterial strain produces one type of AHL. However, some bacteria synthesize multiple AHLs even when only one *luxI-*family gene is identified (Doberva et al., 2017). In most cases, AHL production is carried out by LuxI-type AHL synthases, though other types of AHL synthases exist (e.g., LuxM/HdtS; Waters and Bassler, 2005; Dong et al., 2017). It is therefore likely that some AHL synthases can direct the synthesis of multiple signal molecules with a single enzyme (Milton et al., 2001; Ortori et al., 2007), or exhibit broad substrate specificity, utilizing acyl-ACP donors of varying chain lengths and oxidation states (Gould et al., 2006; Prescott and Decho, 2020).

Here, we examined AHL production, AHL-QS gene topology and architectures, and the phylogenetic relationships of AHL-QS systems in four bacterial strains isolated from underexplored environments, comparing them with experimentally verified AHL-QS systems in Pseudomonadota and with genomes of Gram-positive bacteria recently reported to harbor AHL-QS systems. These strains include two newly described Gram-negative Pseudomonadota, *Brenneria uluponensis* K61^T^ and *Bradyrhizobium prioriisuperbiae* BL16A^T^, and two Gram-positive Actinomycetota, *Pseudonocardia alni* GV4 and *Rhodococcus kroppenstedtii* Y88A. Together, these organisms provide a framework for examining AHL-based signaling across phylogenetically and ecologically distinct bacteria from environments that remain poorly represented in QS studies.

## Materials and methods

Four bacterial strains (referred to as K61^T^, BL16A^T^, GV4, and Y88A) were isolated from different sites and maintained as pure cultures in the Donachie lab at the University of Hawaiʻi at Mānoa (Table 1; Prescott et al., 2023). Culture purity was assessed by observing live cells in sterile distilled water by light microscopy under a 100x oil immersion objective, Gram stains of 24-h cultures, and whole genome sequencing metrics such as GC% of contigs and genome binning tools. Working stocks of cultures were re-plated monthly, and archived stocks were stored at −80 °C in liquid media containing 30% (*v/v*) glycerol. Strain-specific methods are given below, with a brief introduction to each strain.

**Table 1:** Isolates in this study.

Isolate	Abbreviation	Origin	Accession, BioSample, and BioProject	References
*Brenneria uluponensis* K61^T^	K61^T^	Loʻi at Ulupō Heiau on the eastern edge of Kawainui Marsh on Oʻahu	NZ_CP135917 SAMN37483218 PRJNA1019399	Prescott et al. (2023)
*Bradyrhizobium prioriisuperbiae* BL16A^T^	BL16A^T^	Biofilm from the wall of Kaūmana cave on the windward side of the island of Hawaiʻi in 2017	NZ_CP135921 SAMN37483212 PRJNA1019399	Prescott et al. (2023)
*Pseudonocardia alni* GV4	GV4	Isolated from a consortium of at least four strains that grow with the ancient Oxyphotobacteria *Gloeobacter violaceus*	NZ_JARWLD000000000 SAMN34135175 PRJNA954151	Butdee et al. (2025)
*Rhodococcus kroppenstedtii* Y88A	Y88A	Rehydrated microbial mat that had been desiccated for 10 years from a hypersaline pond on San Salvador Island, The Bahamas	NZ_CP135915 SAMN37483221 PRJNA1019399	Prescott et al. (2023)

Summary of the four bacterial isolates analyzed, including their abbreviations, environmental origins, and associated genomic metadata (GenBank accession numbers, BioSample, and BioProject identifiers), along with corresponding references. Isolates were obtained from diverse and underexplored environments.

### Gram-negative Pseudomonadota*: Brenneria uluponensis* K61^T^ and *Bradyrhizobium prioriisuperbiae* BL16A^T^

K61^T^ (Accession: NZ_CP135917; BioProject: PRJNA1019399; BioSample: SAMN37483218) was isolated from a loʻi at Ulupō Heiau on the eastern edge of Kawainui Marsh, Oʻahu as part of a study of plant-growth promoting *Bacteria* in basaltic soils (Prescott et al., 2023). Mud from the loʻi was diluted with sterile distilled water, and sub-samples of the diluted mud were spread on glucose-yeast extract (GYE) agar (per liter: 10 g dextrose, 10 g Yeast Extract, 14.2 g Difco Bacteriological Agar). Strain K61^T^ arose after 72 h of incubation and was purified through repeated transfers on GYE.

To study AHLs produced by K61^T^, six bottles containing 500 mL of GYE broth were prepared. Three bottles were amended with a MgSO₄-enriched brine to a final concentration of 10% *v/v* to examine AHL-QS stability and behavior under osmotic stress and ion toxicity in sulfate-dominated conditions similar to some volcanic conditions. The brine composition was used in prior geochemical studies of basalt-rich evaporitic environments (brine IIa; Tosca et al., 2011; Stevens et al., 2019), and consisted of 50.9% MgSO₄·7H₂O, 0.41% KHCO₃, 0.15% KCl, 1.14% MgCl₂·6H₂O, and 0.9% NaCl, and is referred to here as MgSO₄ brine. Each bottle was inoculated with 1 mL of mid-log growth phase culture of K61^T^. At 24, 48, and 72 h, each culture’s optical density (OD) was measured in a spectrophotometer at 525 nm. AHLs produced during 72 h in different conditions, e.g., GYE, and GYE with MgSO_4_ brine, were extracted and compared by environment and time.

BL16A^T^ (Accession: NZ_CP135921; BioProject: PRJNA1019399; BioSample: SAMN37483212) was isolated from an epilithic biofilm collected in Kaūmana cave, part of a lava tube on the windward side of the island of Hawaiʻi in 2017 (Prescott et al., 2023). BL16A^T^ grew on nutritionally rich potato dextrose agar (PDA) and nutritionally poor Reasoner’s 2A agar (R-2A; Reasoner and Geldreich, 1985), was incubated at room temperature, and purified through repeated transfers on PDA and R-2A. Two bottles, one containing 1 L of potato dextrose broth (PDB), and the other containing 1 L of R-2A broth, were inoculated with 1 mL overnight culture of BL16A^T^. Five days post-inoculation, each culture’s OD was measured as described above, and AHLs were extracted.

### Gram-positive Actinomycetota: *Pseudonocardia alni* GV4 and *Rhodococcus kroppenstedtii* Y88A

GV4 (Accession: NZ_JARWLD000000000; BioProject: PRJNA954151; BioSample: SAMN34135175) was isolated from a consortium with the cyanobacterium *Gloeobacter violaceus* (PCC 7421) by streaking on R-2A agar and incubating at room temperature (20–22 °C). One liter of R-2A broth was inoculated with 1 mL of an overnight culture of GV4. Five days post-inoculation, the culture’s OD was determined as described above, and AHLs were extracted.

Y88A (Accession: NZ_CP135915; BioProject: PRJNA1019399; BioSample: SAMN37483221) was isolated from a microbial mat collected from a hypersaline pond on San Salvador Island, Bahamas. The mat was first desiccated in 2010 and stored for ~10 years in a closed container at ~10% relative humidity. A 2 × 4 cm section of the desiccated mat was rehydrated in ~30 mL sterile seawater (~34% salinity) in a Petri dish, and incubated at room temperature for 1 week. Y88A was isolated and purified from a spread plate of a 50 μL sub-sample of the saline water and mat material on GYE agar and incubated at room temperature for 2 weeks. Subsequently, 1 mL of an overnight culture of Y88A in GYE broth was inoculated in 1 L of GYE broth and incubated for 5 days at 30 °C, at which point the culture’s OD was measured as described above, and AHLs were extracted.

### Whole genome sequencing of all strains

DNA was extracted from each strain using commercial extraction kits (Supplementary Table 1). Kit protocols were followed for K61^T^, BL16A^T^, GV4, and Y88A. Strains were homogenized by mechanical cell disruption in a BeadBug™6 Microtube Homogenizer for 60 s at 4350 RPM (Benchmark Scientific, Sayreville, NJ, United States). Whole-genome libraries were prepared using the Nextera XT DNA Library Prep Kit (Illumina, San Diego, CA, United States). Library fragment size and distribution were determined on a Bioanalyzer High Sensitivity DNA chip (Agilent Technologies, Santa Clara, CA, United States), quantified in the Quant-iT PicoGreen dsDNA Assay Kit (Invitrogen, Carlsbad, CA, United States), normalized, and pooled. Each library was sequenced on an Illumina MiSeq in the Advanced Studies in Genomics, Proteomics and Bioinformatics (ASGPB) at the University of Hawai‘i at Mānoa. The fragments sequenced were 301 base-pairs (bp) long. A minimum of 50X genome coverage was the target for the short-read sequencing, given that long-read sequencing was also planned for each strain (described below).

DNA was also sequenced using a MinION (Oxford Nanopore Technologies, Oxford, United Kingdom). Libraries were prepared with the Nanopore Rapid Barcoding kit (SQK-RBK004), loaded onto a MinION R9.4 Flow Cell, and sequenced in a MinION Mk1B sequencer. Flow cells were run for 24–72 h, depending on the strain from which the DNA was derived (Supplementary Table 1) using the MinKNOW software. MinION sequencing for strains BL16A^T^, K61^T^, and Y88A was conducted by students at St. Andrews Priory School (Honolulu, Hawaiʻi), ‘Iolani School, and Moanalua High School, respectively, as part of the ʻĀina Informatics Network program. MinION sequencing of GV4 was conducted in the Donachie laboratory. Long-read MinION data was subsampled to improve the assembly, using anywhere from 10 to 50% of the data generated (Supplementary Table 1).

A hybrid assembly of Illumina and Nanopore sequences was completed using Unicycler with the EDGE (Empowering the Development of Genomics Expertise) Bioinformatics platform (Li et al., 2017; Wick et al., 2017). Assembled genomes were annotated in EDGE using Prokka (Seemann, 2014) with an in-house HMM (described below), and searched for putative *luxI/R* genes. The National Center for Biotechnology Information (NCBI) accession numbers for each strain can be found in Table 1.

The nucleotide sequence of the 16S rRNA gene in each strain was compared with those of type strains in GenBank and on the EzTaxon server (Altschul et al., 1997; Yoon et al., 2017). A genome-based taxonomy of each strain was determined at the Type (Strain) Genome Server (TYGS, Leibniz Institute, DSMZ) web server at https://tygs.dsmz.de/: the 16S rRNA gene nucleotide sequence extracted from the full genome was aligned with other 16S rRNA sequences in the genomes of related type strains, and pairwise sequence similarities were calculated through the Genome-to-Genome Distance Calculator (GGDC, Leibniz Institute, DSMZ) web server at http://ggdc.dsmz.de/ (Meier-Kolthoff et al., 2013a, 2013b).

### AHL-QS gene homolog searches: hidden Markov models and topology

Hidden Markov Models are dynamic Bayesian networks that can capture hidden information from observable, known sequential symbols (e.g., a nucleotide sequence), if they share an evolutionary history. They can predict the function of a nucleotide or protein sequence based on known genes with the same function and a similar sequence. We developed HMMs for AHL-QS genes and protein families of *luxI* and *luxR* (Prescott et al., 2023). LuxI-family autoinducer synthase proteins were obtained from UniProt in May 2019 with entry IPR001690 (Supplementary Table 2 lists the LuxI proteins used). The corresponding gene sequences of those proteins were downloaded and used to develop a LuxI HMM. LuxR-family proteins were obtained from UniProt in May 2019 based on the presence of LuxR-family domain annotations, including InterPro entries IPR000792, IPR005143, and IPR036693, and were used to develop a separate LuxR HMM. IPR000792 corresponds to the conserved C-terminal LuxR-family HTH DNA-binding domain, whereas IPR005143 and IPR036693 annotate the N-terminal LuxR-family ABD, with IPR005143 integrating the Pfam entry PF03472. Inclusion of these domain annotations allowed the LuxR HMM to capture gene sequences encoding canonical LuxR-family proteins containing both ABD and HTH domains, as well as LuxR-family proteins containing only the ABD or only the HTH domain, which are considered evolutionarily related members of the LuxR protein family. For both LuxI and LuxR HMMs, the entries were filtered with the term “taxonomy:Bacteria, length:[100 TO 500]”, as most known LuxI/R-family proteins range from ~200–300 amino acids (aa). Gene lists were further curated manually based on the gene product entry, and if that was related to AHL-QS or other QS systems based on this description, with removal of LuxR entries described as a “two-component” system, which can include non-AHL-based QS systems. LuxR lists were combined, and duplicate records were removed (Supplementary Table 3 lists LuxR proteins used). HMMs were then constructed using MAFFT v7.429 (Katoh and Standley, 2013) for multiple sequence alignment and HMMER 3.1b1 to build the model^1^.

To determine false positive rates, we generated negative control datasets for both LuxI and LuxR HMMs. For LuxI, we compiled a LuxI-negative dataset by searching UniProt entries for acyl-transfer-related enzymes and non-AHL-QS synthases, retaining entries only for *Bacteria* and Swiss-UniProt reviewed entries, removing any AHL synthases/transcriptional regulators, and preserving sequences spanning a broad phylogenetic diversity by selecting at least one entry from all genera. This created a list of 53,303 non-LuxI genes. For LuxR, UniProt was searched for “transcriptional regulator AND NOT LuxR”, yielding 47,753 sequences. Approximately 10% of this set was selected across all bacterial genera to maximize phylogenetic breadth. We then ran the LuxI and LuxR HMMs against these negative sets to estimate false positive rates. We determined that the false positive rate was 0.011% for the LuxI model and 0.12% for the LuxR model, and were able to estimate an e-value cutoff of 1.0 × 10^−10^ for both LuxI/R models based on average false positive *e*-values.

Using the EDGE Bioinformatics platform, these models were run against the genomes of the strains in this study, along with the following Gram-positive genomes used in phylogenetic analyses: *Salinispora arenicola* DSM 45545 (GCF_000384275.1), *Salinispora pacifica* DSM 45546 (GCA_000384095.1), *Streptomyces lavendulae* subsp. *lavendulae* strain CCM-3239 (GCA_002803845.1), *Streptomyces griseus* DSM 40236^T^ (GCA_900105705.1), *Streptomyces nodosus* DSM 40109^T^ (GCA_014205035.1), *Streptomyces coelicolor* A3(2) (GCA_000203835.1), *Streptomyces clavuligerus* DSM 738^T^ (GCA_028752555.1), and *Streptomyces lividans* TK24 (GCA_000739105.1). In EDGE, we used Prokka for annotation with hmmscan (command - hmms) and Prokka’s built-in profiles. JBrowse (in EDGE Bioinformatics) and Geneious Prime 2025.1.2 reviewed genome topology and identified genes in the *luxI/R* neighborhoods (±10 kb) as possible cognate pairs of *luxI/R* genes or any other potential transcriptional regulators, and the function of nearby genes. Sequences of *luxI/R* genes were converted into amino acid sequences. LuxI/R homologs were further evaluated using protein length (with LuxI proteins typically ~157–257 aa, and canonical LuxR proteins of ~210–272 aa), directionality, conserved residues commonly found in canonical LuxI/R systems, and presence or absence of C-terminal HTH domain and N-terminal ABD of LuxR-family proteins using InterProScan (Jones et al., 2014).

### Phylogenetic analyses

Amino acid sequence alignments of LuxI- and LuxR-family proteins were generated using reference sequences from organisms with experimentally validated *luxI/R* genes and sequences identified in this study using MAFFT 7.526 with the L-INS-I algorithm (Katoh and Standley, 2013), and trimmed with TrimAl using the following parameters: -gt 0.5 (Capella-Gutiérrez et al., 2009). Models for amino acid substitutions selected were based on the best-fit model chosen according to the Bayesian Information Criterion (BIC) obtained by the IQ-TREE model testing tool version 2.4.0 (Minh et al., 2020). IQ-TREE (version 2.4.0) was used to construct maximum likelihood phylogenies of LuxI- and LuxR-family proteins using the following parameters: -m LG + F + I + G4 (LuxI) or Q.pfam+F + G4 (LuxR) -alrt 1,000 -bb 1,000. Resulting consensus trees were then visualized on the iTOL web tool (Letunic and Bork, 2021).

### AHL extractions

The AHLs were extracted from culture supernatants of all four strains using acidified ethyl acetate, following established protocols optimized for neutral lipids (Shaw et al., 1997; Ortori et al., 2007). Distilled ethyl acetate containing 0.1% formic acid was added to 500 mL cultures of K61^T^ and 1 L cultures of BL16A^T^, GV4, Y88A, and mutant strains Y88A-3 and Y88A-11 (see below for targeted gene disruption methods). The volume of ethyl acetate added in this step was equal to the culture volume. Cultures and ethyl acetate were mixed for 24 h on a magnetic stirrer at medium speed, or until the solution was homogeneous, and then allowed to settle under gravity. Aqueous and cell debris layers (bottom layers) were drained using a separatory funnel, and the organic layer was evenly distributed into 50 mL polypropylene centrifuge tubes (Corning Falcon™) and centrifuged at 10,000 x *g* for 15 min. The organic, upper phase from each tube was then pooled into a 1,000 mL round-bottom flask and evaporated in a rotary evaporator. The residue was re-suspended in acidified ethyl acetate, pooled in 2 dram vials, and further dried under a slow stream of cool, filtered (0.2 μm) air.

### AHL standard preparation for LC-MRM-MS

Utilizing standard LC-MRM-MS techniques on a triple quadrupole mass spectrometer, 10 AHL standards were analyzed by multiple reaction monitoring (MRM) to assess the presence and relative concentrations of AHLs. Standards were prepared before each LC-MRM-MS run (Supplementary Table 4). We did not aim to quantify AHL levels, but rather aimed to determine if AHLs were present, and if so, what was the nature of the diversity of AHL molecules in the different strains under different growth conditions. As such, the data are not assumed to serve as a basis for comparative quantification, and are not suited for drawing conclusions about AHL concentration beyond relative comparisons.

As this was a targeted analysis, only the AHLs listed in Supplementary Table 4 could be identified, although many more are known (D’Angelo-Picard et al., 2005; Williams et al., 2007; Decho et al., 2009; Crépin et al., 2012; Doberva et al., 2017). Stock solutions of each AHL standard were prepared at 1 mg/mL by weighing each in an 8 mL borosilicate glass vial, followed by the addition of LC–MS-grade acetonitrile, except for C14-AHL, whose poor solubility in acetonitrile required the use of 1-butanol. A mixed stock solution of all AHLs was prepared by combining 10 μL of each 1 mg/mL stock solution in an LC–MS vial, followed by dilution to 1 mL with LC–MS-grade acetonitrile. The final concentration of each AHL was 10 μg/mL, and this solution was serially diluted as required for the standard series.

### LC-MRM-MS

Dried sample residues of all four bacterial strains were re-suspended in 1 mL LC*–*MS-grade acetonitrile and vigorously vortexed and sonicated. Suspensions were filtered through a 0.45 μm PVDF filter disk to remove insoluble impurities and transferred directly into LC*–*MS vials for analyses. Acetonitrile diluted samples when required. Chromatographic separations were performed with an Agilent 1,200 series binary HPLC pump using a reversed-phase gradient of LC*–*MS-grade H_2_O and acetonitrile, both modified with 0.1% formic acid. 10 μL of each sample was injected onto an Agilent Zorbax Eclipse XBD-C18, 80 Å, 1.8 μm HPLC column (AG927975-902) equilibrated with 10% aqueous acetonitrile. AHLs were eluted using a linear gradient, at a flow rate of 0.8 mL/min, from 10 to 100% acetonitrile over 5 min, followed by 3 min at 100% acetonitrile. Acyl homoserine lactones were detected on an Agilent 6,410 triple quadrupole mass spectrometer via electrospray ionization (+ESI). The following mass spectrometer parameters were used with positive polarity: Gas Temp, 350 °C; Drying gas, 12 L/min; Nebulizer, 55 psi; VCap, 4,000 V; Fragmentor 100 V; Collision energy 5; Cycle time 500 ms. Data were processed using Quantitative (B3.01) and Qualitative Analysis (B3.02) software from Agilent, using calibration curves generated at the start and end of each data collection. Concentrations of the eight external standards ranged from 6.25 to 800 ng/mL. The tandem MS analysis was programmed to look for the AHL precursor and product ions within an appropriate chromatographic window. The transition at *m/z* (mass-to-charge ratio) 102, a characteristic fragment ion corresponding to the homoserine lactone ring, was used to quantify collision-induced dissociation for each AHL species.

We included negative controls during the cultivation process for our isolates (i.e., inoculating and monitoring blank media), but we did not extract and analyze media-only samples during processing because the instrumentation and methods employed (triple quadrupole LC-MRM-MS) lack sufficient sensitivity to detect AHLs at the extremely low levels expected in growth media alone, if they were to be found at all. In some instances, data from inoculated broth samples allowed the relative concentration of AHLs to be determined, while in others, the response was below the limit of quantification on this instrument. For these reasons, and because unknown AHLs for which we did not have standards were detected, the absolute peak area of the AHLs in the chromatograms was used to determine presence or absence, and to facilitate relative comparisons with other samples.

Other lipids produced by each strain that did not match the 10 AHL standards, but exhibited the diagnostic neutral loss of a homoserine lactone ring (*m/z* = 102), were annotated as putative unknown AHLs. This approach specifically targeted fragments with an *m/z* = 102 as part of a neutral loss experiment. The parent ion of lipids that underwent fragmentation to produce the *m/z* = 102 ion was identified and annotated. In some cases, when standards are present, the retention time could be correlated and used as a third parameter to help with the identification of the putative unknown AHL.

### Insertional disruption of *luxI* in Y88A

*Rhodococcus kroppenstedtii* Y88A was grown in GYE broth for all cloning and transformation steps. Cultures were incubated at 30 °C for up to 36 h, until growth was observed, and genomic DNA was extracted. Briefly, 1.5 mL of culture was centrifuged (17,000 x *g*, 2 min), and the pellet was resuspended in 80 μL of 10 mM Tris buffer. Cell lysis was aided by mechanical homogenization with metal beads at 1000 rpm for 2–3 min in a homogenizer. To improve DNA yield, 25 μL of lysozyme (25 mg/mL) was added, and the suspension was incubated at room temperature for 15 min. The mixture was then incubated in a water bath at 55 °C for 10 min. Following this, 100 μL of AL lysis buffer (Qiagen DNeasy Blood and Tissue Kit) was added, and after vortexing, the mixture was incubated at 37 °C for 10 min. Proteinase K (10 μL) was added, and the mixture was incubated at 56 °C for 30 min. DNA was purified on a standard silica column as described in the Qiagen DNeasy Blood and Tissue Kit protocol.

To disrupt the *luxI* gene, a 420 bp genome fragment was PCR amplified using primers p4247-*luxI*F (5′-tcgcgagacgtccaattgcaGCGCTGCTCGCTCTGCCGAG-3′) and p4247-*luxI*R (5′-accggtacgcgtaacgttcgGTACCGCAGGATGCCGGACC-3′). The PCR product was purified using the Zymo Clean and Concentrator kit and assembled into the linearized p4247 integrating plasmid using Gibson Assembly (Thanbichler et al., 2007). The p4247 vector was linearized with NdeI and EcoRI restriction enzymes before assembly.

The Gibson Assembly reaction was chemically transformed into *Escherichia coli* DH5α competent cells. Transformed cells were incubated at 37 °C for 1 h, then plated on Luria-Bertani agar containing kanamycin (20 μg/mL). Plates were incubated overnight at 37 °C. Colonies were screened by colony PCR using the primers p4247pCRConf-F (5′-GGCGACAAGGTGCTGATGCC-3′) and p4247pCRConf-R (5′-GCTGCAAGGCGATTAAGTTGGG-3′). The expected PCR amplicon size was 756 bp, comprising 420 bp from the *luxI* gene insert and 336 bp from the vector backbone. Positive colonies were selected and grown for plasmid isolation.

The recombinant p4247 plasmid containing the *luxI* fragment was electroporated into Y88A using a Gene Pulser apparatus (Bio-Rad) set at 2988 V, 25 μF capacitance, and 750 *Ω* resistance in a 2 mm gap cuvette. After electroporation, cells were recovered in GYE broth for 6 h at 30 °C and plated on GYE supplemented with kanamycin (20 μg/mL). Plates were incubated at 30 °C. Transformants appeared after approximately 3 days.

Disruption of *luxI* was confirmed using colony PCR. Phusion high-fidelity polymerase was used with the following conditions: initial denaturation at 98 °C for 30 s; 30 cycles of 98 °C for 10 s, 58 °C for 30 s, and 72 °C for 2 h 30 min, followed by a final extension at 72 °C for 10 min. Primers used for confirmation were Conf1ccp4247-*luxI*-F (5′-GTCAACAAGGACGAGGAAGC-3′) and Conf1ccp4247-*luxI*-R (5′-GATTAGCGACCACCAGGGTG-3′). The expected product size was 4,651 bp, which was observed in two colonies. PCR products of the expected size from those colonies were purified and sequenced using the primers Seq*luxI*F (5′-GTGACGGGGTCCGGCCCGTT-3′) and Seq*luxI*R (5′-accggtacgcgtaacgttcgGTACCGCAGGATGCCGGACC-3′). Sequencing results confirmed successful disruption of the *luxI* gene. Two mutants were obtained and are referred to as Y88A-3 and Y88A-11. No genetic or chemical complementation was performed because *R. kroppenstedtii* Y88A is a non-model environmental isolate for which established complementation systems are not currently available.

To confirm if Y88A-3 and Y88A-11 strains were still able to produce AHLs or not, cultures of both strains were grown in GYE broth, following the same procedures for AHL extractions and mass spectrometry described above. Scanning electron microscopy (SEM; Hitachi S-4800 Field Emission Scanning Electron Microscope) in the Biological Electron Microscope Facility (BEMF) at the University of Hawaiʻi at Mānoa assessed the morphology of Y88A^WT^, Y88A-3, and Y88A-11.

## Results

### Genomic organization of *luxI/R* loci in Gram-positive Actinomycetota

**Figure 1:**
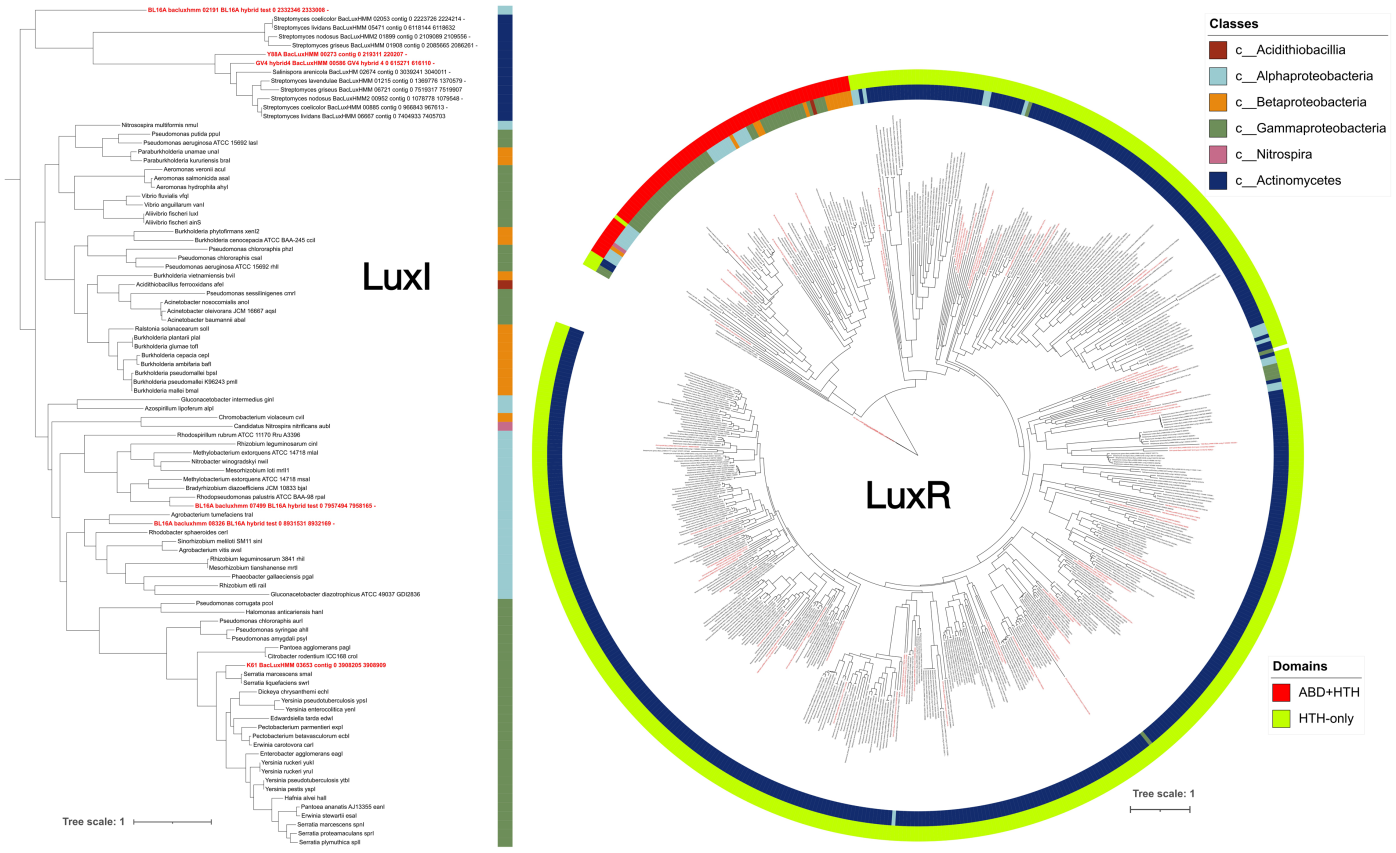
Maximum-likelihood phylogenetic trees of LuxI and LuxR homologs identified in this study and from organisms with experimentally verified *luxI/R* QS systems. Amino-acid sequences were aligned using MAFFT (L-INS-i) and trimmed with TrimAl (gap threshold 0.5). The best-fit substitution models (-m LG+F+I+G4 for LuxI or Q.pfam+F+G4 for LuxR; -alrt 1000-bb 1000) were selected by BIC in IQ-TREE and used to construct trees with 1000 SH-aLRT and ultrafast bootstrap replicates. Trees were visualized in iTOL. Sequences from *Brenneria uluponensis* K61^T^, *Bradyrhizobium prioriisuperbiae* BL16A^T^, *Pseudonocardia alni* GV4, and *Rhodococcus kroppenstedtii* Y88A are highlighted in red. Classes represented include Acidithiobacillia, Alphaproteobacteria, Betaproteobacteria, Gammaproteobacteria, Nitrospira, and Actinomycetes, along with LuxI/R homologs from Gram-positive *Salinispora* and *Streptomyces* spp. known to participate in QS. LuxR protein architecture (HTH-only vs. ABD+HTH) is represented by the outer ring. Luxl/R homologs from the Gram-negative Proteobacteria generally clustered with canonical AHL systems, whereas those from Actinobacteria grouped within distinct clades. Notably, one BL16A LuxI homolog (BL16A_bacluxhmm_02191) clustered with Actinobacteria, and several α- and γ-proteobacterial LuxR sequences were interspersed among Gram-positive clades.

In-house HMM-based annotation identified a single *luxI* homolog in each of the Gram-positive Actinomycetota genomes GV4 and Y88A (GV4_hybrid4_BacLuxHMM and Y88A_BacLuxHMM_00273, respectively) along with multiple *luxR* genes. HMM-based analyses detected 45 *luxR* homologs in GV4 and 15 in Y88A. Similar patterns were observed among Gram-positive Actinomycetota previously identified as AHL producers that were included in a broader bioinformatic survey in this study. HMM-based gene annotation identified a single *luxI* gene in *Salinispora arenicola* DSM 45545 and 23 *luxR* genes, whereas *S. pacifica* DSM 45546 contained no detectable *luxI* genes, but has 28 *luxR* genes. *Streptomyces lavendulae* subsp. *lavendulae* strain CCM-3239, *S. griseus* DSM 40236^T^, *S. nodosus* DSM 40109^T^, *S. coelicolor* A3(2), *S clavuligerus* DSM 738^T^, and *S. lividans* TK24 (Salehi-Najafabadi et al., 2024) were also evaluated and, except for *S. clavuligerus* DSM 738^T^, all contained a single *luxI* gene and between 42 and 79 *luxR* genes (Figure 1). All LuxR-family proteins identified in these Actinomycetota lack recognizable ABDs but retain conserved HTH DNA-binding domains, designated here as HTH-only LuxR-family proteins (Figure 1 and Supplementary Figure 4 is also provided, which can be magnified for easy reading).

**Figure 2:**
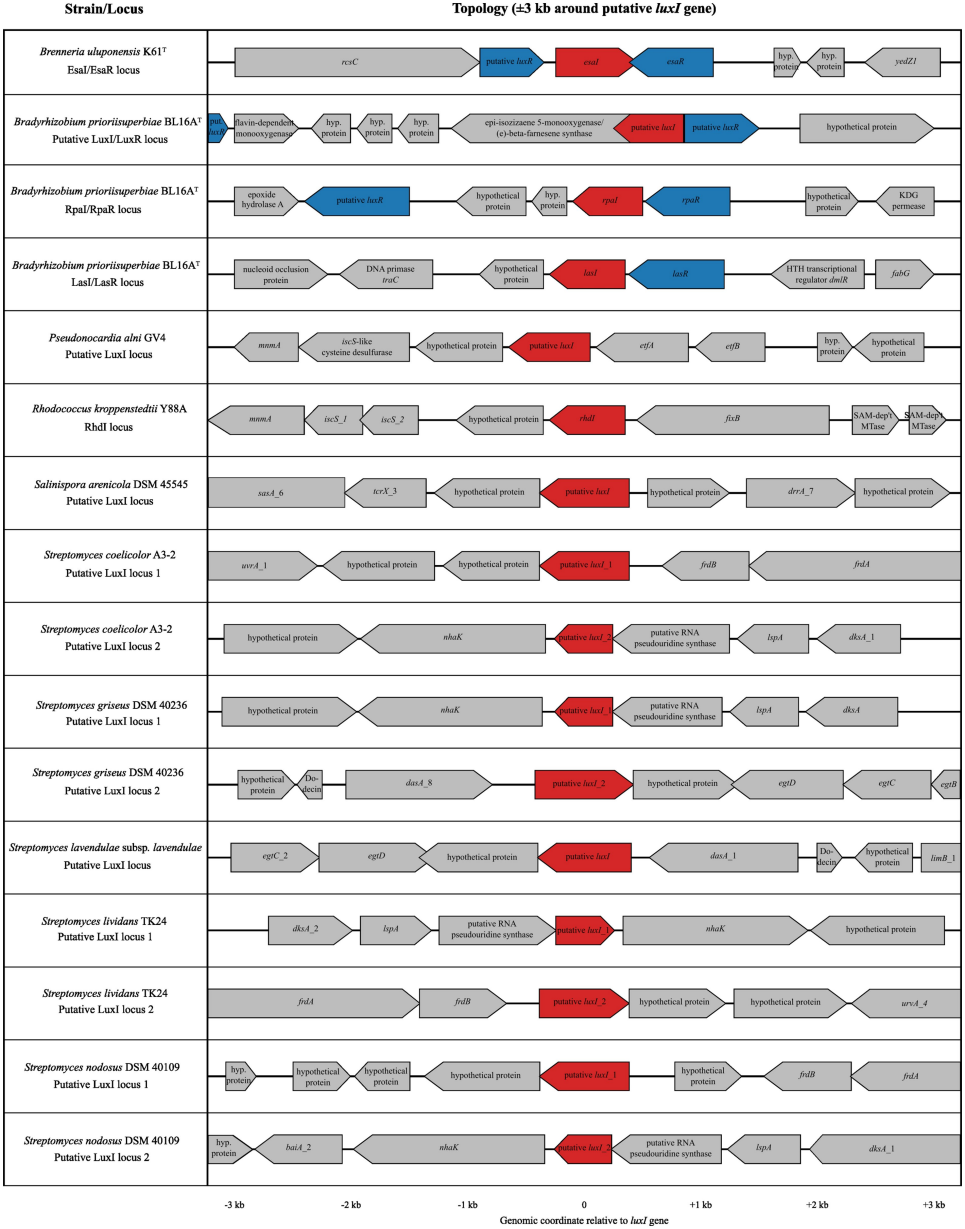
Genomic neighborhood organization of putative luxI loci across diverse bacterial taxa. Gene neighborhood topology is shown for representative strains *Brenneria uluponensis* K61^T^, *Bradyrhizobium prioriisuperbiae* BL16A^T^, *Pseudonocardia alni* GV4, and *Rhodococcus kroppenstedtii* Y88A, as well as additional Gram-positive *Salinispora* and *Streptomyces* strains, with genes displayed within ±3 kb of each putative *luxl* gene. Putative *luxI* genes are highlighted in red, and *luxR* homologs, when present, are highlighted in blue. Flanking genes are annotated based on predicted function where available; hypothetical proteins are indicated as “hyp.” Gene orientation reflects transcriptional direction, and genomic positions are plotted relative to the luxI gene (0 kb). Both canonical *luxl/R* arrangements and non-canonical architectures, including luxI loci lacking adjacent luxR homologs and multiple luxI loci within a single genome, are shown. This comparison illustrates the diversity of luxI-associated genomic contexts across Gram-negative and Gram-positive bacteria. It is important to note that although genomic neighborhood analyses revealed *luxl/R* genes positioned adjacent to genes that could plausibly be QS-regulated and arranged in canonical QS orientations, functional validation is required to confirm the regulatory roles. Additionally, these strains may have more AHL-QS related genes, but they remain undetected due to database limitations.

To assess the genomic context of *luxI* homologs in Gram-positive bacteria, genomic neighborhoods were examined at two scales, focusing on ±3 kb for schematic visualization (Figure 2), and up to ±10 kb to capture broader neighborhood structure. The broader range was chosen to capture operon-scale organization and associated metabolic modules that may not be evident in narrower ±3 kb analyses. Across all examined Gram-positive genomes in this study, except for *S. pacifica* DSM 45546 and *S. clavuligerus* DSM 738^T^, in which no *luxI* genes were annotated, *luxI* homologs consistently occurred as solitary genes rather than canonical *luxI/R* pairs. No LuxR-family proteins containing ABD or HTH domains were identified in these extended neighborhoods (±10 kb). LuxI homologs were embedded in genomic regions enriched for metabolic and redox-associated genes.

The genomic architecture within ±3 kb of the *luxI* locus in both GV4 and Y88A is analogous; the homolog is flanked by genes involved in sulfur metabolism and tRNA modification, including *iscS* (cysteine desulfurase) and *mnmA* (tRNA-specific 2-thiouridylase; Figure 2). The *luxI* gene in GV4 shows it sits at the 5′ end of a co-linear, negative-sense block consisting of the *luxI* gene, followed by genes encoding electron transfer and redox-associated proteins, including electron transfer flavoprotein (ETF) subunits (*etfA*, *etfB*, and *fixB*) and cofactors (*S*-adenosylmethionine-dependent methyltransferase). The intergenic distance between the *luxI* and *etfA* genes is 124 bp, and *etfA* and *etfB* overlap by 46 bp (Figure 2). Three *luxR* genes are upstream of the *luxI* gene, but each is separated by intergenic distances exceeding 120 kb. Similarly, the region surrounding the *luxI* gene in Y88A revealed no adjacent *luxR* genes within ±3 kb (Figure 2), with the nearest *luxR* gene ~313 kb away. Near the *luxI* gene are genes encoding two *iscS*-like cysteine desulfurases, several hypothetical proteins, and *fixB*, a gene encoding an electron transfer flavoprotein homologous to *etfB* (Figure 2). These genes play central roles in tRNA modification and redox homeostasis (Kambampati and Lauhon, 1999; Arts et al., 2016). Their proximity to the *luxI* gene suggests a conserved association between AHL synthesis and cellular processes linked to sulfur metabolism, translation efficiency, and metabolic state.

In five of the six *Streptomyces* genomes studied bioinformatically, *luxI* homologs were found in regions enriched in genes encoding enzymes characteristic of secondary metabolism, including monooxygenases, dehydrogenases, transporters, and numerous hypothetical proteins (Figure 2). These neighborhoods lacked conserved regulatory proteins, but displayed enzymatic clustering typical of biosynthetic and degradative pathways. HTH-type transcriptional regulators were occasionally identified within ±10 kb of *luxI* homologs and were annotated as metabolic regulators (GltC- or LysR-type proteins). They lacked detectable ABDs and were not arranged in any particular configuration with the *luxI*.

In contrast, the genetic neighborhood surrounding the *luxI* homolog in the *Salinispora* genome did not exhibit the same enzyme-rich clustering typical of secondary metabolism, nor was it associated with tRNA modification or redox homeostasis. Instead, the *luxI* gene is positioned among genes associated with environmental sensing and stress response, including *sasA*, *tcrX*, and *drrA*, suggesting a distinct functional landscape (Figure 2). The neighborhood includes *sasA*, a sensor histidine kinase best characterized in Cyanobacteria as part of their circadian clock (Chang and LiWang, 2025), *tcrX*, a transcriptional regulator implicated in stress responses, including to iron limitation (Stupar et al., 2024), and *drrA*, an ATP-binding cassette (ABC) transporter most extensively characterized in *Deinococcus* species for its role in tolerance to oxidative and radiation-induced damage (Wang et al., 2008). This locus also lacks a LuxR-family protein, so this genomic neighborhood is more suggestive of environmental sensing, stress response, and cellular protection.

### LuxI homolog in *R. kroppenstedtii* Y88A, a Gram-positive Actinomycetota

**Figure 3:**
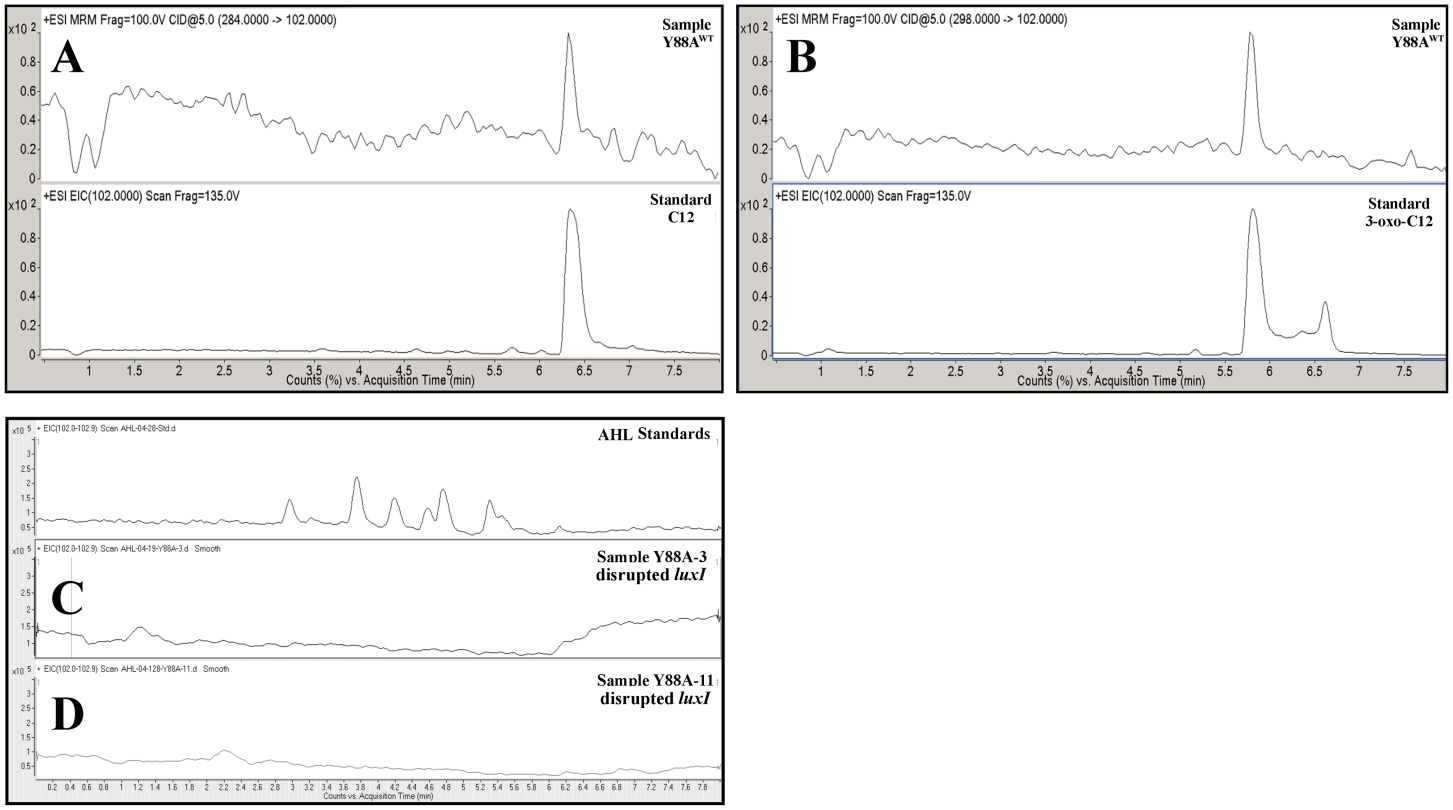
Chromatograms representing **(A)** C12- and **(B)** 3-oxo-C12-homoserine lactone (HSL) production by *Rhodococcus kroppenstedtii* Y88A^WT^ and the lack of AHL production by *luxI* disrupted Y88A-3 **(C)**, and *luxI* disrupted Y88A-11 **(D)** in Glucose-Yeast Extract (GYE) broth. The panels **(A,B)** indicate the detection of C12-oxo-HSL and of C12-HSL. Displayed are traces in which the parent ion fragmented to an *m/z*=102, indicative of the homoserine lactone ring. No peaks in panels **(C,D)** indicate there was no detection of AHLs. Please refer to Supplementary Figure 1 for the representative extracted ion chromatogram (EIC) of AHL standards at 12.5 ng/ml.

**Figure 4:**
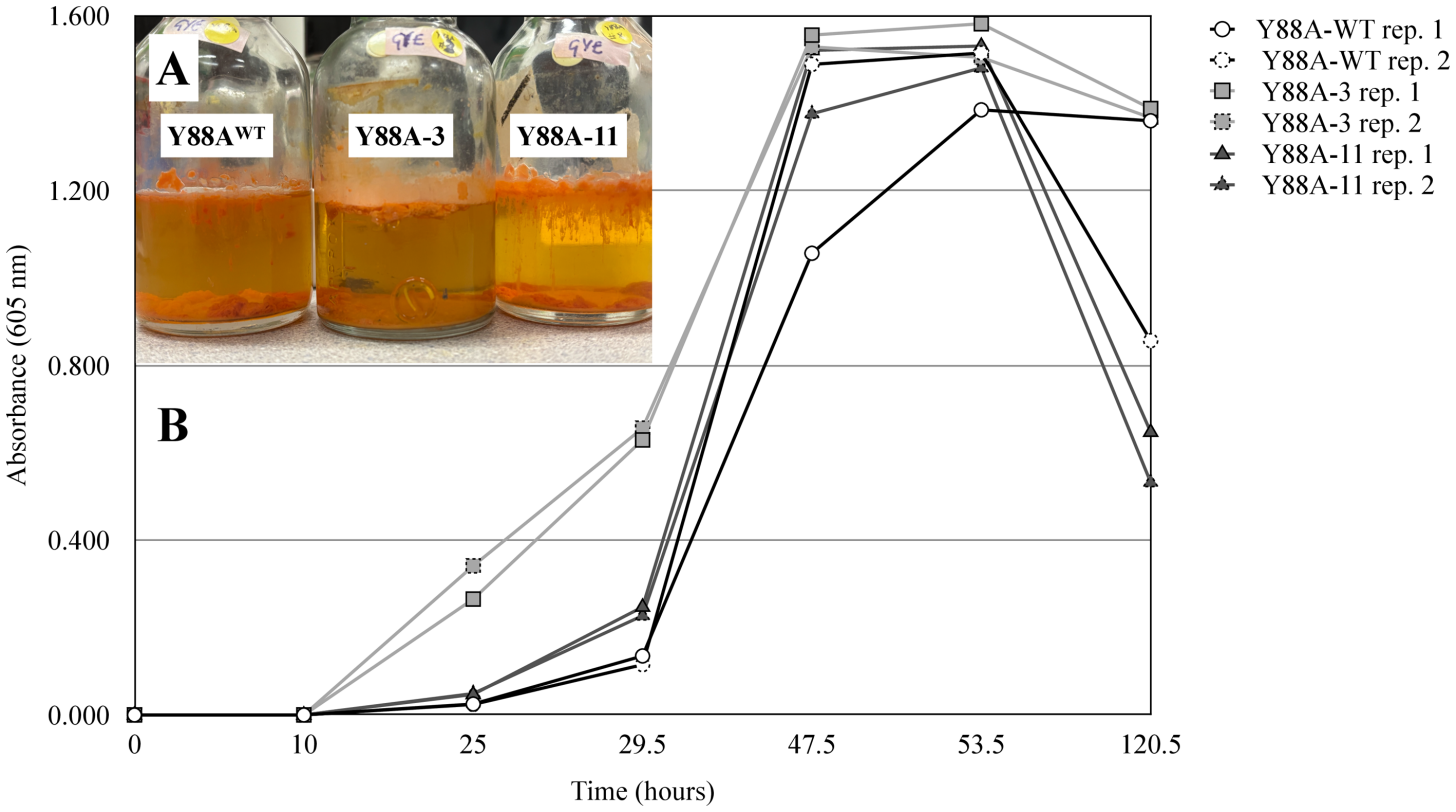
Growth curves of *Rhodococcus kroppenstedtii* Y88A^WT^, *luxI* disrupted Y88A-3, and *luxI* disrupted Y88A-11. **(A)** Growth of mutant strains Y88A-3 and -11 were similar to the wild type Y88A-WT, with thick biofilms produced in the media of all cultures. Any morphological changes observed in the mutants are within the range of normal growth variation, as environmental bacteria often display a broad repertoire of phenotypic states. **(B)** Absorbance measurements at 605 nm indicated that all three strains were at comparable growth stages, suggesting similar cell densities and indicating that the gene disruptions did not affect overall growth.

A targeted gene disruption experiment of the *luxI* homolog (Y88A_BacLuxHMM_00273; positions 219,311–220,207, negative-sense strand) in Y88A was performed, followed by AHL extraction and LC-MRM-MS analysis. Following targeted gene disruption, two independent *luxI*-disrupted strains (Y88A-3 and Y88A-11) were analyzed, with disruption confirmed by colony PCR and sequencing. No AHLs were detected in either strain grown under the same conditions as the wild type (GYE broth, 5 days; OD_605_ = 1.82 and 1.57, respectively; Figure 3), confirming that this gene is required for AHL production under the tested conditions. The growth trajectories of mutant strains were similar to those of the wild type (Figure 4), with thick biofilms produced in the media by all cultures. SEM of Y88A^WT^, Y88A-3, and Y88A-11 showed no apparent morphological differences (Supplementary Figure 3), and no other alterations in growth were observed. Overall, these results support the identification of a *luxI-*family gene responsible for AHL production in *R. kroppenstedtii* Y88A, here designated as *rhdI*.

### Genomic organization of *luxI/R* loci in Gram-negative Pseudomonadota

Unlike the Actinomycetota studied here, we identified canonical AHL-QS systems in both strains of environmental Pseudomonadota in this study. In-house HMM-based annotation identified one *luxI* gene and 12 *luxR* genes in the K61^T^ genome, of which four are canonical LuxR-family proteins and eight are HTH-only LuxR-family proteins (Figure 1). Of these, one cognate *luxI/R* gene pair was identified (K61_BacLuxHMM_03653 and K61_BacLuxHMM_03654, respectively; Figure 2), illustrating a classical AHL-QS system found in many well-studied Pseudomonadota. Initial Prokka annotations identified these genes as homologous to LuxI- and LuxR-family proteins EsaI and EsaR, respectively, originally described in *Pantoea stewartii* subsp. *stewartii* (McClean et al., 2012). The *luxI* gene is located adjacent to the *luxR* gene, reflecting a typical coupled *luxI/R* topology, and appears in a convergent/head-to-head arrangement. Upstream genes encode a sensor histidine kinase RcsC and an additional HTH-only LuxR-family protein. Downstream are two hypothetical proteins, followed by a methionine-sulfoxide reductase subunit YedZ1 (Figure 2).

We identified three putative *luxI* genes and 18 putative *luxR* genes in the BL16A^T^ genome. Of these, three are canonical LuxR-family proteins and 15 are HTH-only LuxR-family proteins (Figure 1). The three canonical LuxR-family proteins are paired with the three LuxI-type synthases as classical cognate *luxI/R* pairs (Figure 2), and each loci represent distinct evolutionary lineages (Figure 1).

The first *luxI/R* locus identified in BL16A^T^ (BL16A_bacluxhmm_02191 and BL16A_bacluxhmm_02192, respectively) occurs on different DNA strands with a 77 bp intergenic region. The surrounding genomic context of this locus differs substantially from those of the other two loci identified in BL16A^T^. Directly downstream of this *luxI* gene is a gene encoding a terpene-associated monooxygenase [(E)-β-farnesene synthase/epi-isozizaene monooxygenase], multiple genes encoding hypothetical proteins, and a gene encoding a flavin-dependent monooxygenase (2,5-diketocamphane 1,2-monooxygenase). This locus lacks the conserved metabolic or regulatory features observed adjacent to the two other *luxI/R* loci in this species. Instead, this locus is embedded in a region enriched in genes that encode enzymes associated with secondary metabolism and redox activity, underscoring the functional diversity of QS systems outside well-characterized organisms. Expansion of the analysis to ±10 kb reinforced this pattern, as the neighborhood was dominated by monooxygenases, oxidoreductases, transport-related genes, and hypothetical proteins, with no enrichment of genes associated with canonical AHL-QS systems. As such, the genomic neighborhood of this *luxI/R* gene pair in BL16A closely parallels that of the *luxI* homologs identified in the Gram-positive bacteria examined in this study (Figures 1, 2).

The remaining two *luxI/R* loci in BL16A^T^ include *rpaI/R* (BL16A_bacluxhmm_07499 and BL16A_bacluxhmm_07500, respectively) and *lasI/R* (BL16A_bacluxhmm_08326 and BL16A_bacluxhmm_08327, respectively). The *rpaI/R* locus is positioned adjacent to genes involved in carbohydrate metabolism. These include a KdgT permease and a complete KDG utilization pathway (KdgT–KdgK–Eda), which is a central intermediate in the Entner-Doudoroff (ED) pathway and commonly associated with the breakdown of plant-derived polysaccharides. This supports a potential link between AHL-based signaling and carbohydrate scavenging, consistent with the availability of plant cell wall-derived substrates in soil or cave environments. The *rpaR* gene is located 82 bp upstream of the *rpaI* gene, and points to a canonical arrangement with the LuxR regulating the AHL synthase (Hirakawa et al., 2011). The *lasI/R* locus is located 298 bp downstream from a transcriptional regulator annotated as DmlR, which has been implicated in the metabolism of xenobiotic and complex organic compounds (Tropel and van der Meer, 2004). This may link AHL-QS to metabolic pathways involved in environmental substrate utilization. The *lasR* gene is located 92 bp upstream of the *lasI* gene, consistent with a coupled *luxI/R* genomic topology.

### Phylogenetic distribution of LuxI- and LuxR-family proteins

Phylogenetic analyses revealed that LuxI-family proteins formed phyla-specific clusters. Actinomycetota-associated sequences– GV4, Y88A, *S. arenicola*, and all *Streptomyces* spp. included in this analysis–clustered separately, forming a monophyletic clade that shared a more recent common ancestor with the first LuxI-family protein identified in BL16A^T^ (phylum Pseudomonadota; “BL16A^T^ bacluxhmm 02191” described above), than with other bacterial strains (Figures 1, 2). The cognate LuxR-family protein “BL16A^T^ bacluxhmm 02192” is most closely related to *Rhodospirillum rubrum* ATCC 11170 *Rru A3395.* The other two LuxI-family proteins found in BL16A^T^ cluster with other known Alphaproteobacteria AHL synthases. The LuxI-family protein in K61^T^ groups within a Gammaproteobacteria clade that is distinct from Alphaproteobacteria LuxI-family proteins, consistent with previous work showing a long-standing evolutionary separation between Gamma- and Alphaproteobacteria AHL-QS systems (Lerat and Moran, 2004).

Phylogenetic analysis of LuxR-family proteins revealed a clear separation between Actinomycetota- and Pseudomonadota-associated sequences. None of the HTH-only LuxR-family proteins from Actinomycetota clustered with experimentally verified canonical LuxR proteins (Figure 1), with two exceptions: *Streptomyces clavuligerus* (BacLuxHMM_00212) and *Streptomyces coelicolor* (BacLuxHMM_06941). Although both are HTH-only LuxR-family proteins, they grouped within the Pseudomonadota clade, closest to *Agrobacterium tumefaciens* TraR and *Azospirillum lipoferum* AlpR.

Several other HTH-only LuxR-family proteins from Alpha- and Gammaproteobacteria were interspersed within the Actinomycetota clades, resulting in multiple paraphyletic groupings. Three HTH-only LuxR-family proteins from BL16A^T^ (“bacluxhmm_08036,” “bacluxhmm_00759,” and “bacluxhmm_07424”) formed a small paraphyletic clade with HTH-only LuxR-family proteins from *Streptomyces* spp. Additionally four other HTH-only LuxR-family proteins (“BL16A^T^ bacluxhmm 05213”, “BL16A^T^ bacluxhmm 01040”, “BL16A^T^ bacluxhmm 01808”, and “K61^T^ BacLuxHMM 03690”) are branched within a paraphyletic clade that includes HTH-only LuxR-family proteins from all Actinomycetota in this study. The largest paraphyletic clade has a total of 14 HTH-only LuxR-family proteins from BL16A^T^ and K61^T^, as well as *Vibrio campbellii* ATCC BAA-1116 LuxR, a reference LuxR-family protein (Figure 1).

### *N*-acyl homoserine lactone production in four bacteria from underexplored environments

We detected a range of AHL molecules in four bacteria isolates from understudied environments (Table 2). All LC-MRM-MS data are in Supplementary File 1 and have been deposited online in the MassIVE repository (MassIVE #MSV00100312). A representative extracted ion chromatogram (EIC) of AHL standards at 12.5 ng/mL is provided (Supplementary Figure 1).

**Table 2:** AHLs detected by LC-MRM-MS of K61^T^, BL16A^T^, GV4, and Y88A.

Standard (HSL)	*m/z* [M + H]^+^	Retention Time^a^ (t_R_)	K61^T^						BL16A^T^		GV4	Y88A
			GYE			GYE + 10% IIa Brine			PDB	R-2A	R-2A	GYE
			24 h	48 h	72 h	24 h	48 h	72 h	5d	5d	5d	4d
3-oxo-C6	214	3.0	Detected^c^	Detected	–	Detected	Detected	Detected	Detected	–	–	–
C6	200	3.8	Detected	Detected	Detected	–	Detected	Detected	–	–	–	–
C7	214	4.3	–	–	–	–	–	–	–	–	–	–
C8	228	4.6	–	–	–	–	Detected	Detected	Detected	–	–	–
3-oxo-C10	270	4.9	–	–	–	–	–	–	–	–	–	–
C10	256	5.4	–	Detected	Detected	–	–	–	Detected	Detected	–	–
3-oxo-C12	298	5.6	–	–	–	–	–	–	Detected	Detected	Detected	Detected
C12	284	6.1	–	Detected	Detected	–	–	–	–	Detected	–	Detected
3-oxo-C14	326	6.3	–	–	–	–	–	–	–	Detected	–	–
C14	312	6.8	–	–	–	–	–	–	–	Detected	–	–
Unidentified AHL^b^ (t_R_)	–	–	5.0, 6.0, 6.5, 6.8	5.2	5.2	6.5	–	–	3.1, 3.7, 5.4, 7.0, 7.5	–	6.2,7.1	–
Figure	–	–	Figure 6 and Figure 7			Figure 6 and Figure 7			Figure 8		Figure 5	Figure 3
Supplementary material	–	–	Supplementary Figure 2 and Supplementary Files 1, 2						Supplementary Files 3, 4		Supplementary File 5	Supplementary File 6

Listed are AHL standards with corresponding mass-to-charge ratios (*m/z*) and retention times (t_R_), alongside presence or absence of each compound. Detection was confirmed based on matching parent *m/z*, retention time, and the characteristic *m/z =* 102 fragment. Additional peaks consistent with AHLs but lacking standard matches are reported as unidentified AHLs. Growth conditions include glucose yeast extract (GYE), GYE supplemented with 10% MgSO₄ brine (IIa), potato dextrose broth (PDB), and Reasoner’s 2A (R-2A) medium. HSL, homoserine lactone; *m/z*, mass to charge ratio; t_R_, retention time; h, hours; GYE, glucose yeast extract; R-2A; Reasoner’s 2A medium; PDB, potato dextrose broth; IIa, MgSO_4_-rich brine.

^a^Retention times (t_R_) vary slightly from run to run.

^b^Unidentified AHL = provided the characteristic m/z = 102 fragment.

^c^Detected = correct parent m/z, retention time, and characteristic m/z = 102 fragment as standard.

**Figure 5:**
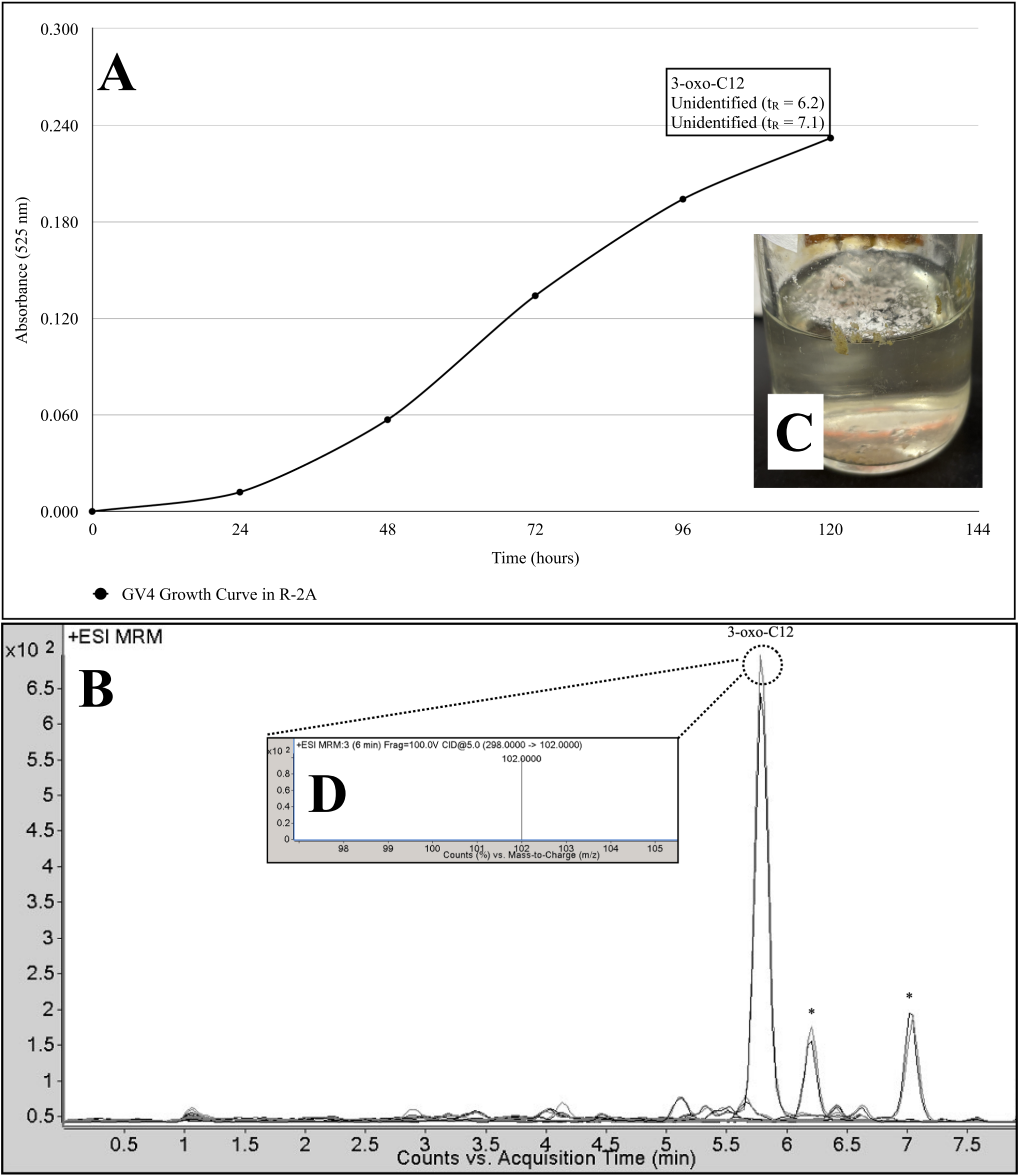
Growth curve **(A)** and chromatogram **(B)** representing *N*-acyl homoserine lactone (AHL) production of *Pseudonocardia alni* GV4 **(C)** in Reasoner's-2A (R-2A) medium. Fragmentation spectra **(D)** shows the parent molecular ion of 3-oxo-C12-HSL (*m/z* = 298) and a fragmentation ion product corresponding to the lactone ring moiety (*m/z* = 102). Please refer to Figure 1 for the representative extracted ion chromatogram (EIC) of AHL standards at 12.5 ng/ml. *=unidentified putative AHL with fragment at *m/z* = 102.

Two Actinomycetota strains evaluated produced AHLs. LC-MRM-MS of culture extracts revealed that *Pseudonocardia alni* GV4 grown in R-2A broth produced 3-oxo-C12-AHL 5 days post-inoculation. At least two putative AHLs were detected, each exhibiting diagnostic fragment ions with *m/z* = 102, consistent with the conserved homoserine lactone ring (Figure 5). These signals had retention times and peak areas that did not correspond to standards used here, and are thus described here as unidentified AHLs. Similarly, when grown in GYE broth for 5 days, *Rhodococcus kroppenstedtii* Y88A produced two longer-chain AHLs, specifically 3-oxo-C12-AHL and C12-AHL (Figure 3). Thick biofilms were observed on the surfaces of these liquid cultures (Figure 4).

**Figure 6:**
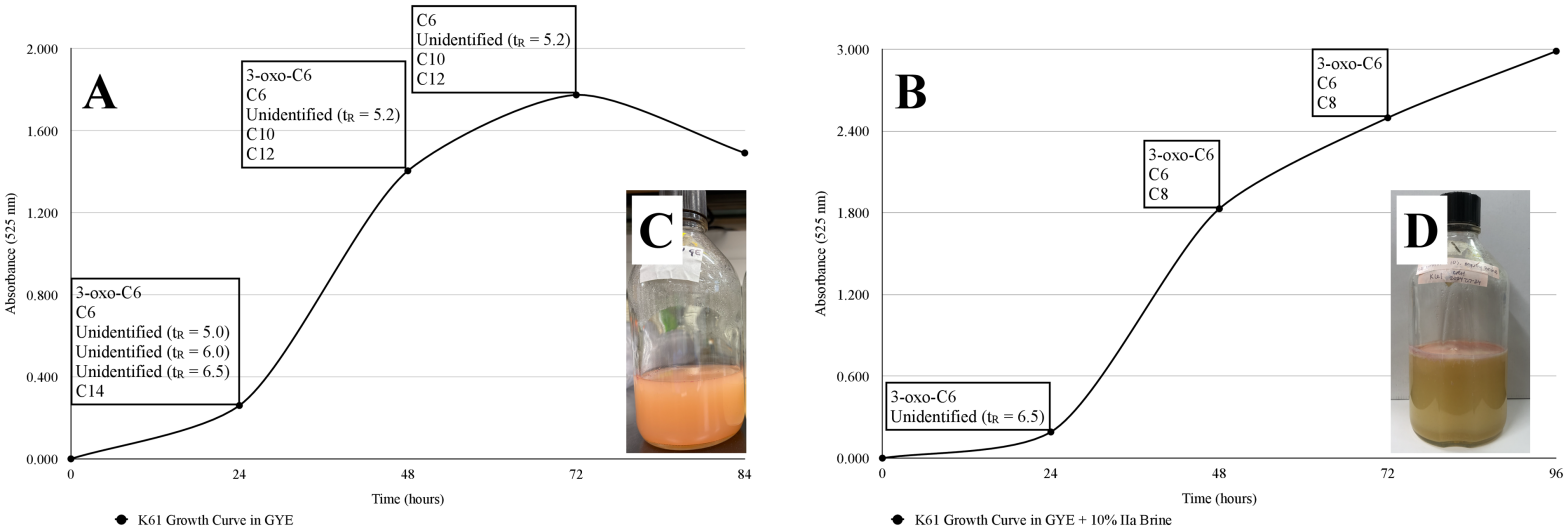
Growth curves of *Brenneria uluponensis* K617 and *N*-acyl homoserine lactone (AHL) production in **(A)** Glucose-Yeast Extract (GYE) and **(B)** GYE + 10% IIa Brine at 24, 48, and 72 h. The growth of K61^T^ was measured by assessing the absorbance of one milliliter of culture at 525 nm using a spectrophotometer. A variety of AHLs were detected at 24, 48, and 72 hours post-inoculation in both media types. Unidentified AHLs are denoted with their retention time (t_R_). **(C)** *B. uluponensis* K61^T^ in GYE. **(D)** *B. uluponensis* K61^T^ in GYE + 10% IIa Hesperian Age Mars Brine. Whereas turbid, pink growth was observed in GYE lacking brine, cultures in 10% IIa brine formed a thick, pink, pungent biofilm at the air-liquid interface one week post-inoculation at room temperature. These observations indicate phenotypic variation in growth across environmental conditions. The loss of longer-chain AHLs in the brine condition further suggests that environmental differences modulate AHL production. Please refer to Supplementary Figures 1, 2 for the representative extracted ion chromatogram (EIC) of AHL standards at 12.5 ng/ml. and all biological replicate traces, respectively.

**Figure 7:**
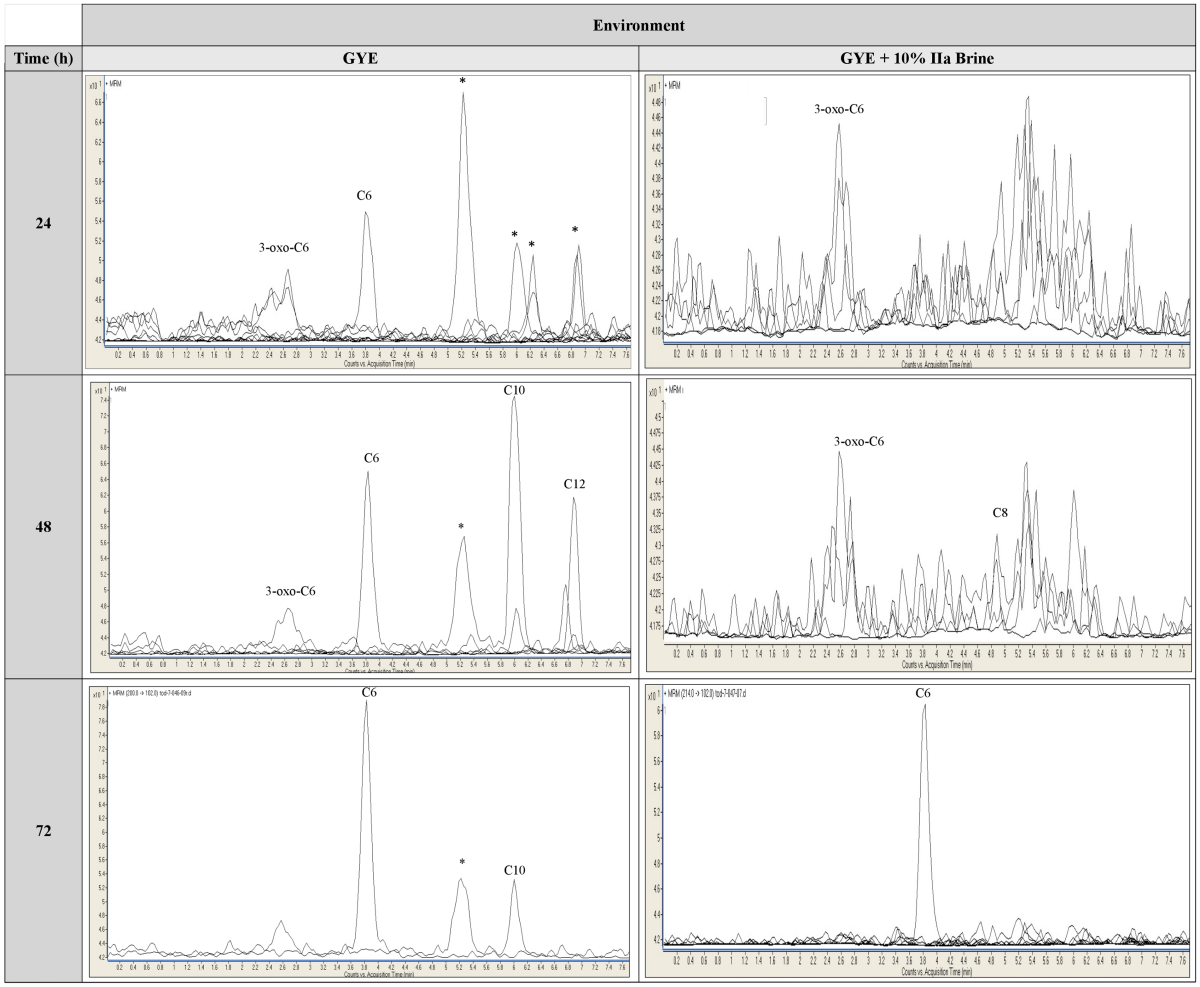
Chromatograms showing *N*-acyl homoserine lactone (AHL) production by *Brenneria uluponensis* K61^T^ in Glucose-Yeast Extract (GYE) broth and in GYE broth supplemented with 10% IIa brine at 24, 48, and 72 h. Long-chain AHLs are reduced under Mars brine conditions. The *y*-axis represents ion detection frequency at each time point, and the scale varies considerably, particularly in the 24- and 48-hour Mars brine samples. The generally accepted criteria for chemical detection are defined by signal-to-noise (S/N) ratios, with limits of quantification set at 10× S/N and limits of detection at 3× S/N; the proximity of observed signals to these thresholds impacts the strength of any conclusions. Data from one biological replicate are displayed. See Supplementary Figures 1, 2 for representative extracted ion chromatograms (EICs) of AHL standards (12.5 ng/ml) and for traces from all biological replicates, respectively. * = unidentified putative AHL with fragment at *m/z* = 102.

In the Pseudomonadota *Brenneria uluponensis* K61^T^, LC-MRM-MS revealed production of 3-oxo-C6-AHL in 24 and 48 h cultures (exponential phase) in GYE and in GYE supplemented with 10% MgSO_4_ brine (Figures 6, 7 and Supplementary Figure 2). AHL production declined by 72 h, coinciding with entry to the stationary phase (Figures 6, 7 and Supplementary Figure 2). Growth in the MgSO_4_ brine-amended medium was slower than in GYE, and cultures formed a thick, pink biofilm at the liquid-air interface (Figure 6). Comparison of AHL profiles in different growth conditions revealed distinct AHL production profiles at different times (Figures 6, 7 and Table 2). During exponential growth, K61^T^ produced C6-AHL and 3-oxo-C6-AHL in both media, but at 24 h in GYE broth, four additional AHLs were detected, with two more putative, unidentified AHLs at 48 and 72 h (late exponential to stationary phase). Notably, C10-AHL and C12-AHL were produced only in GYE broth (Figures 6, 7). Fewer AHLs among the suite we could detect were found in the MgSO_4_ brine-amended medium, with only C6-AHL, 3-oxo-C6-AHL, and one unidentified AHL detected early in the incubation, while C8-AHL appeared later (48–72 h) in the incubation. The latter was not detected in the unamended GYE with K61^T^ (Figures 6, 7).

**Figure 8:**
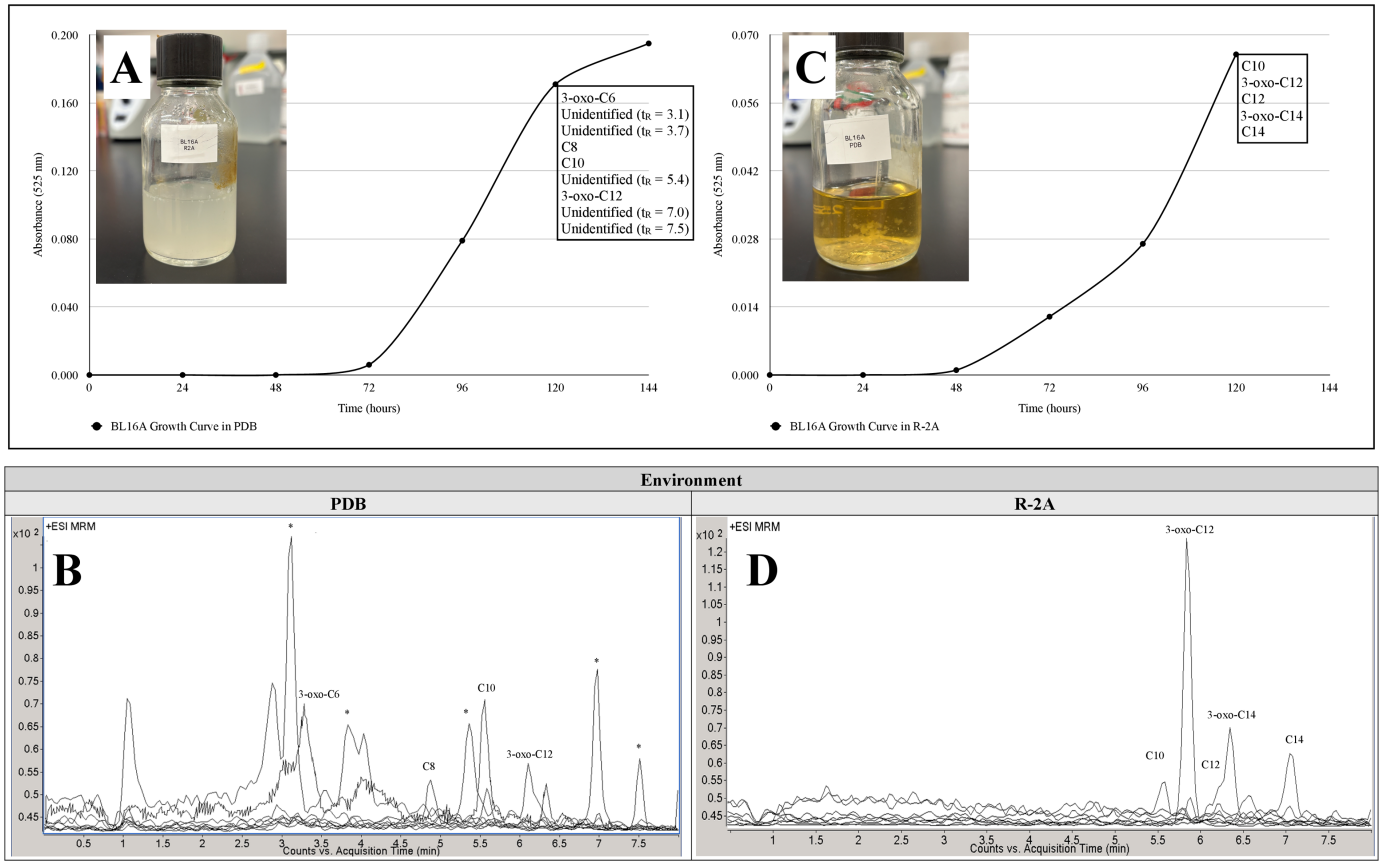
Growth curves and chromatograms representing *N*-acyl homoserine lactone (AHL) production of *Bradyrhizobium prioriisuperbiae* BL16A^T^ in **(A,B)** nutritionally rich Potato Dextrose Broth (PDB) and **(C,D)** nutritionally poor Reasoner's-2A (R-2A) broth. Please refer to Figure 1 for the representative extracted ion chromatogram (EIC) of AHL standards at 12.5 ng/ml. *=unidentified putative AHL with fragment at *m/z* = 102.

The AHLs detected in the Pseudomonadota *Bradyrhizobium prioriisuperbiae* BL16A^T^ also varied by growth medium. After 5 days in PDB, a nutrient-rich medium, nine known AHLs were detected, including 3-oxo-C6-AHL, C8-AHL, C10-AHL, and 3-oxo-C12-AHL, along with five unidentified AHLs with *m/z* = 102 (Figure 8 and Table 2). In contrast, cultures grown in R-2A, a nutrient-poor medium, produced at least five AHLs, including C10-AHL, 3-oxo-C12-AHL, C12-AHL, 3-oxo-C14-AHL, and C14-AHL (Figure 8 and Table 2).

## Discussion

The QS research has historically emphasized medically relevant and host-associated bacteria, leaving environmental systems comparatively underexplored (Whiteley et al., 2017). Our results add to the growing body of literature documenting the phylogenetic diversity of AHL-producing organisms and further demonstrate that AHL production does not solely occur in Pseudomonadota or in Gram-negative *Bacteria*. In this study, we provide experimental evidence that Actinomycetota synthesize AHLs using a *luxI* homolog, supporting recent bioinformatic studies that have identified *luxI/R* genes in Gram-positive bacterial genomes. To date, only 30 *luxI* genes have been experimentally verified through gene deletion or disruption experiments in the UniProt database, with all but one belonging to Pseudomonadota. Thus, our work represents the first investigation of an AHL synthase in Actinomycetota and in *Rhodococcus*.

In addition, we show that *luxI* genes in Actinomycetota occur within genomic contexts distinct from canonical Pseudomonadota QS systems and are frequently associated with genes involved in sulfur, redox, or related metabolic processes. Notably, while both Actinomycetota examined produced AHLs and have *luxI* homologs, none of their LuxR-family proteins contained detectable ABDs despite universal retention of the conserved HTH DNA-binding domain. This architectural divergence raises fundamental questions about if and how AHL signals are perceived in these organisms, including whether Actinomycetota produce AHLs without directly sensing them, rely on non-canonical LuxR-family proteins for sensing, or employ alternative signal reception mechanisms altogether. Collectively, these findings challenge simplified models of AHL-QS as a tightly coupled, one-signal, one-receptor system and suggest that AHL signaling in Actinomycetota may play roles that differ from those classically observed in Gram-negative bacteria. Key results are discussed further below.

### Genomic context, multifunctionality, and experimental evidence of *luxI* solos in Gram-positive bacteria

In this study, we (1) provide experimental evidence that Actinomycetota synthesize AHLs, (2) demonstrate that Actinomycetota have single *luxI* genes (solos) that are not co-located with *luxR* genes, and (3) reveal that the genomic neighborhoods surrounding *luxI* genes is different for Actinomycetota compared to Pseudomonadota. The two Actinomycetota in this study, *Pseudonocardia alni* GV4 and *Rhodococcus kroppenstedtii* Y88A, are the third and fourth known Actinomycetota, and the fourth and fifth known Gram-positive bacteria to synthesize AHLs (Biswa and Doble, 2013; Bose et al., 2017). Using an in-house HMM, a single *luxI* gene was identified in each Actinomycetota genome, suggesting that these genes may be solely responsible for the production of multiple AHLs observed in these species. Because our LC–MS analyses were targeted to a defined set of standards, we likely underestimated the full diversity of AHLs produced by these strains. The observation of multiple AHLs could reflect synthesis via atypical biosynthetic pathways (Duddy and Bassler, 2021). The presence of additional AHL-related genes that were not detected by our HMMs is possible due to the limited taxonomic representation of experimentally verified *luxI* genes outside the Pseudomonadota.

Gram-positive bacteria lack the canonical *luxI/R* gene arrangements commonly observed in Gram-negative systems. In particular, LuxR-family proteins containing ABDs were absent within ±10 kb of the *luxI* loci, consistent with recent reports of atypical *luxI/R* organization across diverse bacterial taxa (Cai and Zhang, 2024). Although HTH-type transcriptional regulators were occasionally present in these regions, their lack of detectable ABDs is more consistent with local transcriptional regulation or integration within metabolic gene clusters than with classical QS circuitry. These patterns are consistent with Gram-positive *luxI* homologs functioning as metabolically integrated enzymes. It is possible that AHL production in these organisms may serve roles distinct from, or only partially analogous to, classical population-density sensing systems characterized in Gram-negative bacteria.

*luxI* homologs in GV4 and Y88A are both co-localized with genes involved in tRNA modification, sulfur metabolism, and ETFs. Recent work has demonstrated that QS can modulate electron transfer and redox-associated processes, suggesting that QS-regulated behaviors in Actinomycetota may intersect with cellular metabolic state (Qu et al., 2025). These observations raise the possibility that AHL synthesis in GV4 and Y88A is linked to a redox-metabolic context. Similar patterns were observed in *Streptomyces*, where *luxI* homologs are frequently located near genes involved in redox balance and secondary metabolism, including flavin-dependent monooxygenases and other oxidoreductases. The repeated association of *luxI* homologs with redox-active metabolic genes suggests a conserved genomic linkage, although the regulatory roles of AHL production in Gram-positive bacteria remain unresolved.

The *luxI* homolog in the *Salinispora* sp. was unique in that it was flanked by genes associated with environmental sensing and stress response. The absence of biosynthetic enzymes or canonical LuxR-family proteins suggests that AHL production by the *luxI* homolog may be integrated into broader environmental response networks rather than classical QS architectures. This genomic arrangement is notable given the marine origin of *Salinispora* and its placement within an evolutionary ancient Actinomycetota lineage (Tischler et al., 2019). This may reflect conserved strategies for AHL-signaling under fluctuating environmental conditions, such as those encountered in marine environments.

Finally, targeted disruption of the sole *luxI* homolog in Y88A resulted in a complete loss of detectable AHL production in two independent mutants, indicating that this gene is required for AHL production under the tested conditions. As the genome contains only a single member of the highly conserved *luxI* gene family, the likelihood that an alternative AHL synthase gene was overlooked is low. Together, these findings suggest that this gene functions as an AHL synthase, consistent with bioinformatic prediction. We therefore designate this gene *rhdI*, representing the first experimentally supported *luxI* homolog described in the genus *Rhodococcus*. Although *Rhodococcus* are well known for quorum-quenching capabilities (Ryu et al., 2020), this study provides the first experimental evidence for AHL-QS within the genus. While genetic complementation would further strengthen this result, such methods remain challenging in non-model organisms such as *R. kroppenstedtii* Y88A due to limited genetic methods for non-model organisms, and was therefore not completed. However, complementation is not necessary to conclude that the *rhdI* is required for AHL production under the tested conditions, as no AHLs were detected by two mutant strains under the same growth conditions.

### Phylogenetic distribution and architecture of LuxI- and LuxR-family proteins

Phylogenetic analyses show that AHL-QS systems span multiple, evolutionarily distinct lineages. LuxR-family proteins from Gram-negative bacteria are generally clustered within well-defined clades corresponding to established AHL-QS systems, consistent with their conserved canonical, ABD and HTH domain architecture. In contrast, LuxR-family proteins from Actinomycetota formed multiple distinct and deeply branching clades, reflecting substantial evolutionary divergence. This is consistent with prior investigations that included study of AHL-QS in Actinomycetota (Subramoni et al., 2015). Notably, several LuxR homologs from Alphaproteobacteria and Gammaproteobacteria were interspersed among Actinomycetota-dominated clades, a pattern that may reflect ancient diversification or repeated horizontal gene transfer events. These data suggest that AHL-QS systems in Gram-positive bacteria may be more widespread and diverse than currently recognized. Further investigation of environmental bacteria will be essential to clarify the roles and evolutionary trajectories of AHL-based signaling outside well-studied lineages.

Despite phylogenetic intermixing, none of the LuxR-family proteins identified from Gram-positive bacteria in this study contained detectable ABDs. These data may support a single evolutionary scenario, such as widespread loss of the ABD, but we suggest that these data are simply consistent with diversification within the LuxR family, in which the conserved HTH DNA-binding domain is present while sensory architectures vary. This pattern suggests that many LuxR-family proteins in Actinomycetota may function as transcriptional regulators that are not directly responsive to AHLs, but further study is required to determine this. In Gram-positive bacteria, AHL production may occur independently of a paired LuxR-family receptor. Such architectures may represent vestigial or repurposed forms, although further experimental work is needed to test these possibilities.

The genome of *P. alni* GV4 encodes an unusually large number of HTH-only LuxR-family proteins, none of which cluster with canonical LuxR-family proteins. This diversity suggests that GV4 harbors a broad and largely uncharacterized repertoire of LuxR solos, a pattern previously reported in Actinomycetota and thought to reflect expanded regulatory capacity or responsiveness to diverse environmental cues. Although several *luxR* genes are located upstream of the *luxI* gene in GV4, the large intergenic distances separating these loci make it unknown as to whether they are cognate *luxI/R* pairs. Experimental validation or transcriptional data would be required to determine whether any of these LuxR-family proteins interact with the *luxI* genes. It is also possible that Actinomycetota do not engage in classical “self-talk,” but instead produce AHLs primarily for interspecies signaling without sensing AHL signals. This would, however, incur an energetic cost, as signaling molecules would be synthesized without an apparent local LuxR-mediated response; therefore, further research is needed to clarify why Actinomycetota produce AHLs in the absence of proximal LuxR regulation.

In contrast to LuxR-family proteins, LuxI-family proteins are relatively conserved and can synthesize approximately 20 known AHL molecules, with many more AHL types likely found in nature. Current databases contain more than 3,600 LuxR-family homologs, many of which are classified as solos or “orphans,” lacking an associated LuxI-family synthase. As proposed previously (Prescott and Decho, 2020), this may suggest that *luxR* genes are more easily adapted to various environmental conditions, including what other species are in the local area and what signals they are producing. This would allow for flexibility to AHL-QS systems in nature, allowing bacteria to respond to changing environments and shifting community composition.

Finally, it remains possible that many *luxR* genes in Actinomycetota are not directly involved in AHL-QS at all. The apparent decoupling of signal detection from DNA binding highlights an evolutionary divergence in QS strategies and expands the functional landscape of LuxR-family proteins beyond canonical AHL-based systems. The presence of an HTH domain alone is therefore insufficient to infer AHL responsiveness, particularly in Gram-positive bacteria. Instead, the observed architectural diversity suggests that LuxR-family proteins participate in a wide range of regulatory roles.

### Environmental modulation of AHL production

AHL profiles varied across growth conditions, consistent with prior studies showing that environmental context influences AHL production (Pongsilp et al., 2005; Decho et al., 2011; Calatrava-Morales et al., 2018). Together, these shifts in AHL profiles reflect broader physiological or metabolic adjustments to environmental stress, including the synthesis of AHLs by K61^T^ that may be more stable under high ionic and osmotic stress, such as those found in environments characterized by high salinity or altered ionic composition. In BL16A^T^, AHL production shifted toward longer-chain signals under more oligotrophic or stress-like conditions, a pattern that may reflect adaptations to lava caves, a nutrient-poor environment. These shifts in AHL profiles highlight the plasticity of AHL-QS signaling in response to environmental conditions and suggest that laboratory studies conducted under a single growth condition may substantially underestimate the diversity of signals produced by a given strain. This has implications for future research on AHL production, as results may be implicitly biased based on culture media, highlighting the importance of conducting multiple independent experiments to gain a comprehensive understanding of AHL production within a single strain.

The AHLs detected in this study were limited by the 10 standards used in LC-MRM-MS. Although it is standard practice in AHL studies, traditional extraction and mass spectrometry analysis methods using a triple quadrupole mass spectrometer may also underestimate the full diversity of AHLs produced by a single strain. Using additional AHL standards, scanning analyses, or higher-sensitivity metabolite and lipid profiling instruments such as the Orbitrap would likely reveal additional AHLs (Hu et al., 2005). Due to this limitation, we have intentionally taken a conservative approach and do not claim structural identification of signals beyond those that can be confidently assigned based on retention time and the diagnostic transition *m/z* = 102. Signals that could not be resolved to known standards are explicitly reported as putative and discussed accordingly.

### Implications for AHL diversity and signaling complexity

Genomic redundancy reflects the capacity of genes to serve multiple roles, enabling cells to repurpose existing functions across different contexts. Such multifunctionality can be more energy efficient than maintaining entirely separate genetic pathways, allowing the cell to economize its resources. In the context of AHL-QS, a single *luxI* gene may produce multiple AHLs under different environmental conditions, or alternative, non-canonical pathways may exist for AHL synthesis. Together, these features suggest that overlapping regulatory roles can provide both flexibility and resilience in bacterial signaling systems.

Some Bacteria may have duplications of genes or operons, including *luxR* genes (Subramoni et al., 2015). Gene duplications could lead to multiple versions of genes that respond differently to environmental cues, resulting in a more flexible or nuanced QS system (Kondrashov, 2012). Duplicated genes or operons may play specialized roles depending on an ecological niche, even if they originated from the same ancestral gene (Santos et al., 2012). In the case of AHL-QS, perhaps some AHLs bind to specialized LuxR proteins and activate functions like biofilm formation, while others might regulate functions like motility in nutrient-rich environments, or nodulation in soils with decreased availability of soil nitrate. Gene duplication adds layers to the “language” of bacterial communication, with different LuxRs and AHLs “speaking” to different cellular responses based on the environmental conditions. AHL diversity not only provides environmental adaptation but also enables fine-tuned cellular responses via specialized *luxR* genes. This discussion about gene duplication is intended to explore the diversity and flexibility of AHL-based QS systems, not to assert a strict one-to-one mapping between AHL molecules and LuxR receptors. While HGT is indeed a mechanism for the acquisition of QS systems, gene duplication has also been proposed as a means of generating functional divergence within signaling systems (Subramoni et al., 2015; Kondrashov, 2012). Multiple mechanisms, potentially including HGT and gene duplication, may contribute to the complexity observed in LuxI/R diversity, and further research is required.

Looking forward, it is relevant to address why a single bacterial strain might produce a diversity of AHLs, each under different environmental conditions. Classically, AHL-mediated QS is understood to occur once a threshold concentration is reached for a particular AHL, which then activates a specific regulatory cascade controlling functions such as bioluminescence, biofilm formation, or virulence. However, when a bacterium produces, for example, 12 different AHLs, does it also encode 12 corresponding *luxR* receptors, each tuned to recognize one of these AHLs and to regulate distinct sets of genes? Or, alternatively, might AHLs act more broadly as environmental sensors whose production varies with external conditions, collectively shaping the bacterium’s adaptive response? If the latter is true, it suggests that AHL-based signaling may resemble a sensory system, akin to taste, where receptors can recognize several signal molecules to varying degrees, producing patterns of activation that conveys the cell’s perception of its environment. This implies a rather complicated language used by bacteria (with no neuronal processing required).

### Evolutionary implications and unresolved questions in Gram-positive AHL-QS

The Actinomycetota phylum is ancient, is one of the largest and most diverse groups within the domain Bacteria, and contains mostly Gram-positive taxa with high G + C content (Gao and Gupta, 2012). Phylogenetic studies support a common ancestor for the Actinomycetota, Cyanobacteria, and Deinococcus lineages, with the Actinomycetota diverging from all other bacteria some 2.7 Ga, predating the rise of molecular oxygen in Earth’s atmosphere (Battistuzzi et al., 2004). Actinomycetota are ubiquitous in soils, aquatic, and host-associated habitats. They are functionally important in complex, multi-species communities due to their metabolic flexibility and physiological potential (Lewin et al., 2016). Actinomycetota are also known to be highly abundant in volcanic environments (Prescott et al., 2022). This underscores their metabolic flexibility and ecological importance, and these ancient lineages may therefore harbor AHL-QS systems that represent primitive or foundational forms of chemical communication in bacteria. Actinomycetota are an older phylum than Pseudomonadota; these genes may represent an earlier evolution of AHL-QS.

The phylogenetic relationships of LuxI- and LuxR-family proteins highlight that AHL-QS systems in Actinomycetota form distinct evolutionary lineages separate from Pseudomonadota. It is possible that AHL-QS in Gram-positive bacteria may represent ancient or early-diverging forms of chemical communication, however this requires further research. The clustering of certain LuxI- and LuxR-family proteins and the Pseudomonadota homologs presence within the Actinomycetota clade points to potential gene mobility and HGT events that may have shaped AHL-QS evolution. These patterns reinforce the idea that current bioinformatic approaches that are biased toward Pseudomonadota likely underestimate AHL-QS diversity in other phyla. More broadly, the phylogenetic distinctiveness of Actinomycetota AHL-QS genes supports the notion that these bacteria possess unique regulatory strategies, enabling flexible responses to environmental conditions and perhaps contributing to their ecological success across diverse habitats on Earth. Ultimately, these findings highlight both the ancient origins and evolutionary plasticity of AHL-QS systems, while illuminating substantial gaps in our understanding of bacterial communication outside of well-studied lineages.

## Conclusion

Our findings underscore the ancient and potentially under-appreciated role of Actinomycetota in AHL signaling and highlight critical gaps in our understanding of QS evolution, particularly the LuxR-family proteins. This study further expands the known diversity of AHL-QS beyond Gram-negative bacteria and demonstrates that environmental conditions directly influence AHL production. Future research that incorporates community-level analyses, non-canonical gene discovery, and functional studies will be essential to fully capture the diversity and ecological significance of AHL-QS.

## Data Availability

The LC-MRM-MS data presented in the study are deposited in the MassIVE repository (MassIVE #MSV00100312). Genomic metadata (GenBank accession numbers, BioSample, and BioProject identifiers) are as follows: *Brenneria uluponensis* K61^T^ (NZ_CP135917, SAMN37483218, PRJNA1019399), *Bradyrhizobium prioriisuperbiae* BL16A^T^ (NZ_CP135921, SAMN37483212, PRJNA1019399), *Pseudonocardia alni* GV4 (NZ_JARWLD000000000, SAMN34135175, PRJNA954151), and *Rhodococcus kroppenstedtii* Y88A (NZ_CP135915, SAMN37483221, PRJNA1019399. The datasets presented in this study can be found in online repositories. The names of the repository/repositories and accession number(s) can be found in the article/Supplementary material.

## Footnotes

1
https://hmmer.org/
