# Supplementary material for: N-acyl-homoserine lactone-based quorum sensing beyond canonical lineages: insights from Actinomycetota
Source: Front Microbiol. 2026 Apr 20;17:1738013. doi: 10.3389/fmicb.2026.1738013 (PMC13136126; doi:10.3389/fmicb.2026.1738013)
Supplement: Supplementary file 6 [file Data_Sheet_6.pdf]

Qualitative Analysis Report

**Data Filename**	LB-MS-1-005-2.d	**Sample Name**	LB-MS-1-005-2
**Sample Type**	Sample	**Position**	Vial 2
**Instrument Name**	Instrument 1	**User Name**	
**Acq Method**	AHL_MRM2.m	**Acquired Time**	10/21/2022 8:55:36 PM
**IRM Calibration Status**	Not Applicable	**DA Method**	B&S_trial_02232021.m
**Comment**			

User Chromatograms

Fragmentor Voltage 100 **Collision Energy** 5 **Ionization Mode** ESI

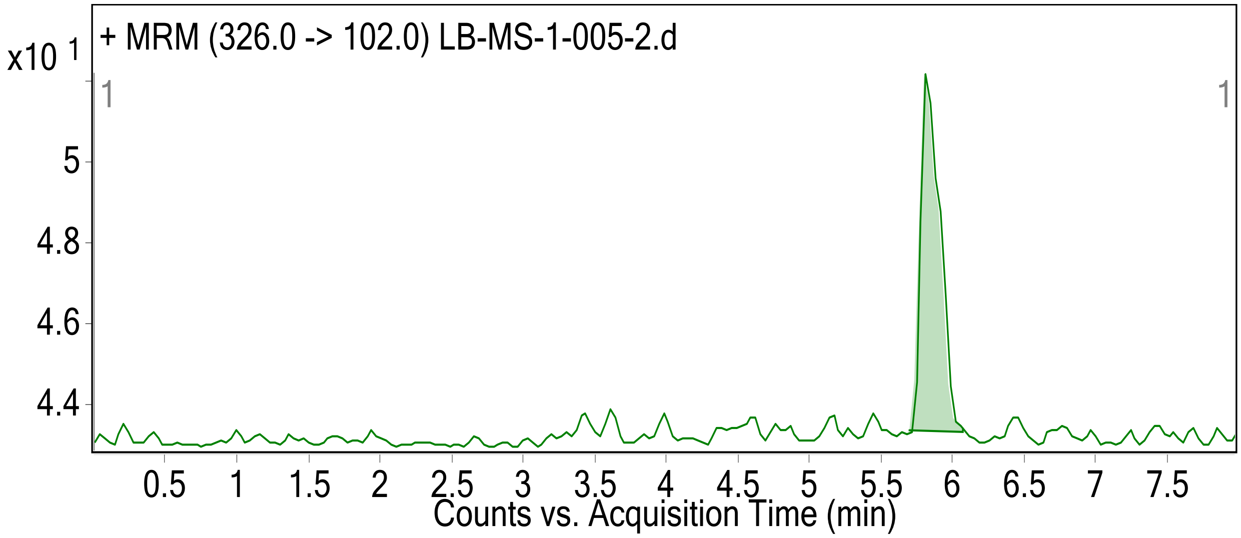

Integration Peak List

Peak	Start	RT	End	Height	Area	Area %
1	5.7	5.82	6.08	9	83	100

Fragmentor Voltage 100 **Collision Energy** 5 **Ionization Mode** ESI

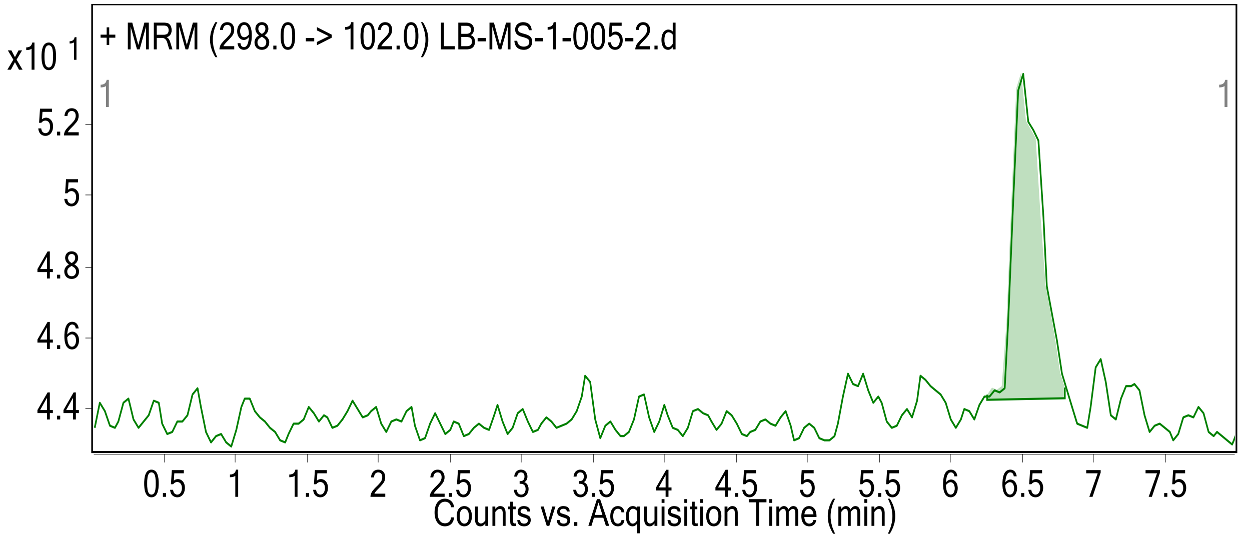

Integration Peak List

Peak	Start	RT	End	Height	Area	Area %
1	6.25	6.48	6.79	9	128	100

Fragmentor Voltage 100 **Collision Energy** 5 **Ionization Mode** ESI

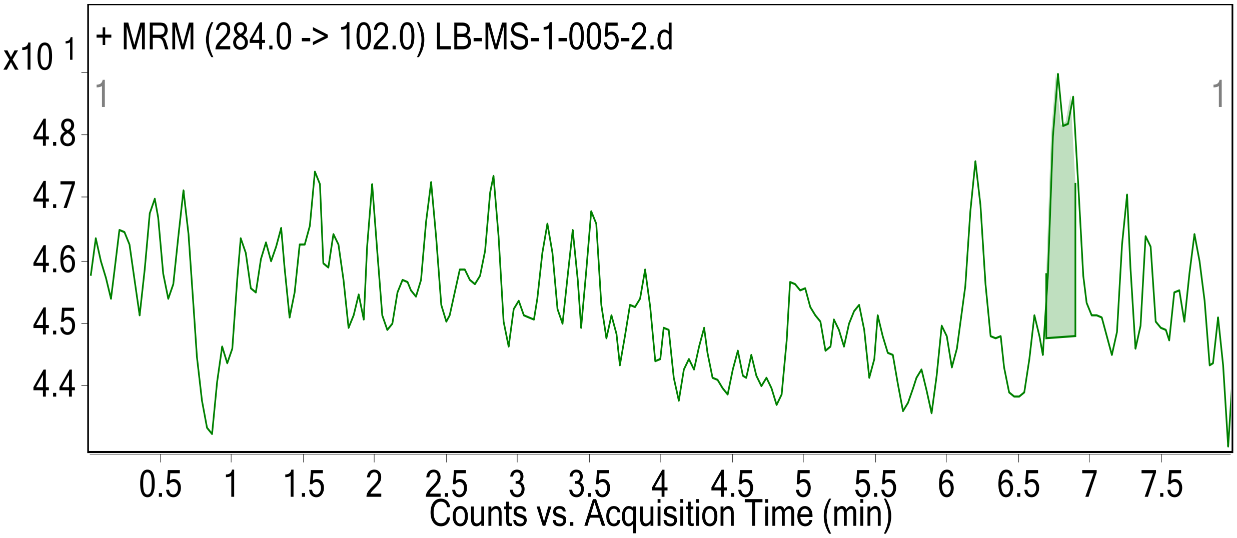

Integration Peak List

Peak	Start	RT	End	Height	Area	Area %
1	6.7	6.77	6.9	4	44	100

Fragmentor Voltage 100 **Collision Energy** 5 **Ionization Mode** ESI

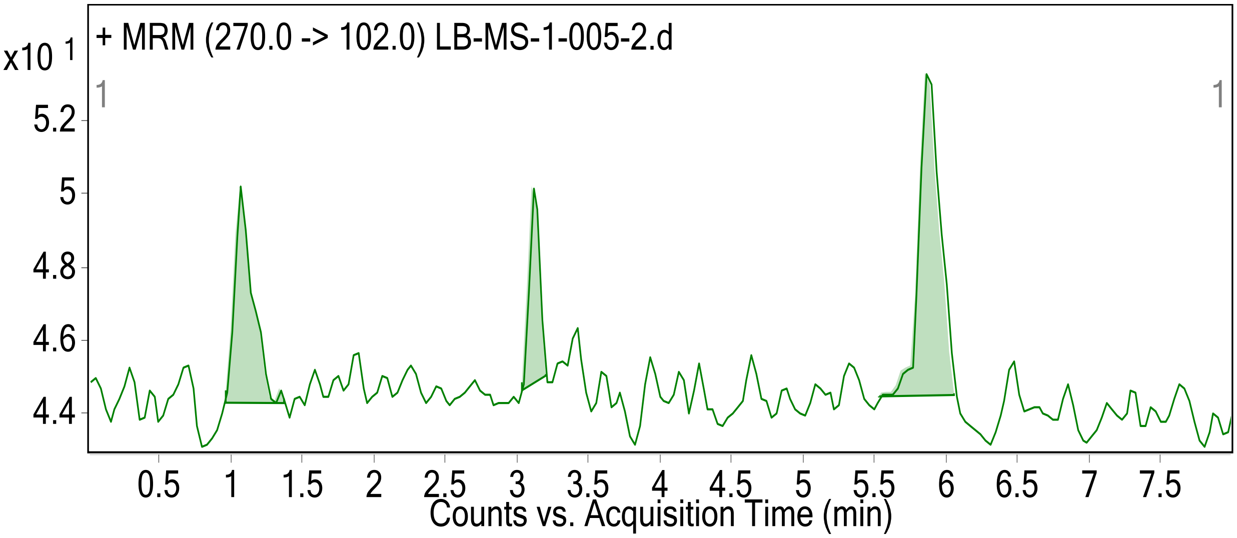

Integration Peak List

Peak	Start	RT	End	Height	Area	Area %
1	0.96	1.07	1.37	6	55	60.85
2	3.03	3.11	3.2	5	29	31.97
3	5.53	5.86	6.05	9	90	100

Fragmentor Voltage 100 **Collision Energy** 5 **Ionization Mode** ESI

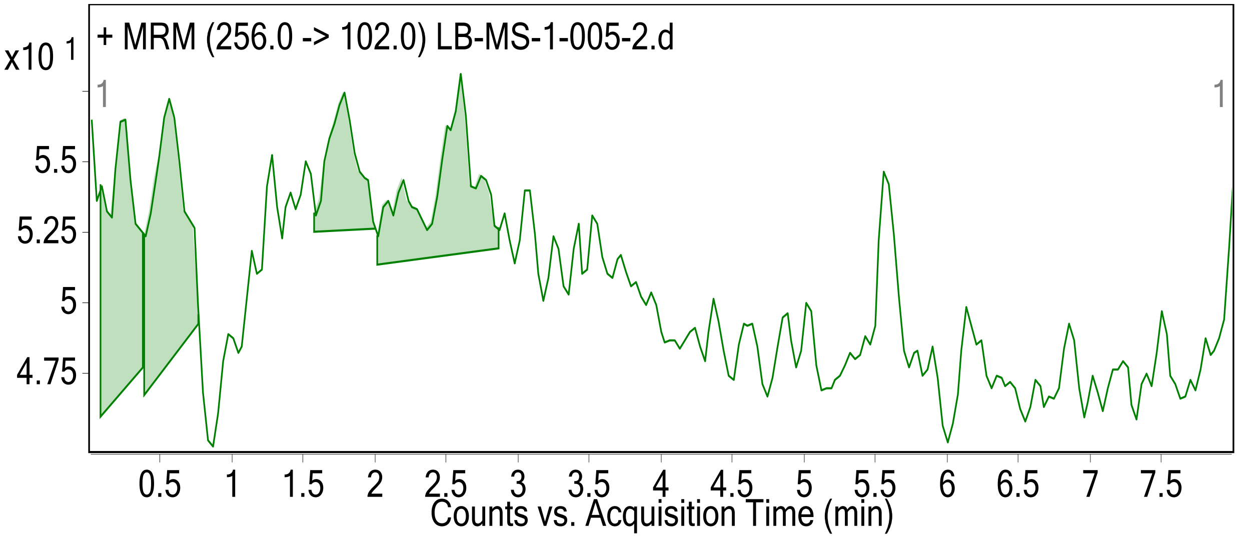

Integration Peak List

Peak	Start	RT	End	Height	Area	Area %
1	0.08	0.24	0.38	9	139	99.61
2	0.39	0.56	0.77	9	139	100
3	1.58	1.78	2.01	5	68	49.02
4	2.02	2.6	2.87	6	134	96.27

Fragmentor Voltage 100 **Collision Energy** 5 **Ionization Mode** ESI

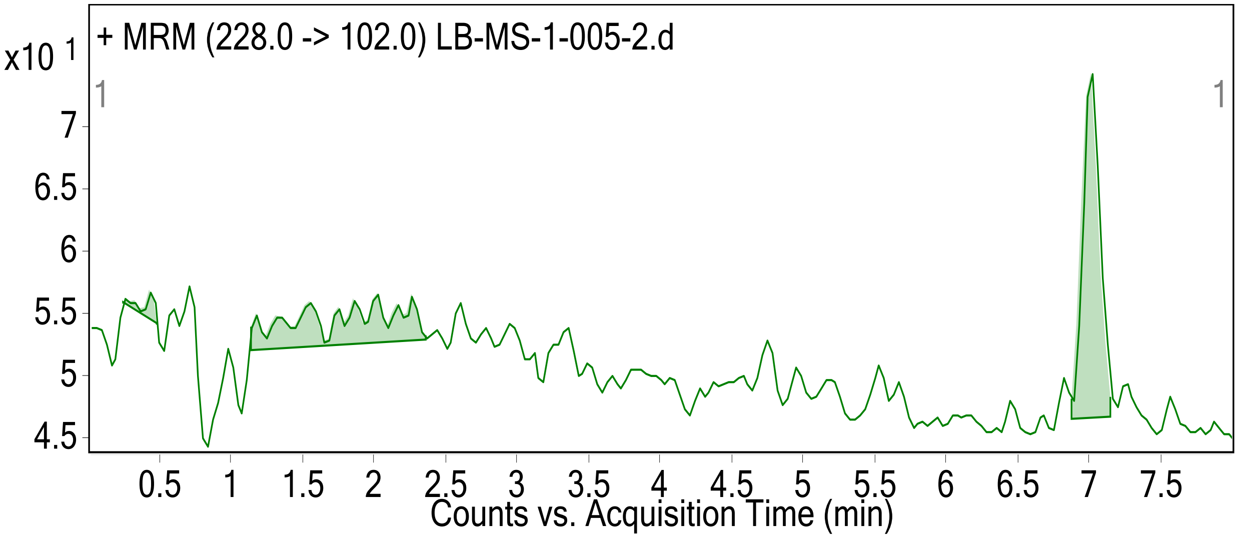

Integration Peak List

Peak	Start	RT	End	Height	Area	Area %
1	0.25	0.43	0.48	2	13	5.33
2	1.14	2.01	2.36	4	169	69.39
3	6.88	7	7.15	28	243	100

Fragmentor Voltage 100 **Collision Energy** 5 **Ionization Mode** ESI

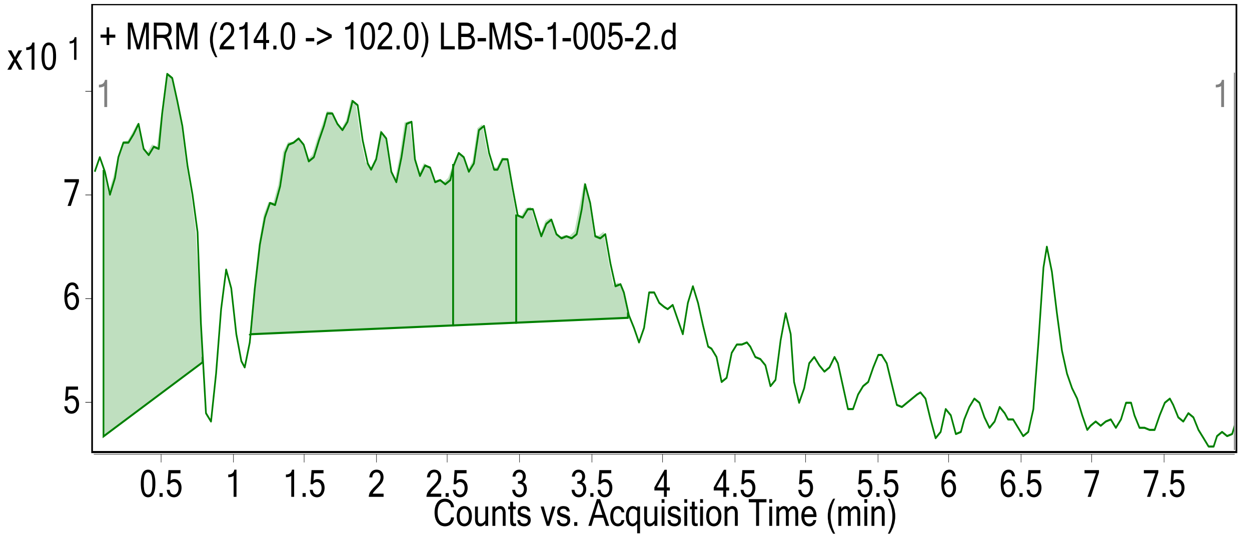

Integration Peak List

Peak	Start	RT	End	Height	Area	Area %
1	0.09	0.55	0.78	30	1003	71.39
2	1.11	1.83	2.54	22	1405	100
3	2.54	2.73	2.98	19	432	30.76
4	2.98	3.45	3.76	13	392	27.91

Fragmentor Voltage 100 **Collision Energy** 5 **Ionization Mode** ESI

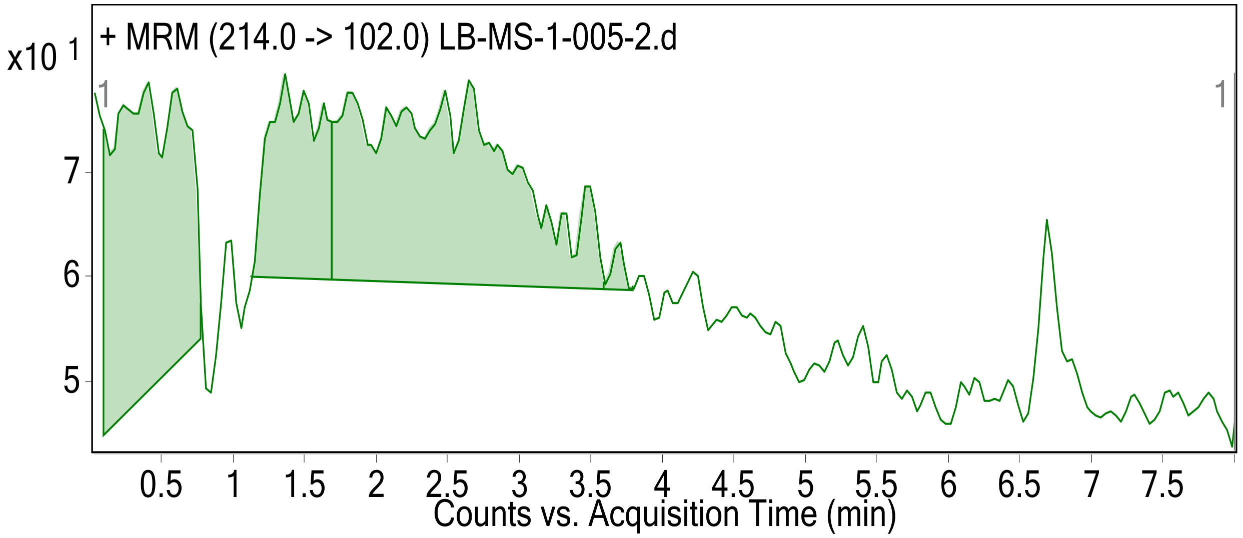

Integration Peak List

Peak	Start	RT	End	Height	Area	Area %
1	0.1	0.39	0.77	29	1031	72.2
2	1.13	1.35	1.69	20	489	34.23
3	1.69	2.65	3.59	20	1429	100
4	3.59	3.68	3.79	5	29	2.05

Fragmentor Voltage 100 **Collision Energy** 5 **Ionization Mode** ESI

Qualitative Analysis Report

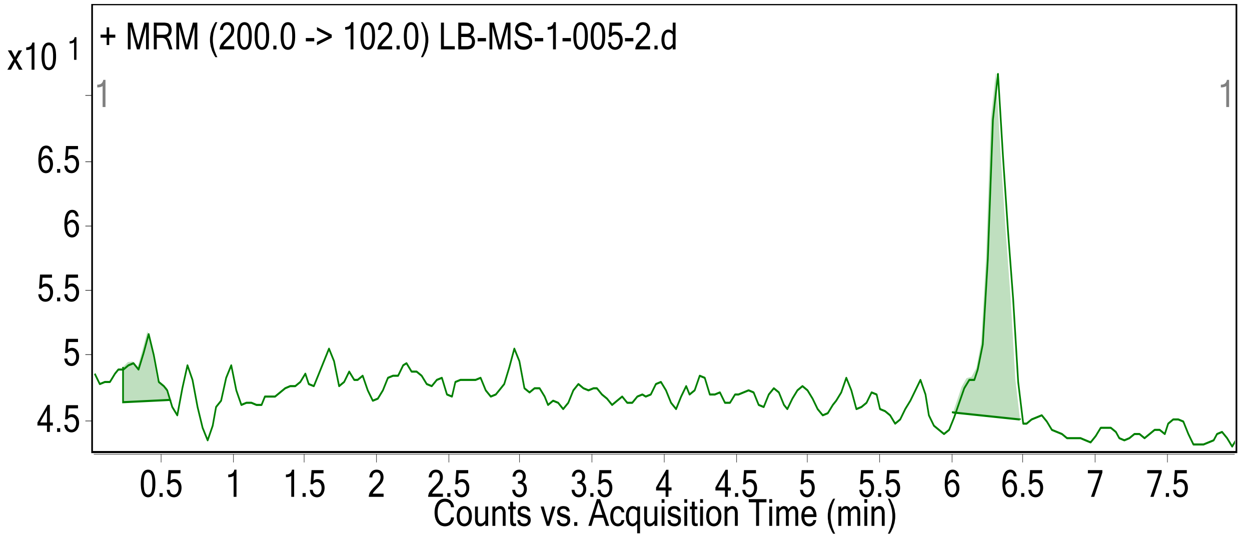

Integration Peak List

Peak	Start	RT	End	Height	Area	Area %
1	0.23	0.4	0.56	5	57	21.99
2	6.01	6.3	6.48	27	259	100

Fragmentor Voltage 100 **Collision Energy** 5 **Ionization Mode** ESI

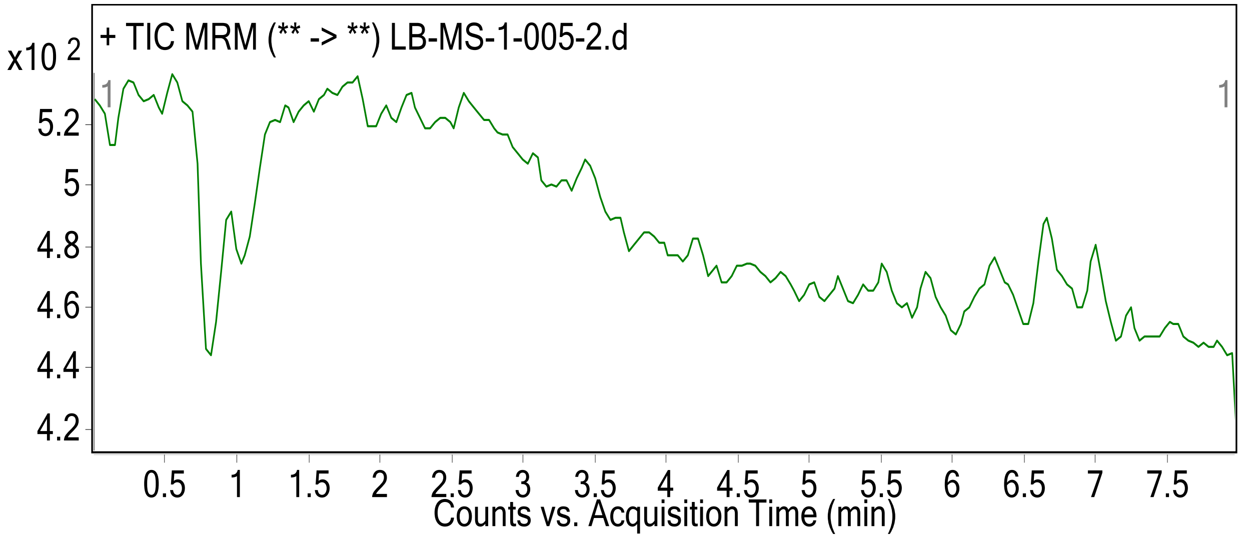

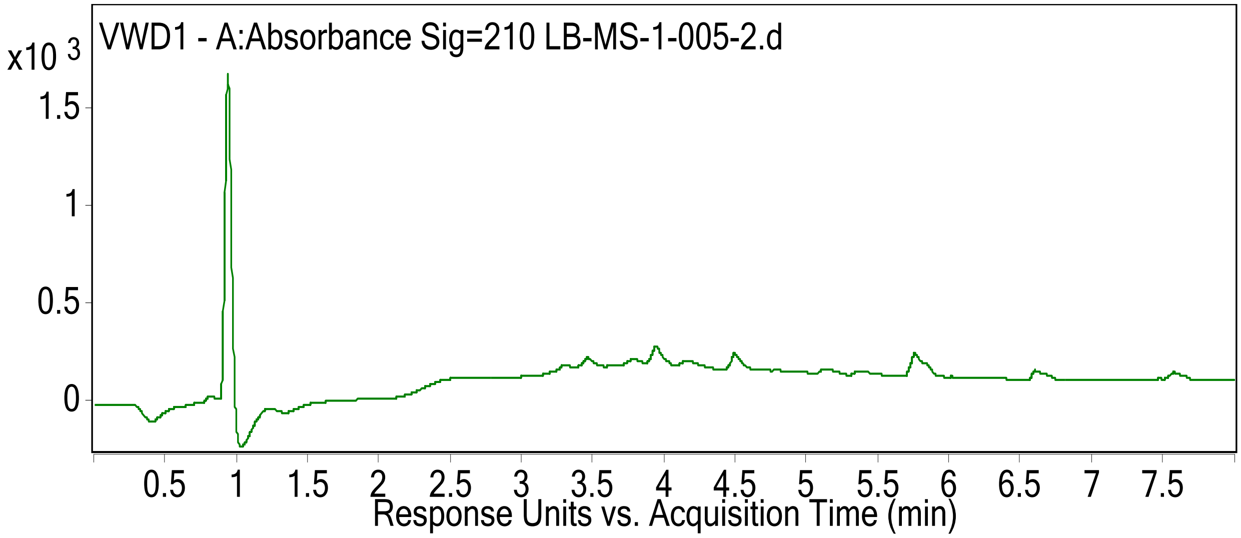

Fragmentor Voltage	100	Collision Energy	5	Ionization Mode	ESI

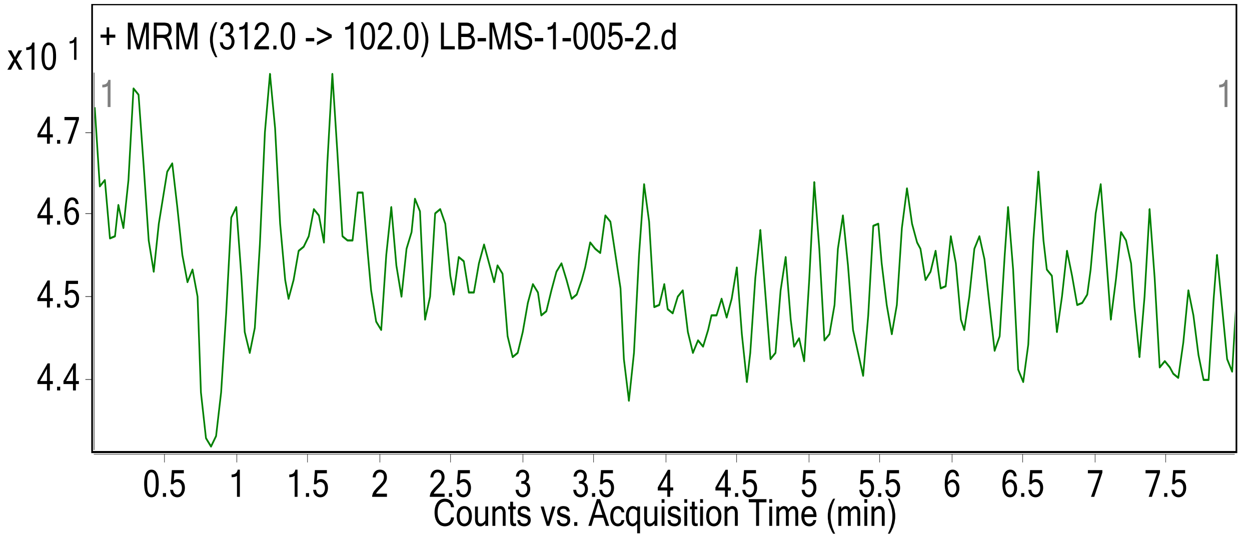

--- End Of Report ---

Qualitative Analysis Report

**Data Filename**	LB-MS-1-005-4.d	**Sample Name**	
**Sample Type**	Sample	**Position**	Vial 5
**Instrument Name**	Instrument 1	**User Name**	
**Acq Method**	AHL_MRM2.m	**Acquired Time**	11/7/2022 2:53:32 PM
**IRM Calibration Status**	Not Applicable	**DA Method**	B&S_trial_02232021.m
**Comment**			

User Chromatograms

Fragmentor Voltage 100 **Collision Energy** 5 **Ionization Mode** ESI

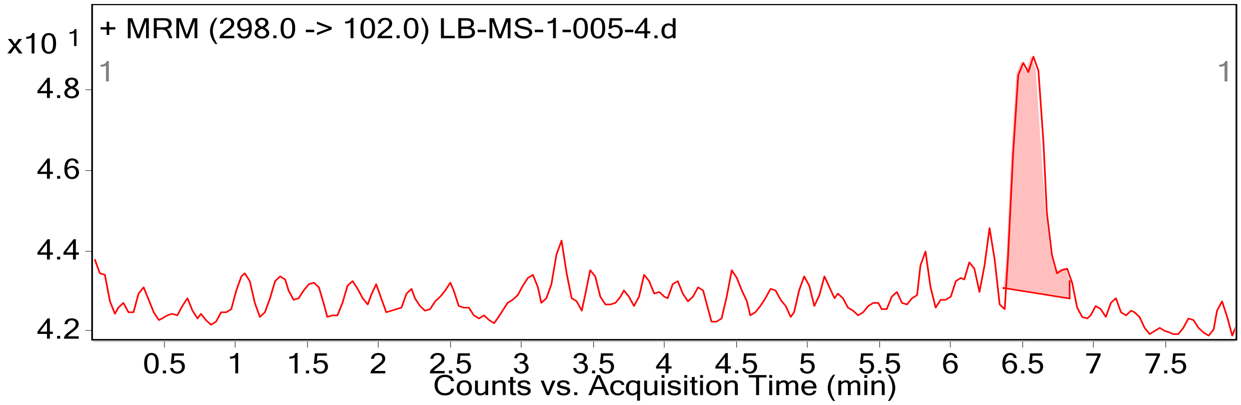

Integration Peak List

Peak	Start	RT	End	Height	Area	Area %
1	6.37	6.56	6.83	6	84	100

Fragmentor Voltage 100 **Collision Energy** 5 **Ionization Mode** ESI

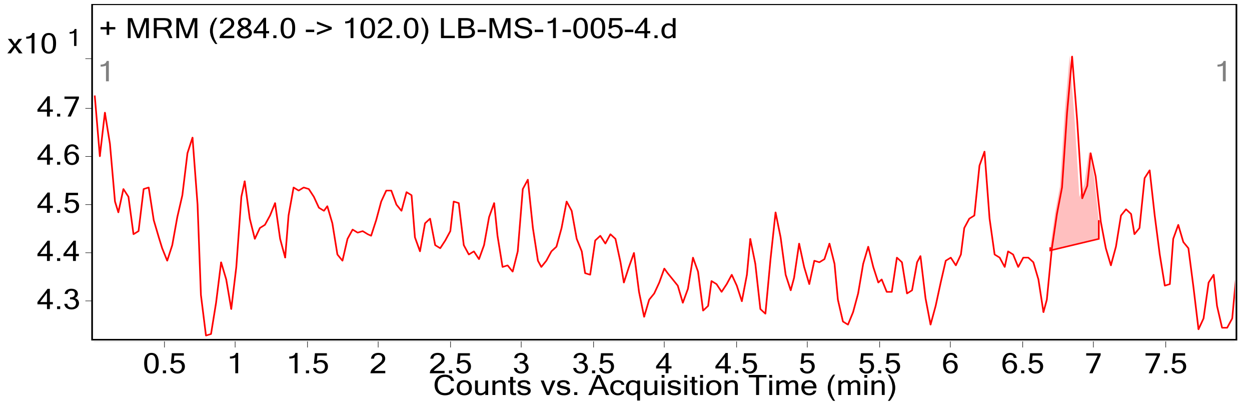

Integration Peak List

Peak	Start	RT	End	Height	Area	Area %
1	6.7	6.83	7.04	4	34	100

Fragmentor Voltage 100 **Collision Energy** 5 **Ionization Mode** ESI

Qualitative Analysis Report

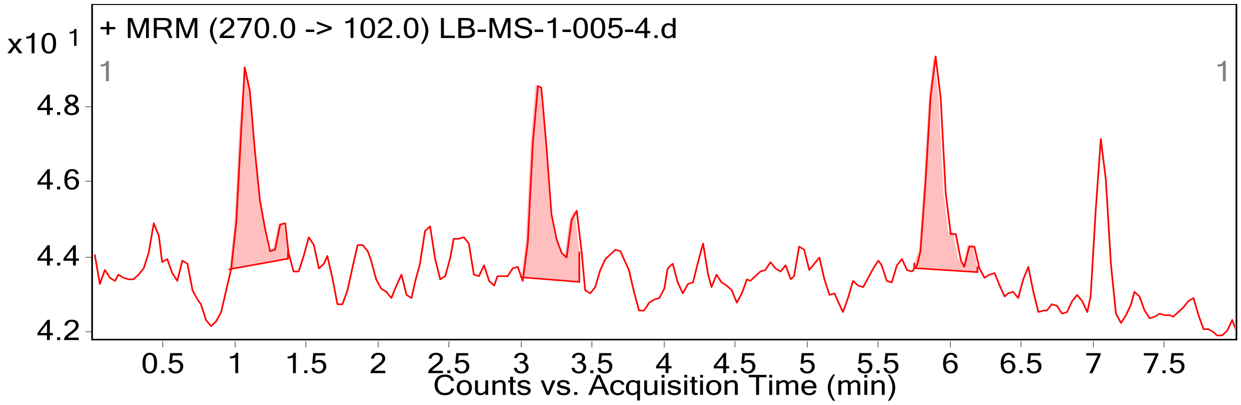

Integration Peak List

Peak	Start	RT	End	Height	Area	Area %
1	0.96	1.07	1.37	5	48	85.73
2	3	3.12	3.41	5	56	100
3	5.75	5.89	6.19	6	49	88.24

Fragmentor Voltage 100 **Collision Energy** 5 **Ionization Mode** ESI

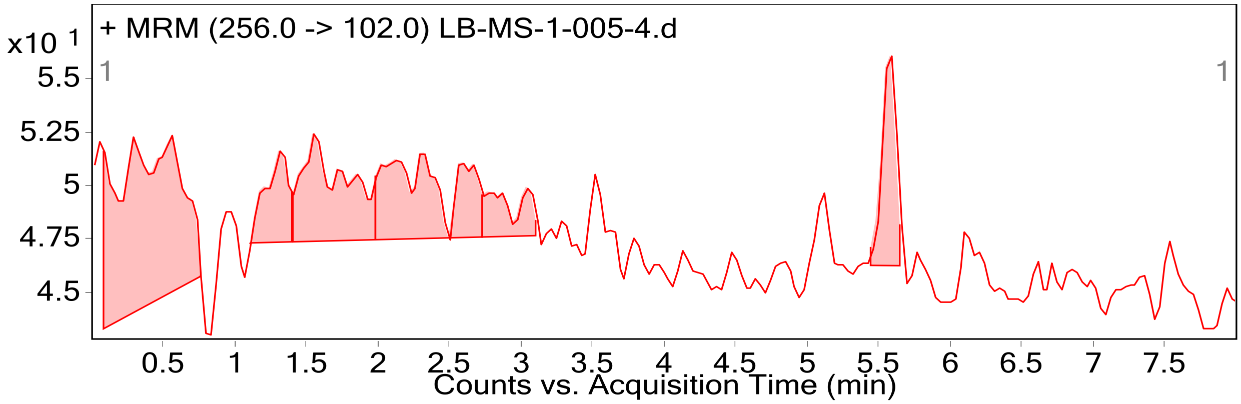

Integration Peak List

Peak	Start	RT	End	Height	Area	Area %
1	0.08	0.55	0.76	7	247	100
2	1.11	1.32	1.4	4	49	19.88
3	1.41	1.55	1.99	5	111	45.07
4	1.99	2.31	2.73	4	131	52.92
5	2.73	3.04	3.11	2	39	15.66
6	5.45	5.57	5.65	10	72	29.12

Fragmentor Voltage 100 **Collision Energy** 5 **Ionization Mode** ESI

Qualitative Analysis Report

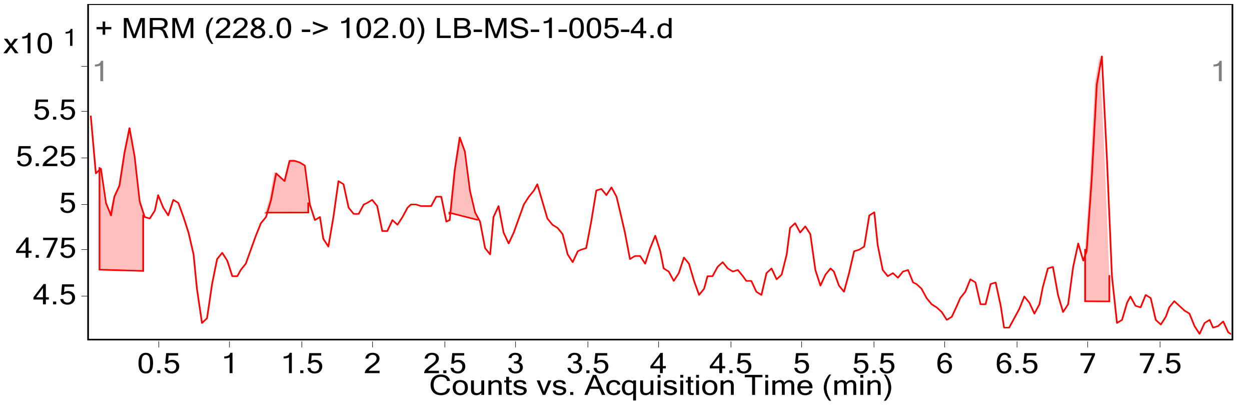

Integration Peak List

Peak	Start	RT	End	Height	Area	Area %
1	0.09	0.29	0.39	8	99	100
2	1.25	1.43	1.55	3	37	37.73
3	2.53	2.61	2.74	4	25	25.59
4	6.98	7.07	7.15	13	90	90.75

Fragmentor Voltage 100 **Collision Energy** 5 **Ionization Mode** ESI

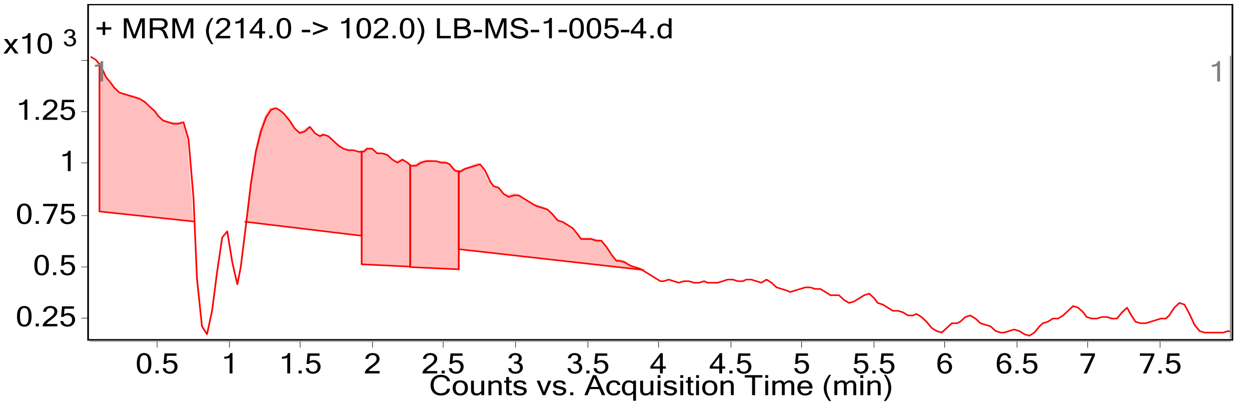

Integration Peak List

Peak	Start	RT	End	Height	Area	Area %
1	0.09	0.65	0.75	482	21612	97.21
2	1.11	1.31	1.92	577	22232	100
3	1.92	1.97	2.26	569	11503	51.74
4	2.26	2.39	2.6	527	10451	47.01
5	2.6	2.73	3.89	433	16717	75.2

Fragmentor Voltage 100 **Collision Energy** 5 **Ionization Mode** ESI

Qualitative Analysis Report

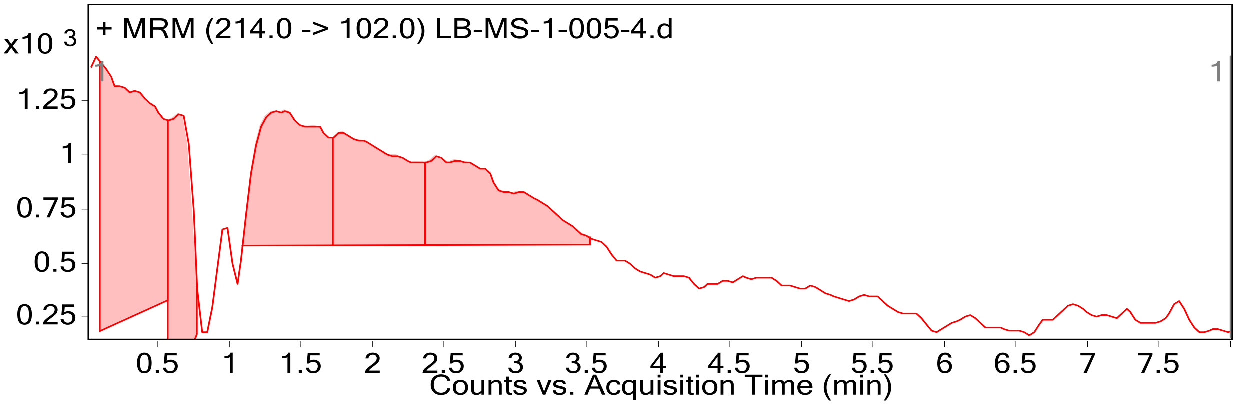

Integration Peak List

Peak	Start	RT	End	Height	Area	Area %
1	0.1	0.33	0.57	1029	30272	100
2	0.57	0.65	0.77	1189	11937	39.43
3	1.09	1.39	1.72	627	19914	65.78
4	1.72	1.78	2.37	527	17380	57.41
5	2.37	2.45	3.52	414	18009	59.49

Fragmentor Voltage 100 **Collision Energy** 5 **Ionization Mode** ESI

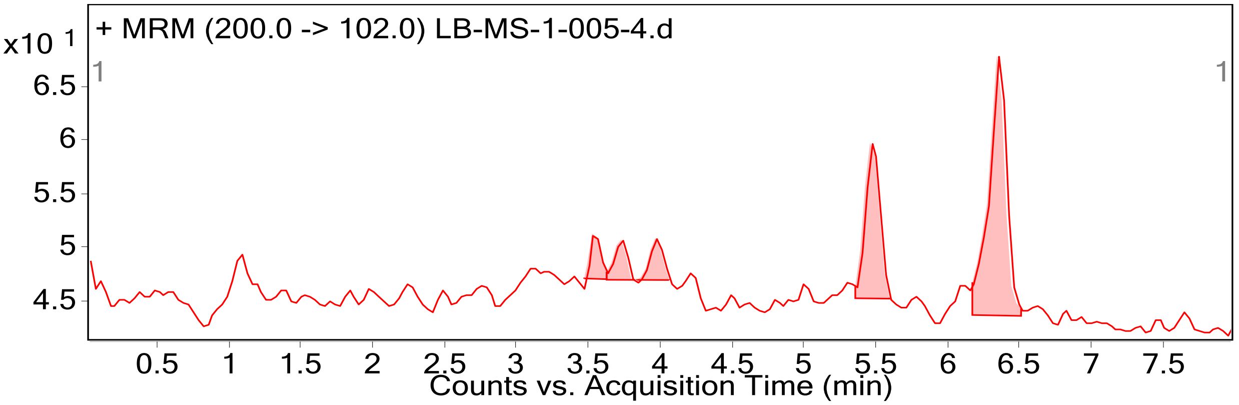

Integration Peak List

Peak	Start	RT	End	Height	Area	Area %
1	3.47	3.54	3.63	4	22	10.51
2	3.63	3.72	3.82	4	24	11.46
3	3.84	3.97	4.06	4	24	11.76
4	5.36	5.47	5.6	14	110	53.21
5	6.17	6.35	6.51	24	207	100

Fragmentor Voltage 100 **Collision Energy** 5 **Ionization Mode** ESI

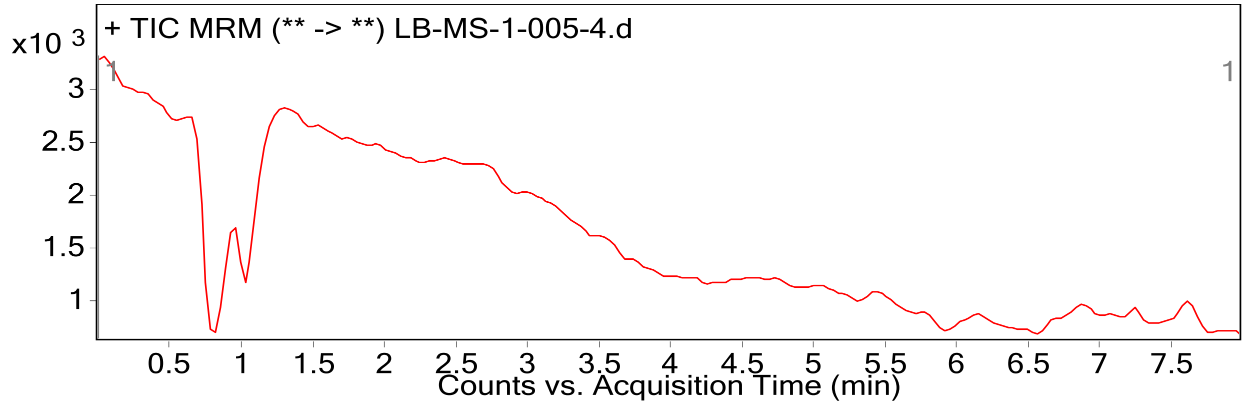

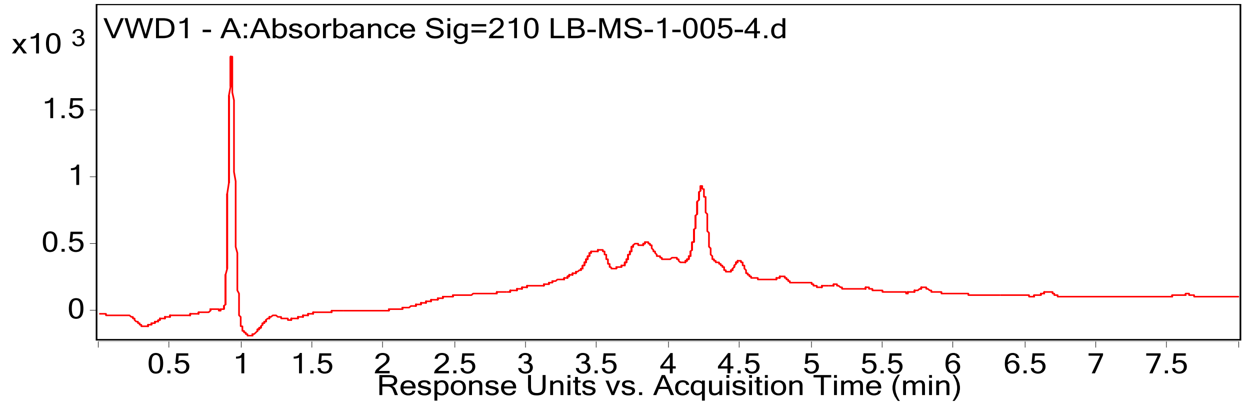

Fragmentor Voltage 100 **Collision Energy** 5 **Ionization Mode** ESI

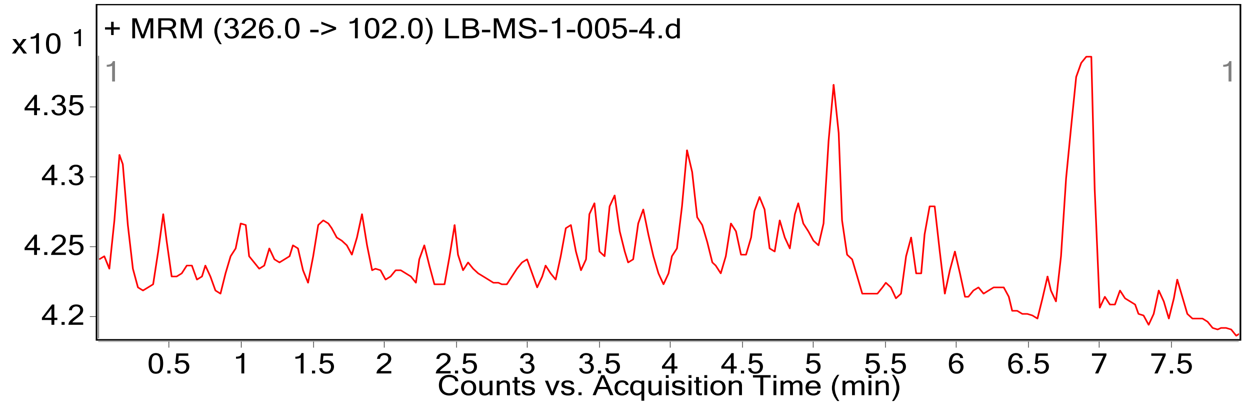

Fragmentor Voltage 100 **Collision Energy** 5 **Ionization Mode** ESI

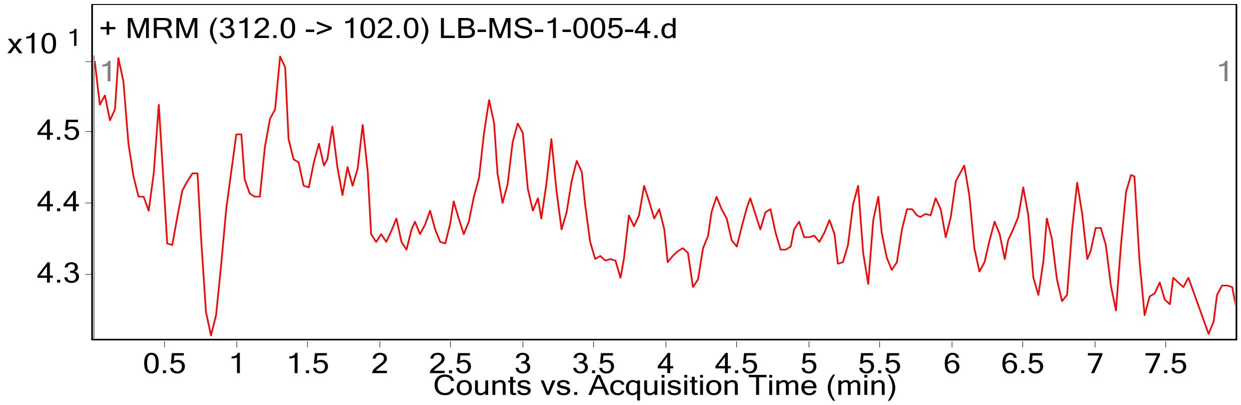

--- End Of Report ---
