# Supplementary material for: N-acyl-homoserine lactone-based quorum sensing beyond canonical lineages: insights from Actinomycetota
Source: Front Microbiol. 2026 Apr 20;17:1738013. doi: 10.3389/fmicb.2026.1738013 (PMC13136126; doi:10.3389/fmicb.2026.1738013)
Supplement: Supplementary file 7 [file Image_1.pdf]

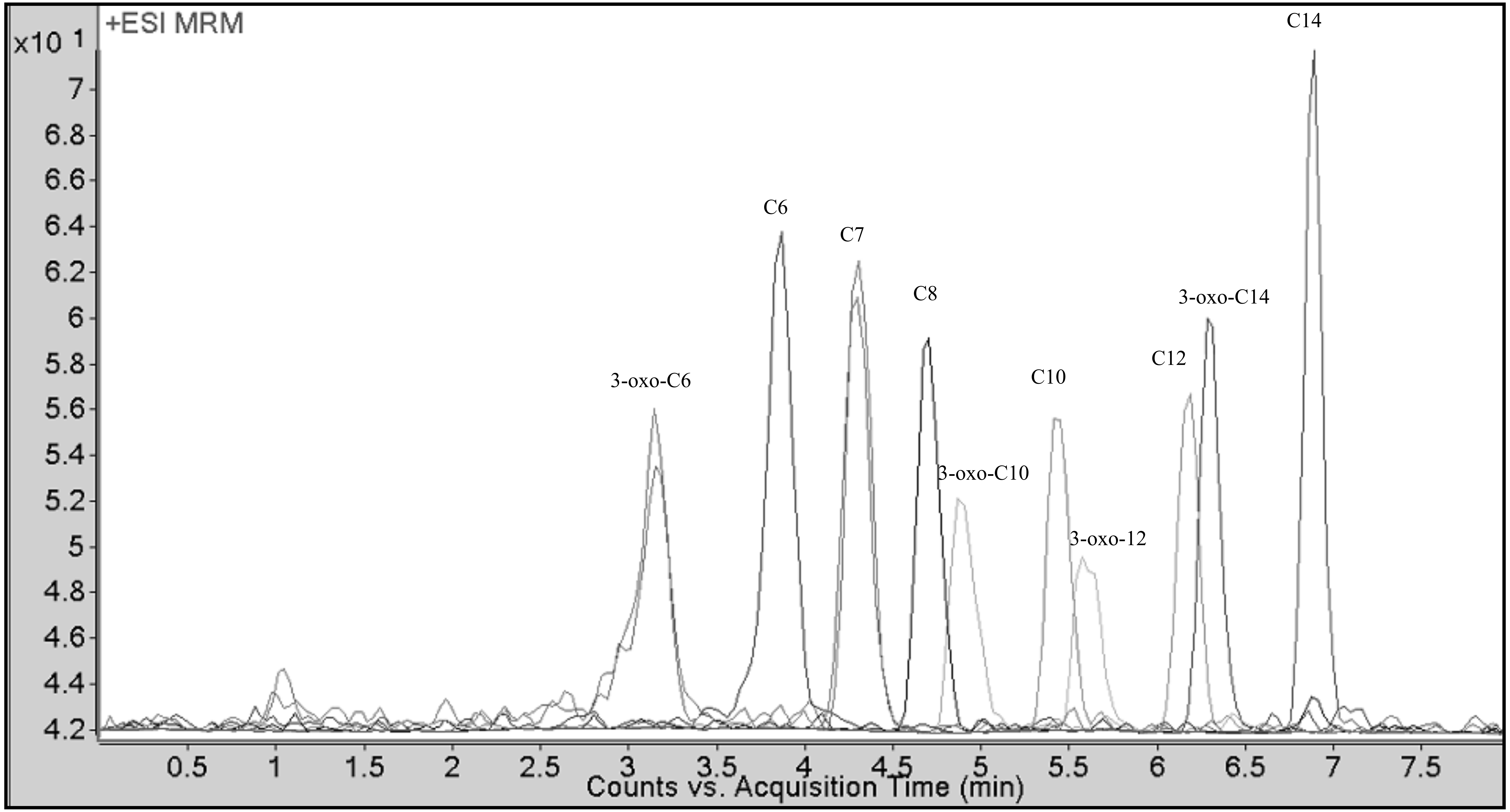

Supplemental Figure 1. Representative extracted ion chromatogram (EIC) of *N*-acyl homoserine lactone (AHL) standards at 12.5 ng/mL. These standards were used for all AHL extractions performed. Standards were prepared prior to each liquid chromatography multiple reaction monitoring mass spectrometry (LC-MRM-MS) run.
