# Supplementary material for: N-acyl-homoserine lactone-based quorum sensing beyond canonical lineages: insights from Actinomycetota
Source: Front Microbiol. 2026 Apr 20;17:1738013. doi: 10.3389/fmicb.2026.1738013 (PMC13136126; doi:10.3389/fmicb.2026.1738013)
Supplement: Supplementary file 8 [file Image_2.pdf]

24h	Environment	
**Replicate**	**GYE**	**GYE + 10% IIa Brine**
**A**	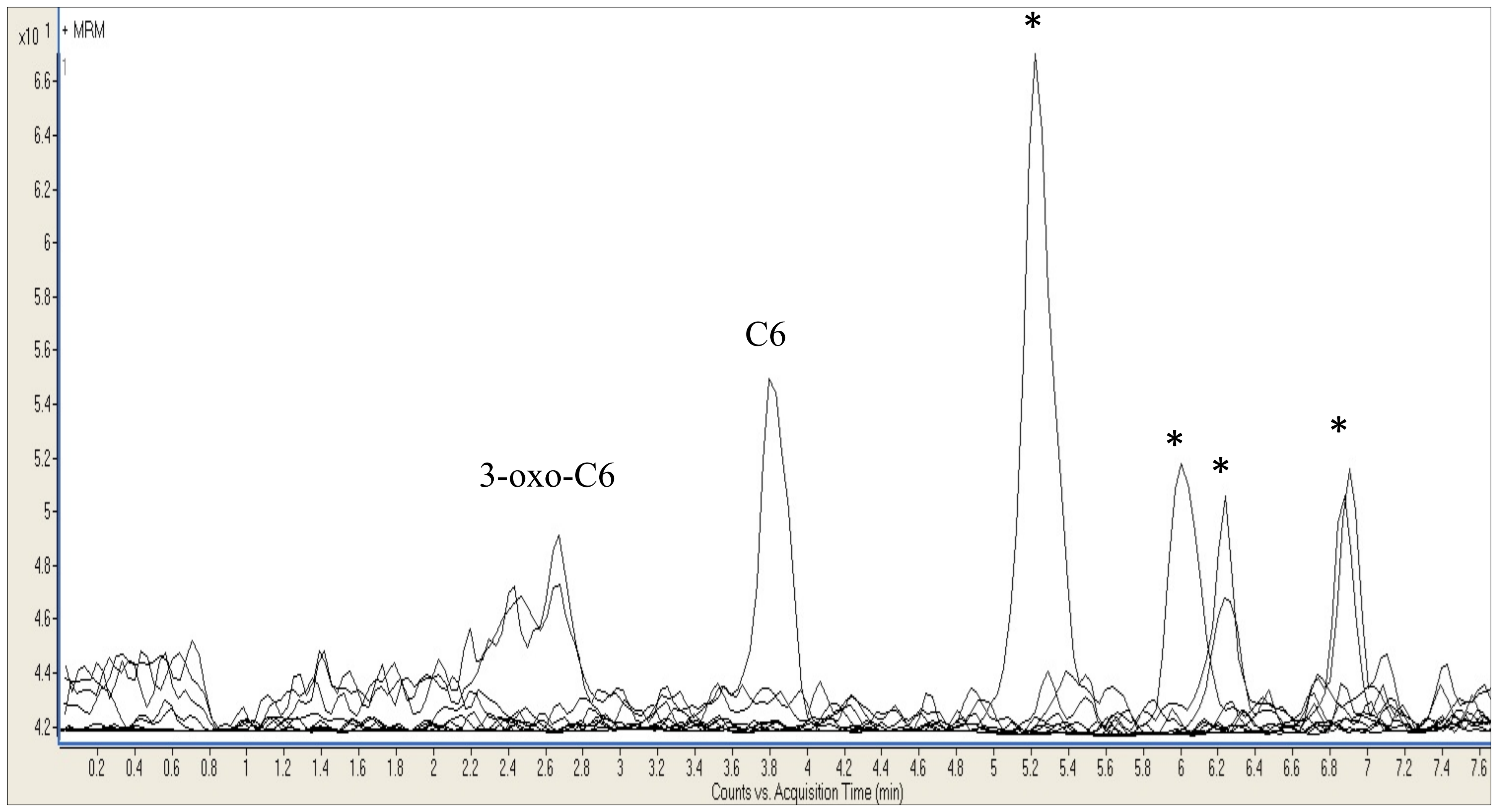	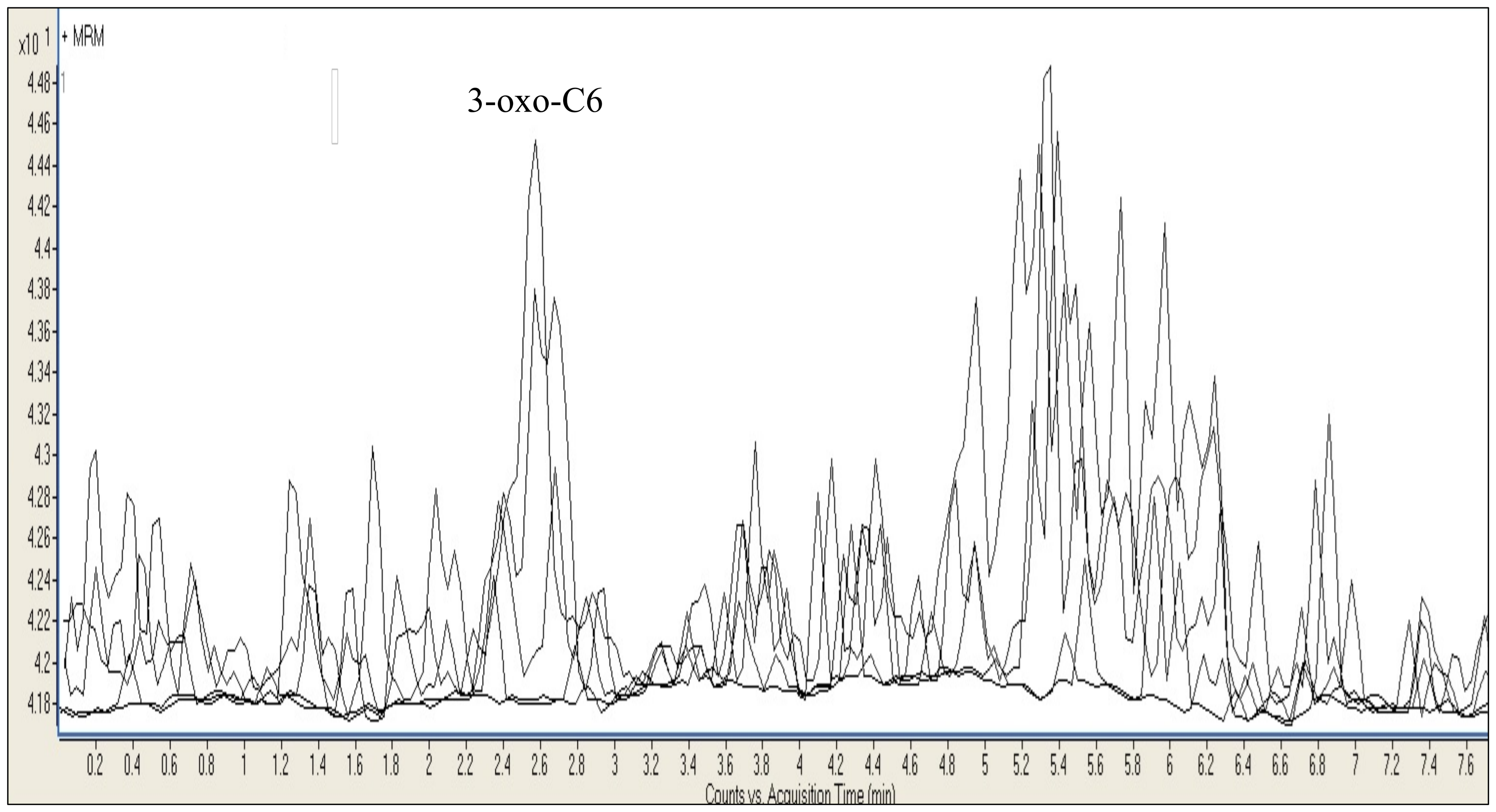
**B**	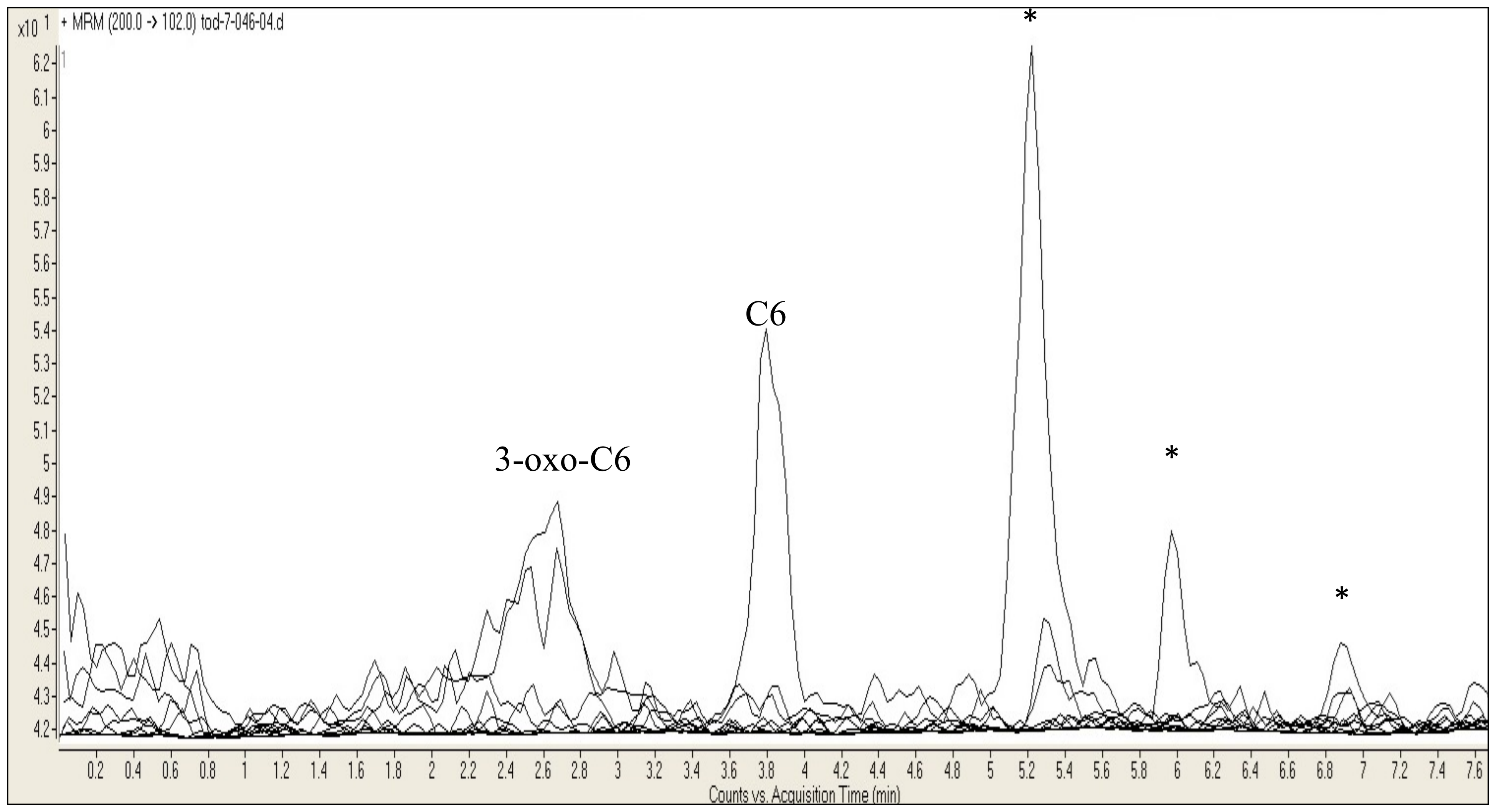	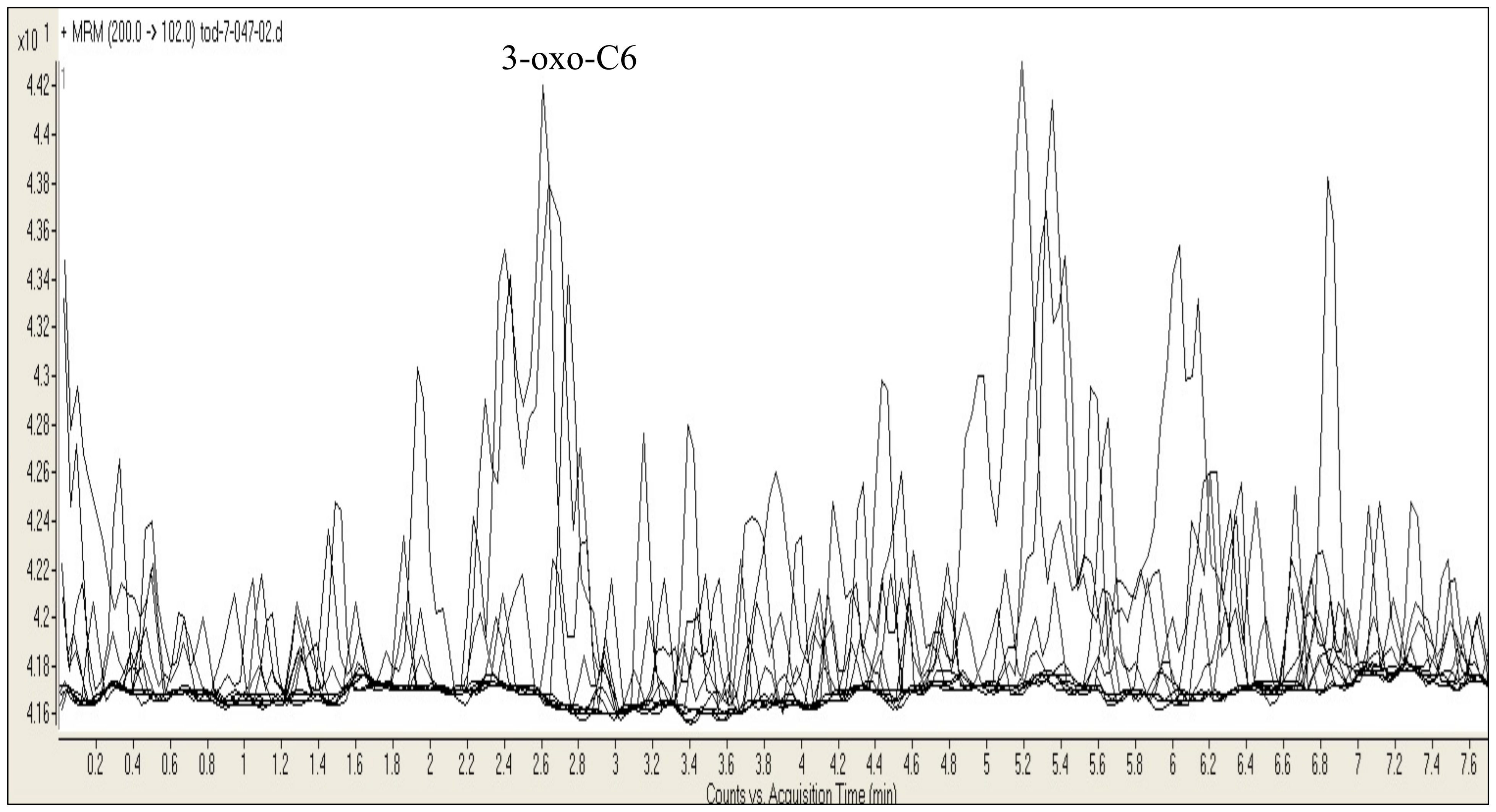
**C**	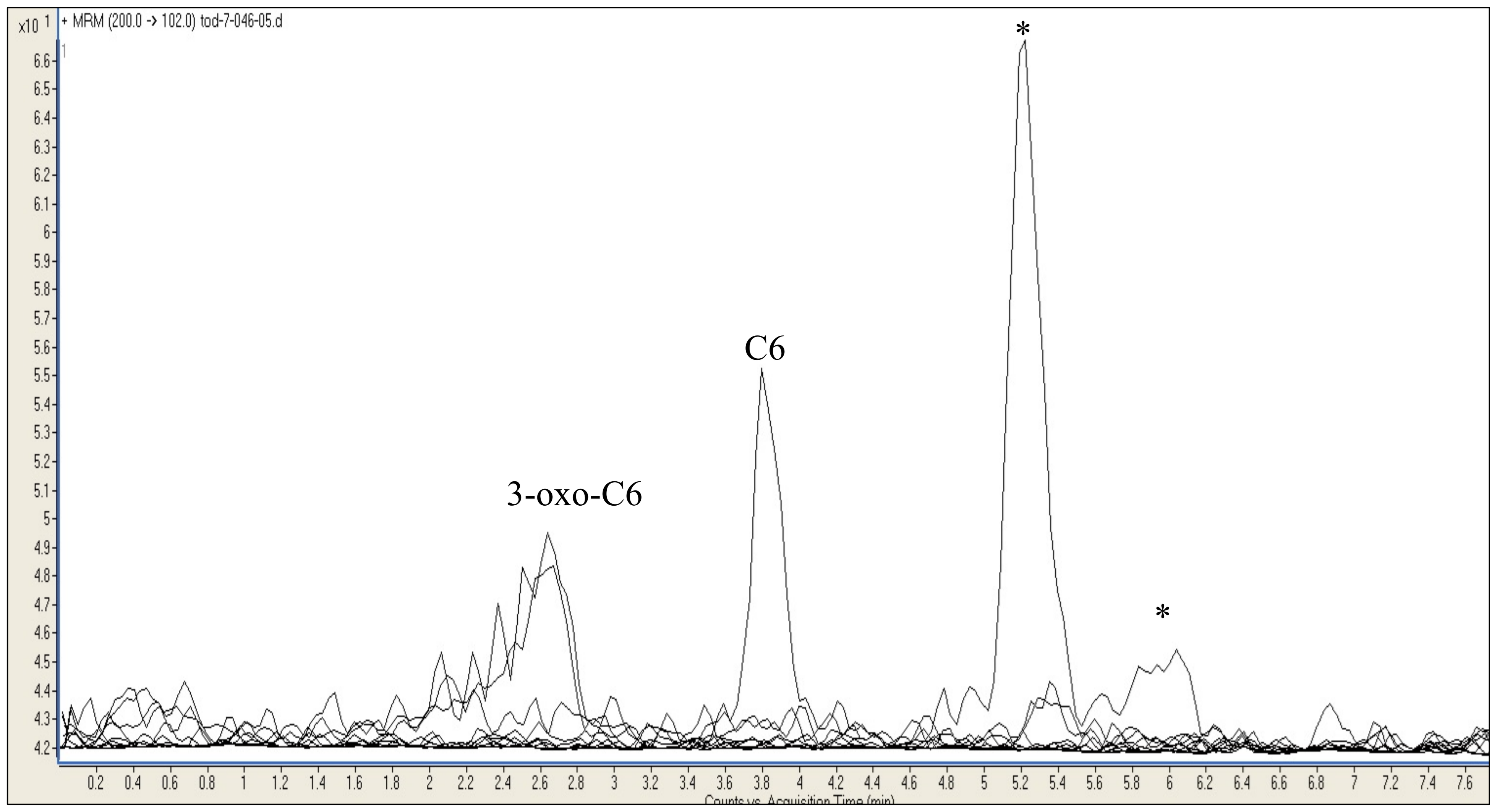	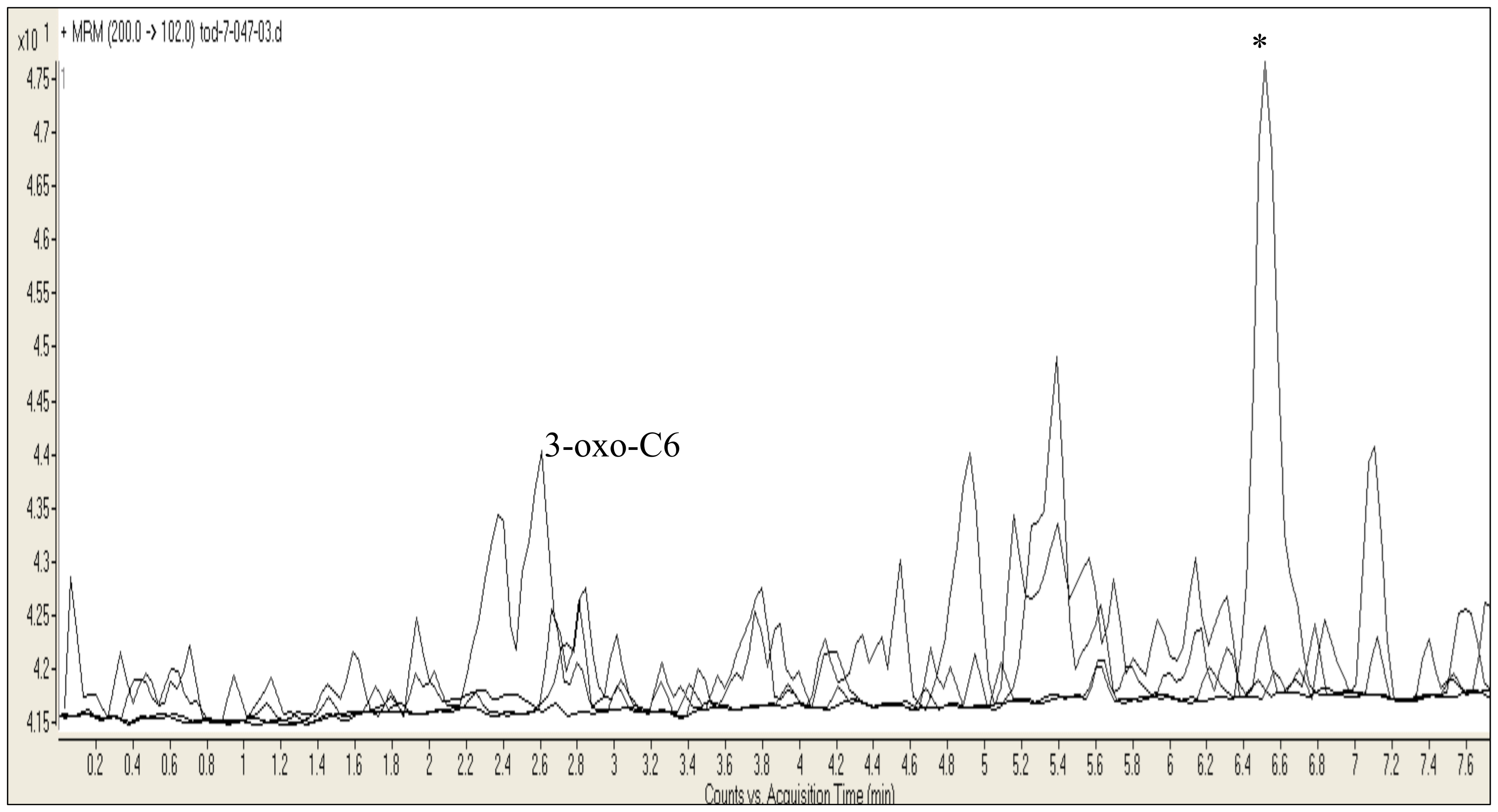

<table>
<tr><th>48h</th><th colspan="2">Environment</th></tr>
<tr><th>Replicate</th><th>GYE</th><th>GYE + 10% IIa Brine</th></tr>
<tr><td>A</td><td>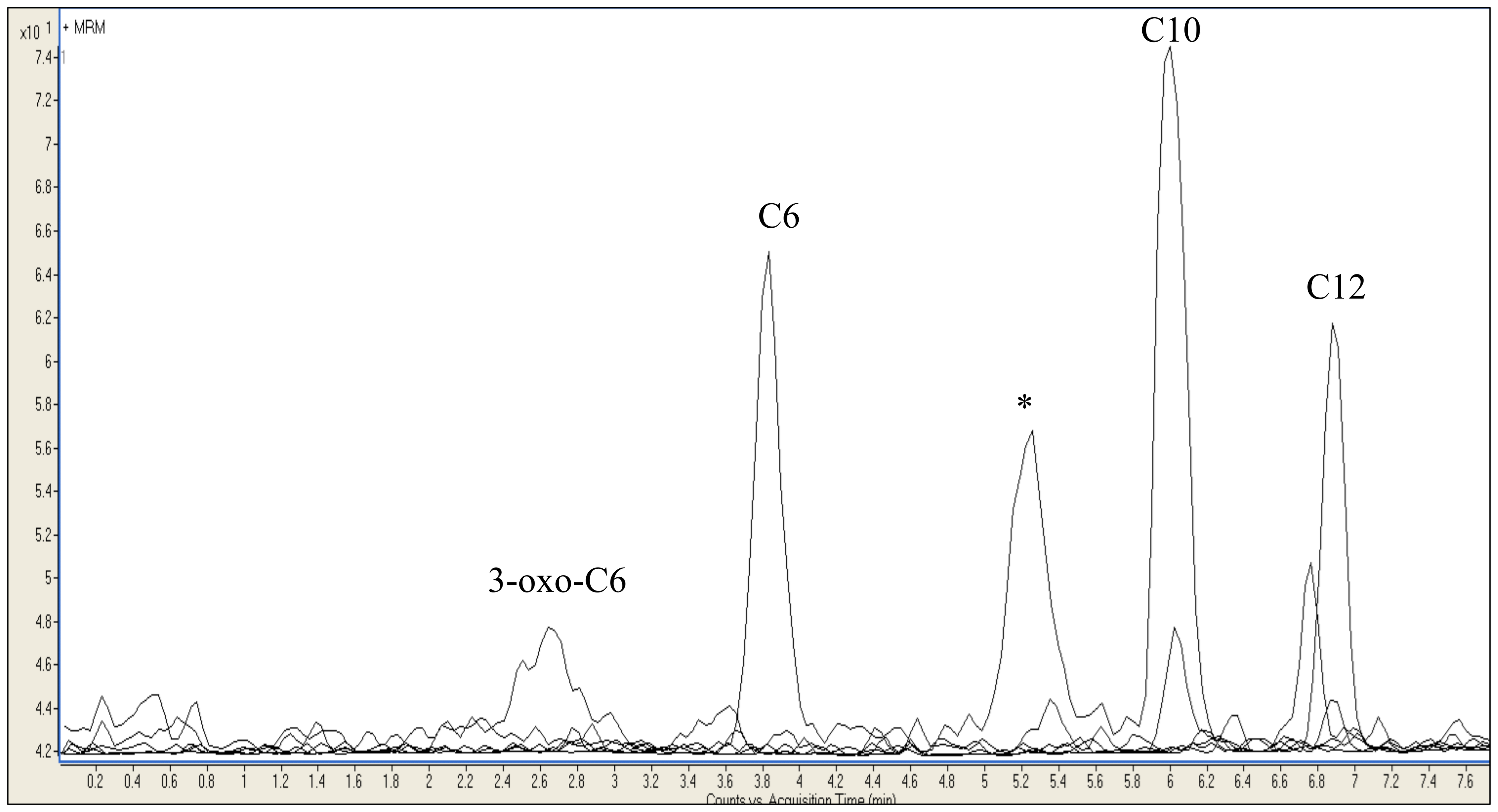
</td><td>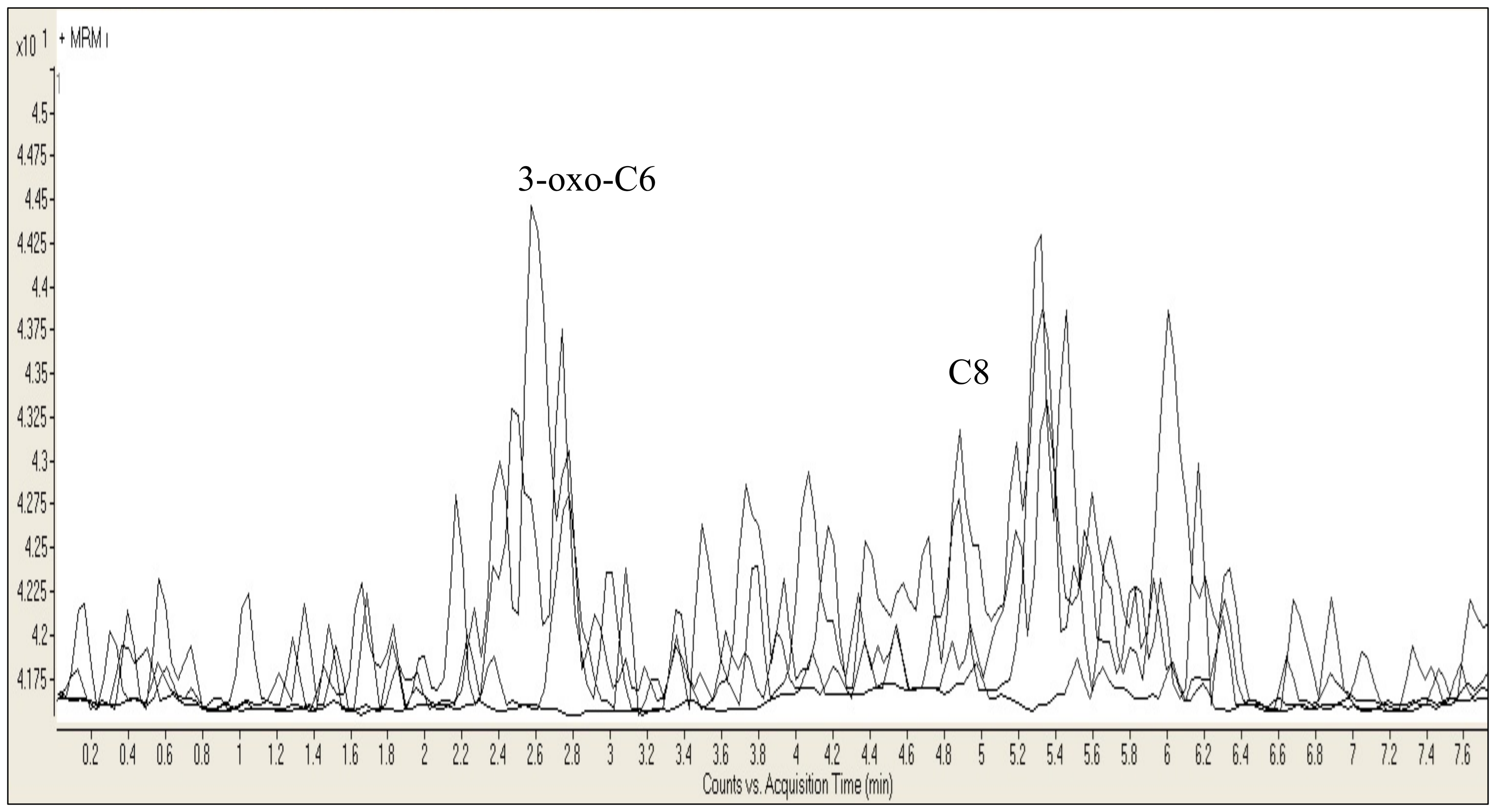
</td></tr>
<tr><td>B</td><td>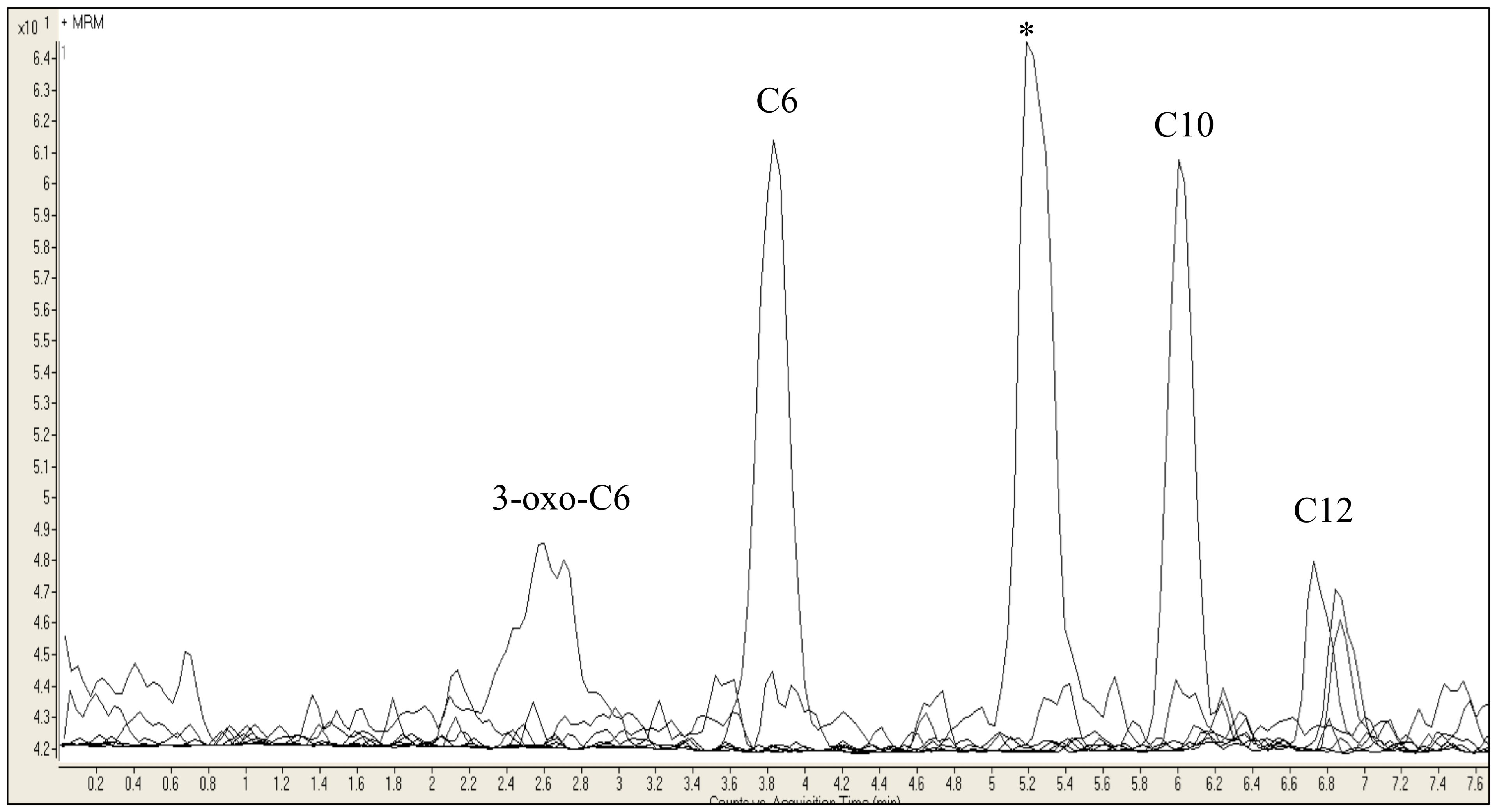
</td><td>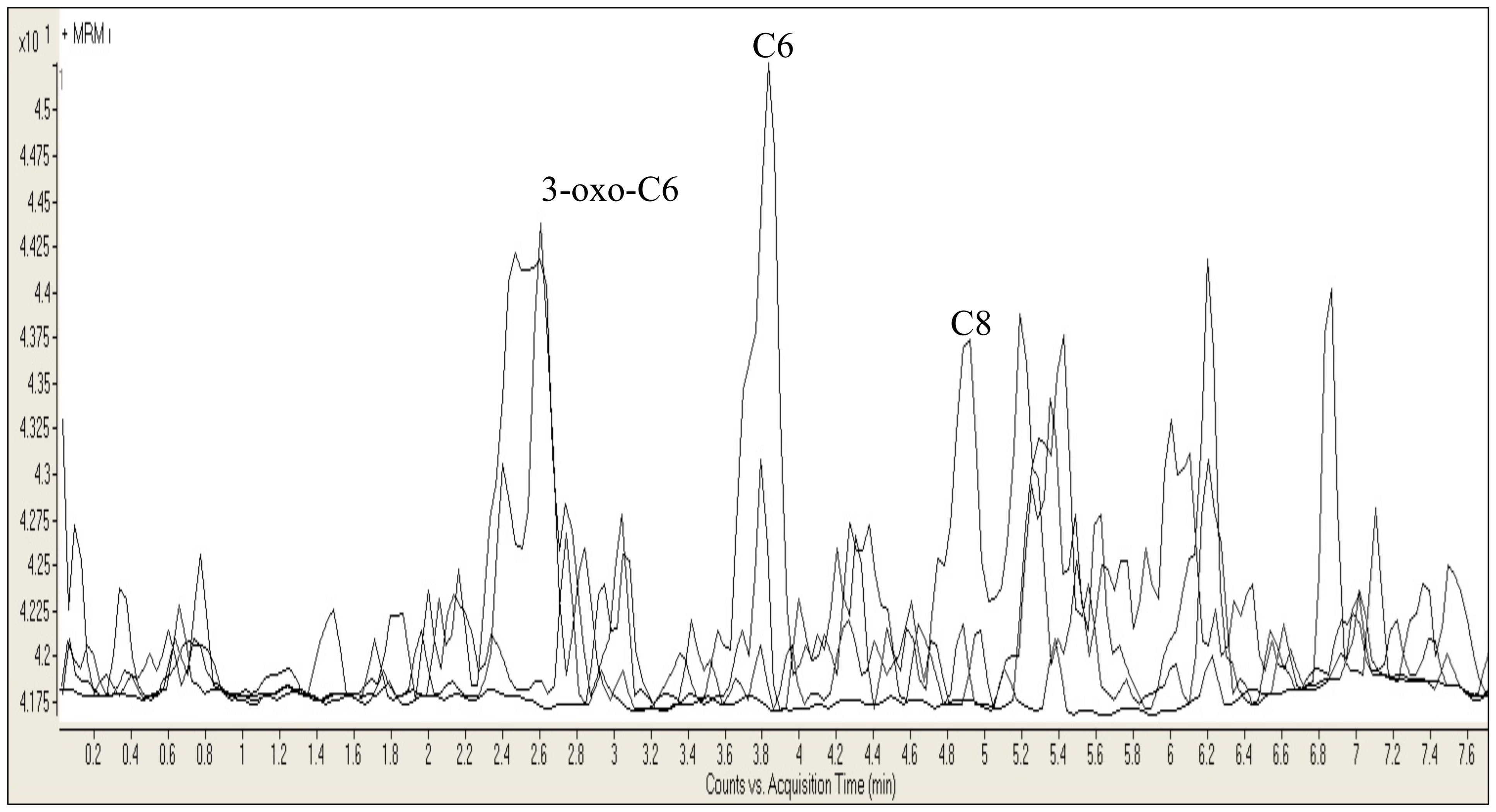
</td></tr>
<tr><td>C</td><td>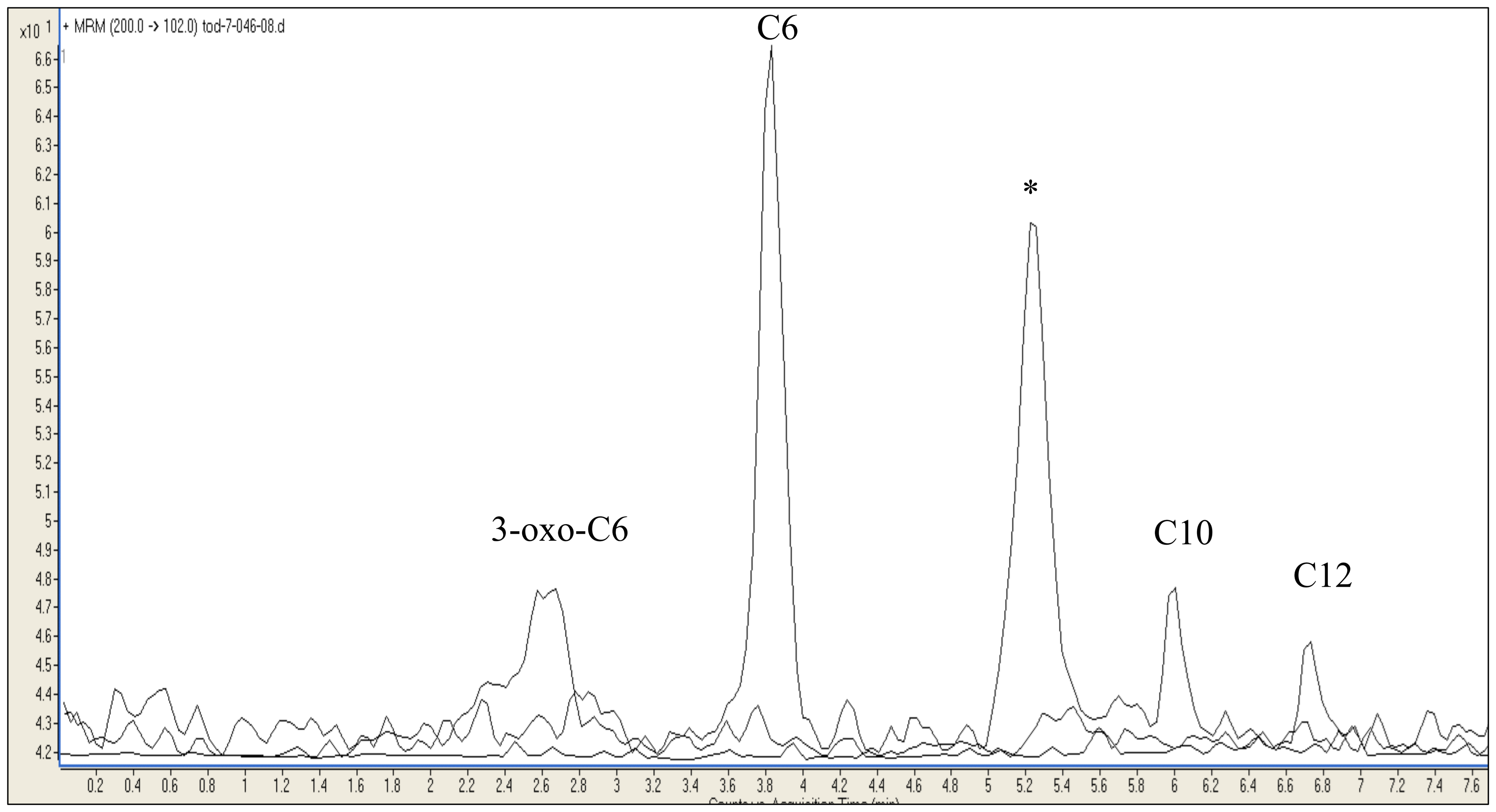
</td><td>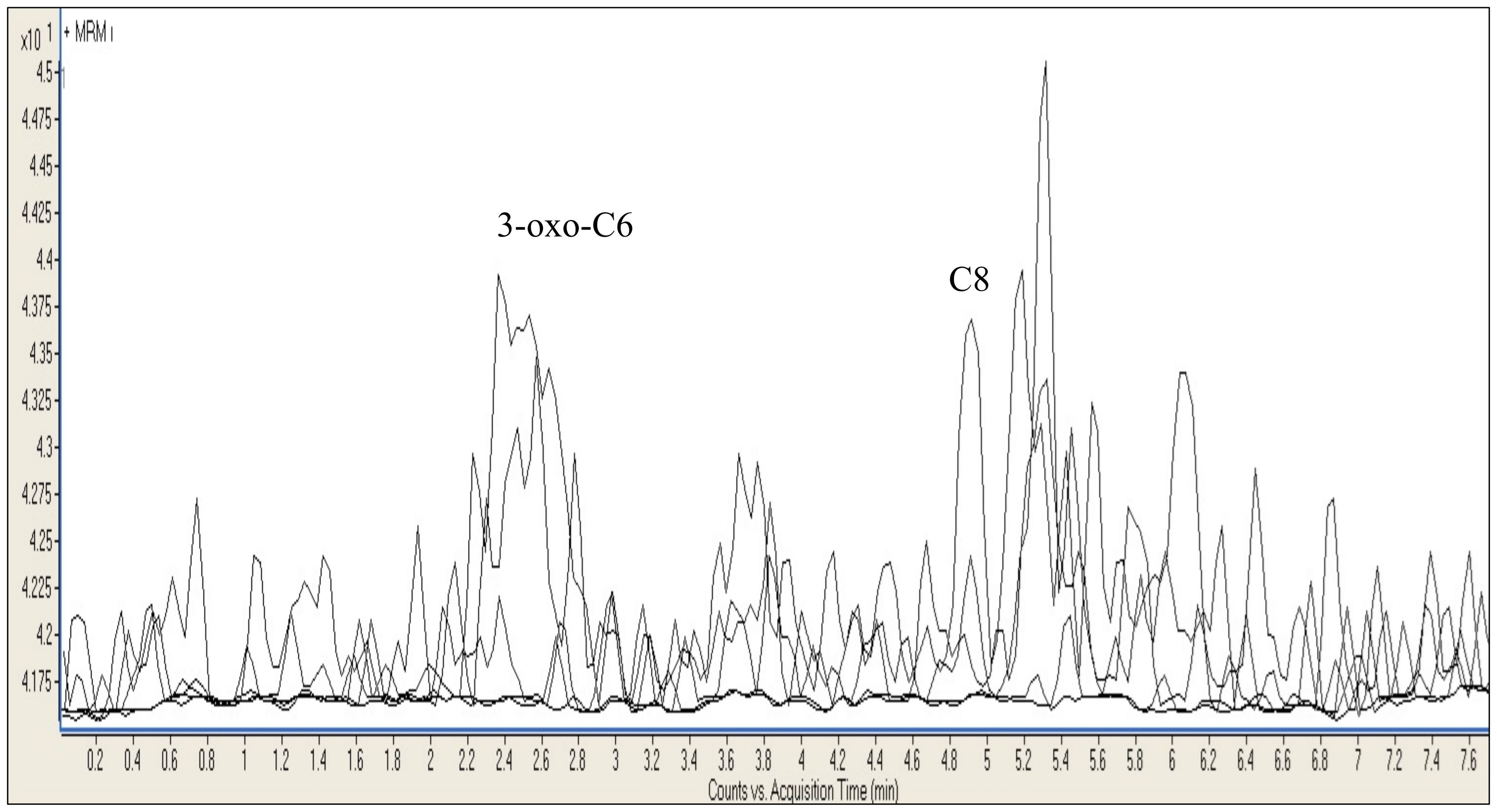
</td></tr>
</table>

<table>
<tr><th>72h</th><th colspan="2">Environment</th></tr>
<tr><th>Replicate</th><th>GYE</th><th>GYE + 10% IIa Brine</th></tr>
<tr><td>A</td><td>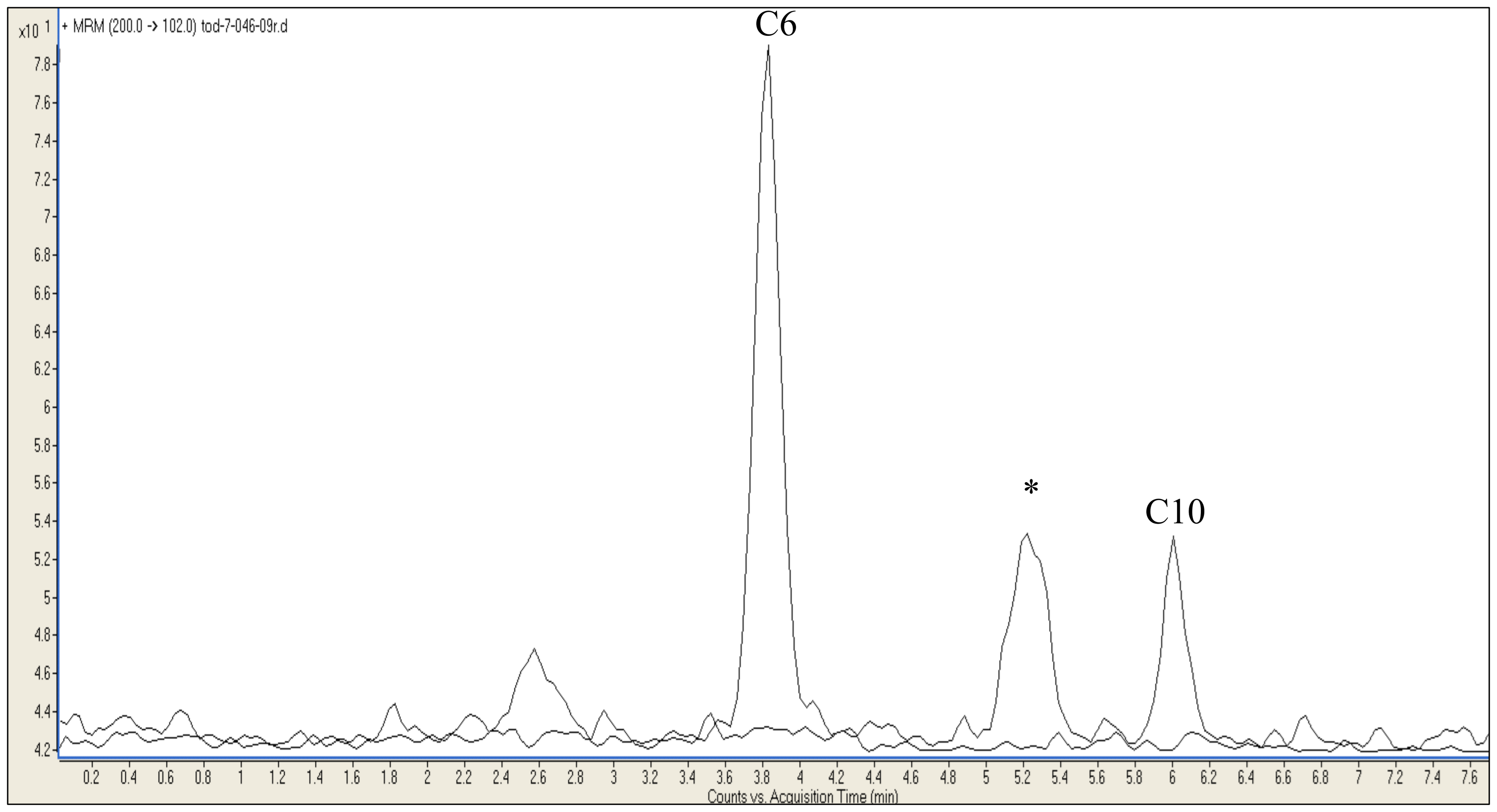
</td><td>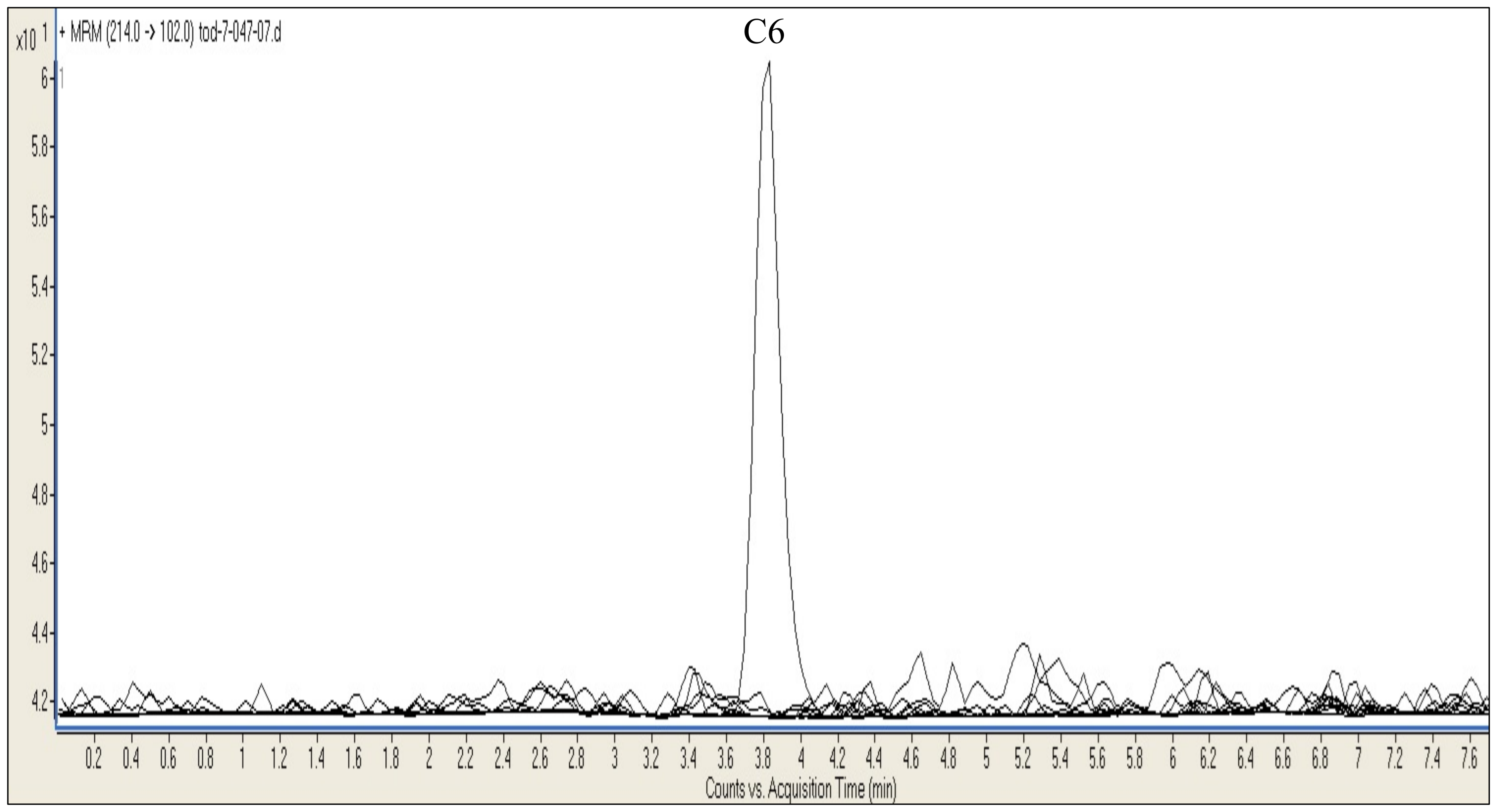
</td></tr>
<tr><td>B</td><td>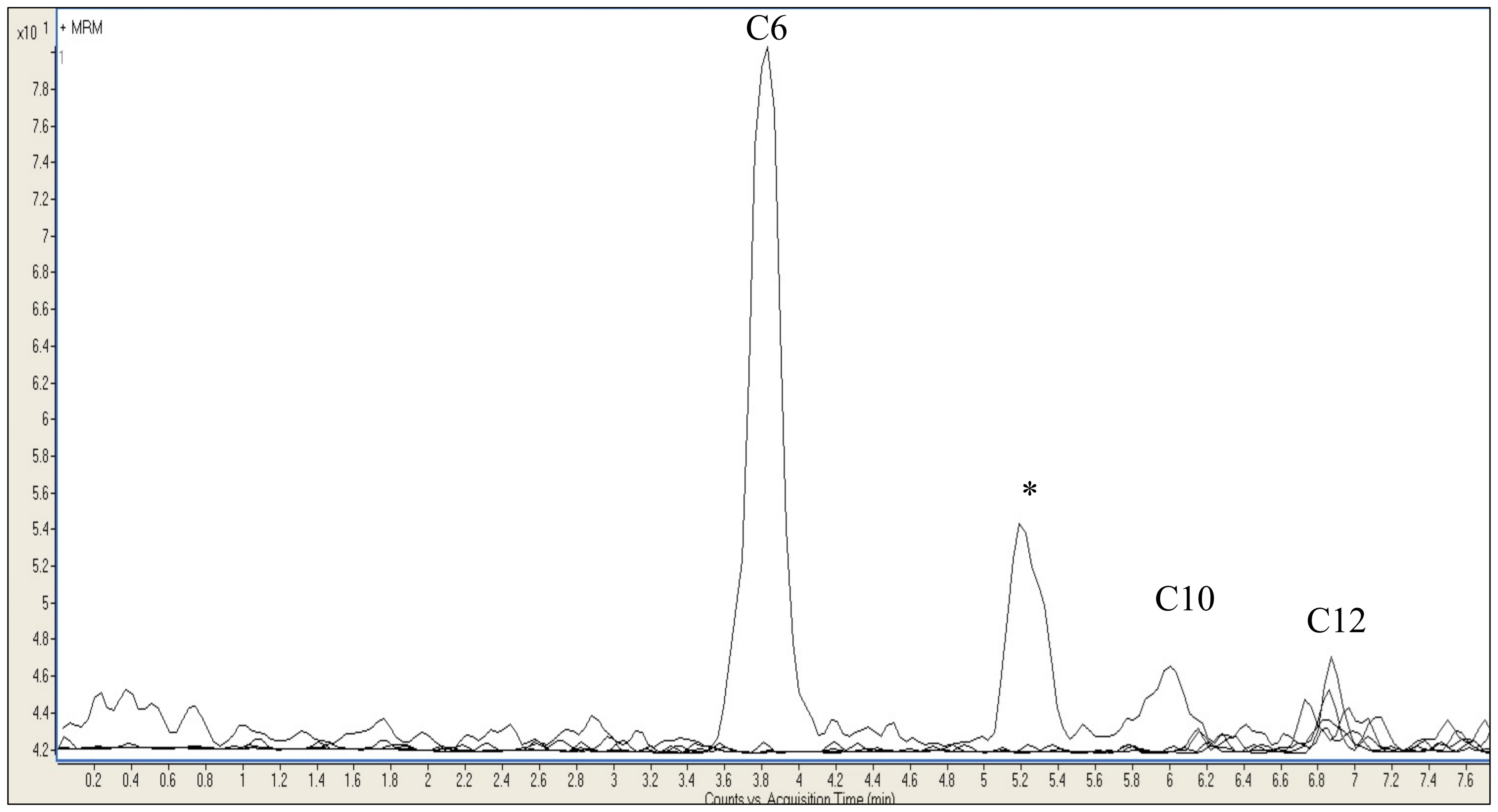
</td><td>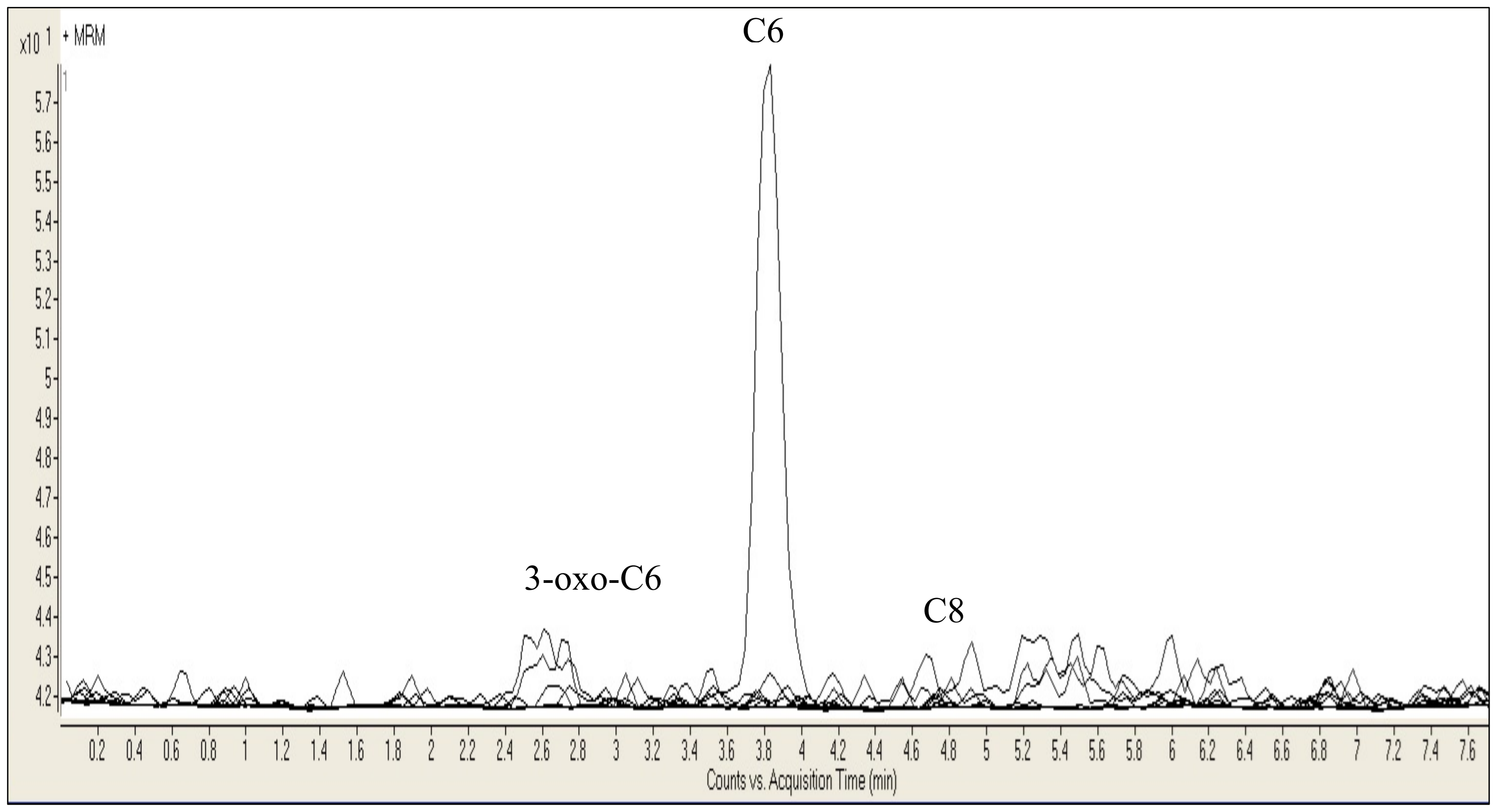
</td></tr>
<tr><td>C</td><td>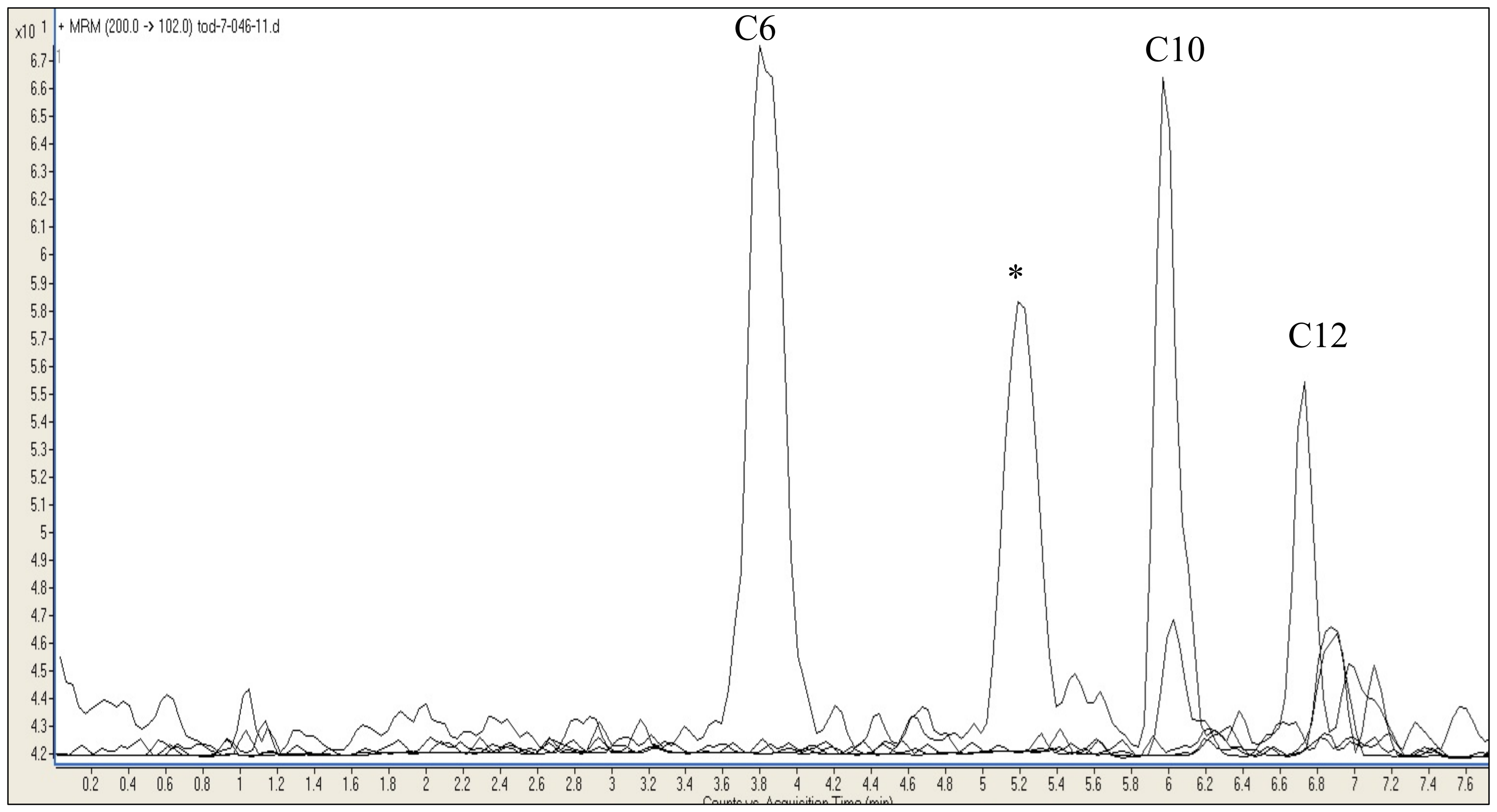
</td><td>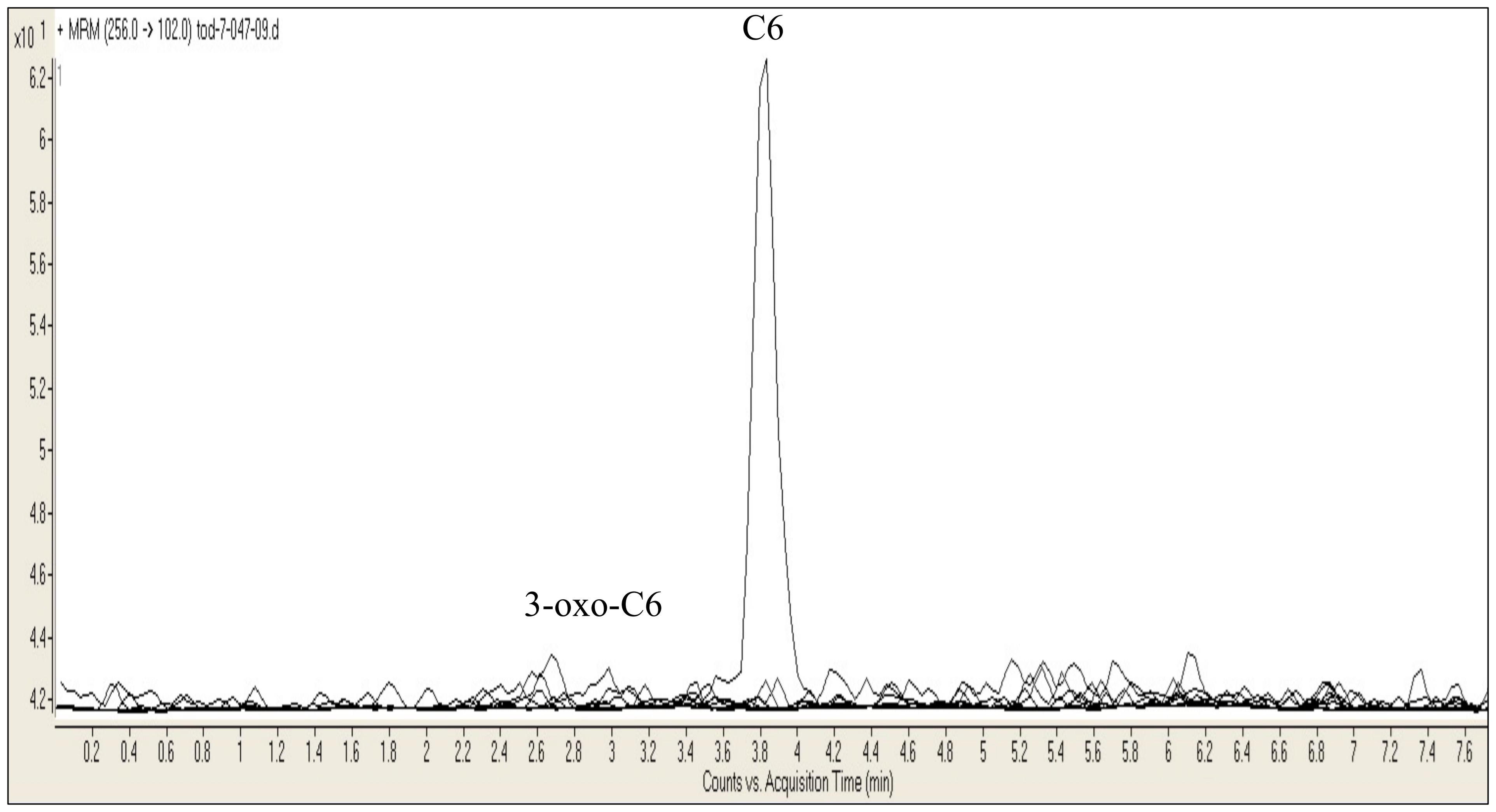
</td></tr>
</table>

Supplemental Figure 2. Representative extracted ion chromatogram (EIC) of *N*-acyl homoserine lactone (AHL) standards used during *Brenneria uluponensis* K61^T extraction analyses at 12.5 ng/mL, and chromatograms representing AHL production of K61^T in Glucose-Yeast Extract (GYE) broth vs. GYE broth supplemented with 10% IIa brine at 24, 48, and 74 hours. The representative extracted ion chromatogram of AHL standards used during K61^T extraction analyses at 12.5 ng/mL is the first trace. Biological replicates are identified as A, B, and C. Time points (24, 48, and 72h) are indicated in the top left corner. (Abbreviations: *=unidentified putative AHL with fragment at *m*/*z* = 102, h=hours).
