# Supplementary figures and images for: N-acyl-homoserine lactone-based quorum sensing beyond canonical lineages: insights from Actinomycetota
Source: Front Microbiol. 2026 Apr 20;17:1738013. doi: 10.3389/fmicb.2026.1738013 (PMC13136126; doi:10.3389/fmicb.2026.1738013)

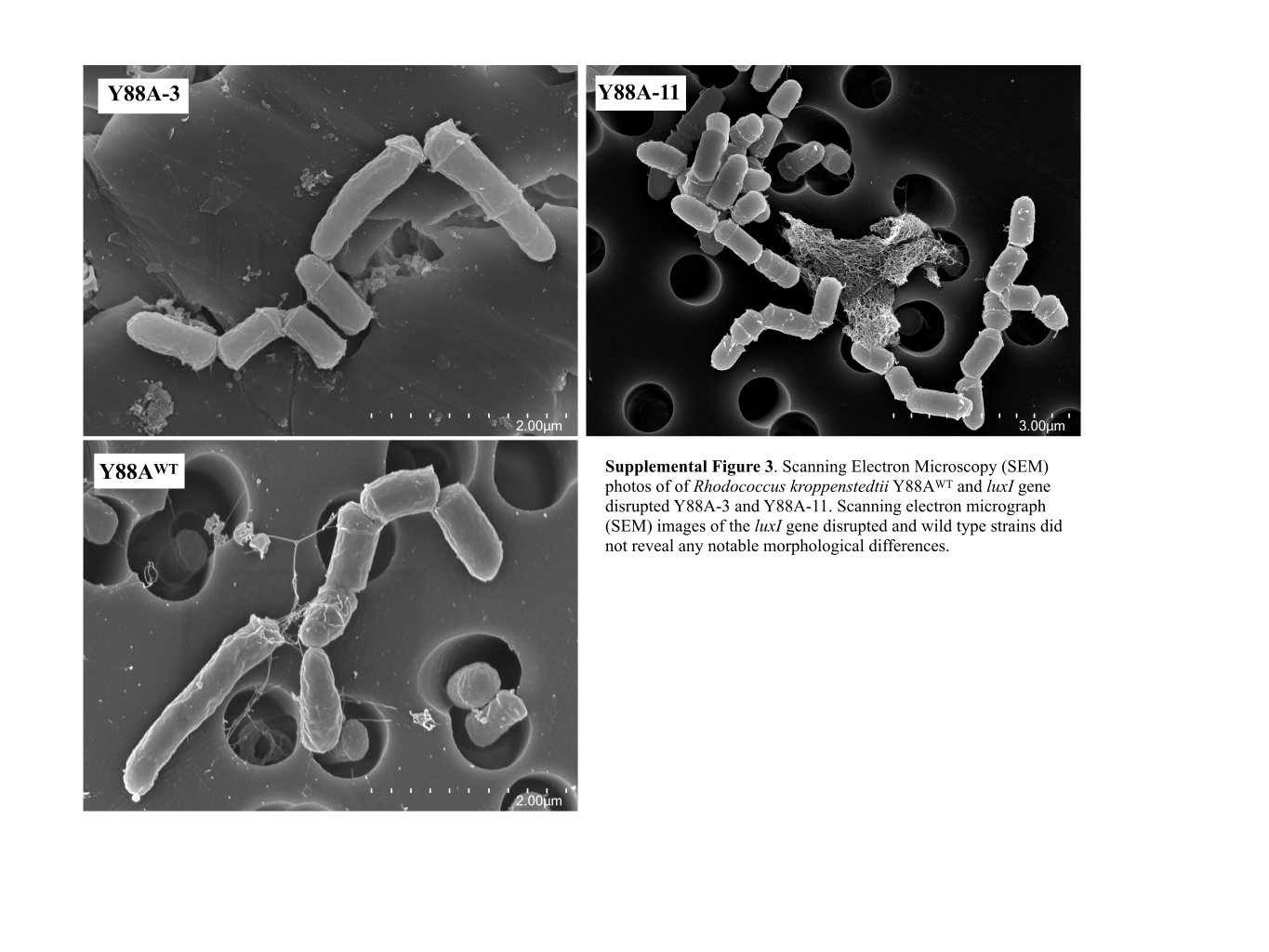

Supplement: Supplementary file 9 [file Image_3.jpeg]
