# Supplementary material for: N-acyl-homoserine lactone-based quorum sensing beyond canonical lineages: insights from Actinomycetota
Source: Front Microbiol. 2026 Apr 20;17:1738013. doi: 10.3389/fmicb.2026.1738013 (PMC13136126; doi:10.3389/fmicb.2026.1738013)
Supplement: Supplementary file 10 [file Image_4.pdf]

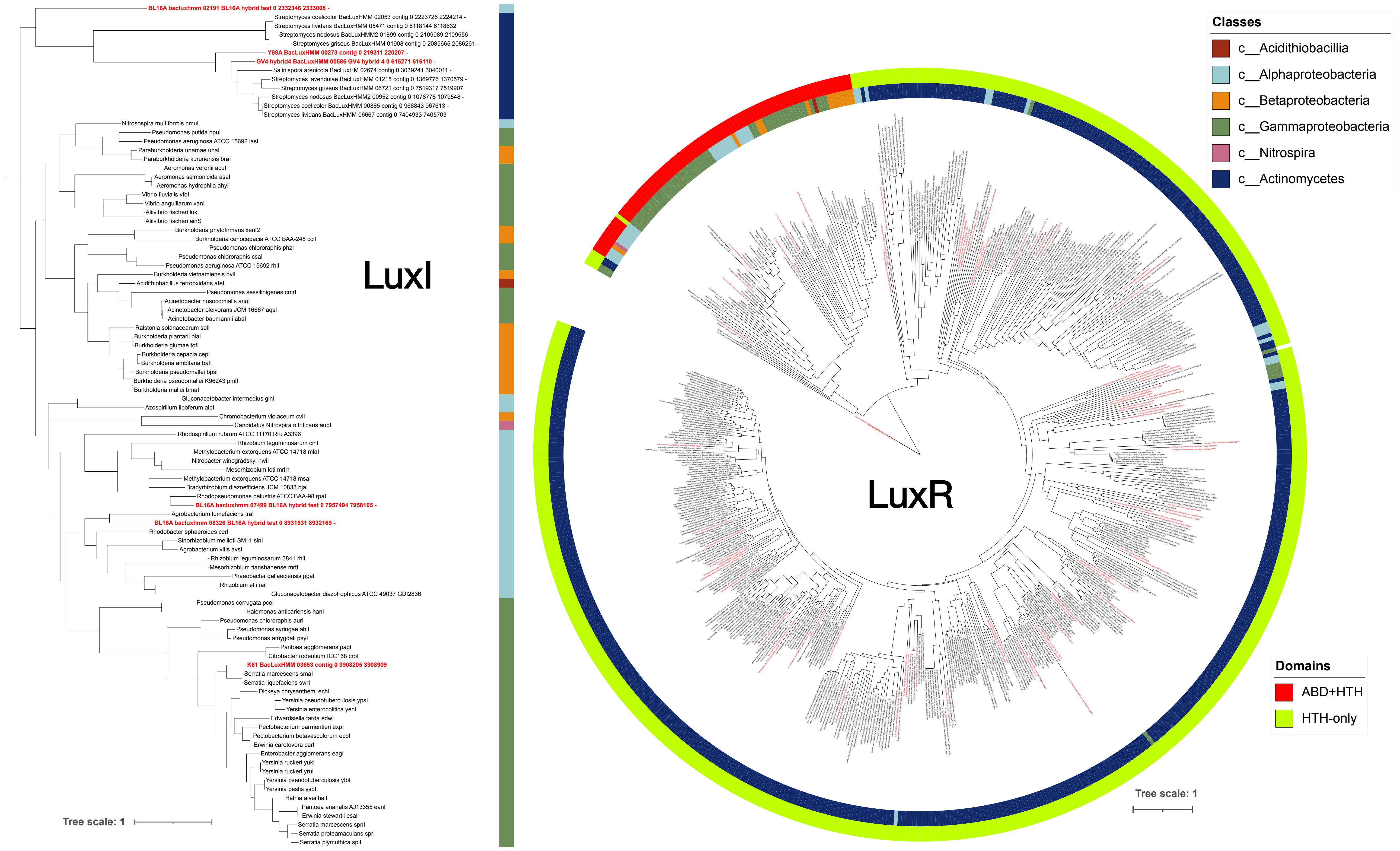

Supplemental Figure 4. Maximum-likelihood phylogenetic trees of LuxI and LuxR homologs identified in this study and from organisms with experimentally verified *luxI/R* QS systems. Amino-acid sequences were aligned using MAFFT (L-INS-i) and trimmed with TrimAl (gap threshold 0.5). The best-fit substitution models (-m LG+F+I+G4 for LuxI or Q.pfam+F+G4 for LuxR; -alrt 1000 -bb 1000) were selected by BIC in IQ-TREE and used to construct trees with 1000 SH-aLRT and ultrafast bootstrap replicates. Trees were visualized in iTOL. Sequences from *Brenneria uluponensis* K61^{T}, *Bradyrhizobium prioriisuperbiae* BL16A^{T}, *Pseudonocardia alni* GV4, and *Rhodococcus kroppenstedtii* Y88A are highlighted in red. Classes represented include Acidithiobacillia, Alphaproteobacteria, Betaproteobacteria, Gammaproteobacteria, Nitrospira, and Actinomycetes, along with LuxI/R homologs from Gram-positive *Salinispora* and *Streptomyces* spp. known to participate in QS. LuxR protein architecture (HTH-only vs. ABD+HTH) is represented by the outer ring. LuxI/R homologs from the Gram-negative Proteobacteria generally clustered with canonical AHL systems, whereas those from Actinobacteria grouped within distinct clades. Notably, one BL16A LuxI homolog (BL16A_bacluxhmm_02191) clustered with Actinobacteria, and several α- and γ-proteobacterial LuxR sequences were interspersed among Gram-positive clades.
