# Supplementary material for: N-acyl-homoserine lactone-based quorum sensing beyond canonical lineages: insights from Actinomycetota
Source: Front Microbiol. 2026 Apr 20;17:1738013. doi: 10.3389/fmicb.2026.1738013 (PMC13136126; doi:10.3389/fmicb.2026.1738013)
Supplement: Supplementary file 14 [file Table_4.docx]

**Supplemental Table 4**. AHL standards used to identify known AHLs or isomers of known AHLs, the *m/z* of their associated protiated ion, and approximate retention time (t_R_).

| **Compound** | **Compound Name** | **Molecular Formula** | **[M+H]^+^** | **~ t_R_(min)** | **InChIKey** |
| --- | --- | --- | --- | --- | --- |
| 3-oxo-C6-HSL | *N*-(3-Oxohexanoyl)-L-  homoserine lactone | C_10_H_15_NO_4_ | 214 | 3.0 | YRYOXRMDHALAFL-QMMMGPOBSA-N |
| C6-HSL | *N*-Hexanoyl-L-  homoserine lactone | C_10_H_17_NO_3_ | 200 | 3.8 | ZJFKKPDLNLCPNP-QMMMGPOBSA-N |
| C7-HSL | *N*-Heptanoyl)-L-  homoserine lactone | C_11_H_19_NO_3_ | 214 | 4.3 | FTMZLSDESAOPSZ-VIFPVBQESA-N |
| C8-HSL | *N*-Octanoyl-L-  homoserine lactone | C_12_H_21_NO_3_ | 228 | 4.6 | JKEJEOJPJVRHMQ-JTQLQIEISA-N |
| 3-oxo-C10-HSL | *N*-(3-Oxodecanoyl)-L-  homoserine lactone | C_14_H_23_NO_4_ | 270 | 4.9 | KYGIKEQVUKTKRR-LBPRGKRZSA-N |
| C10-HSL | *N*-Decanoyl-L-  homoserine lactone | C_14_H_25_NO_3_ | 256 | 5.4 | TZWZKDULKILUPV-LBPRGKRZSA-N |
| 3-oxo-C12-HSL | *N*-(3-Oxododecanoyl)-L-  homoserine lactone | C_16_H_27_NO_4_ | 298 | 5.6 | PHSRRHGYXQCRPU-AWEZNQCLSA-N |
| C12-HSL | *N*-Dodecanoyl-L-  homoserine lactone | C_16_H_29_NO_3_ | 284 | 6.1 | WILLZMOKUUPJSL-AWEZNQCLSA-N |
| 3-oxo-C14-HSL | *N*-(3-Oxotetradecanoyl)-L-homoserine lactone | C_18_H_31_NO_4_ | 326 | 6.3 | YQFJJDSGBAAUPW-INIZCTEOSA-N |
| C14-HSL | *N*-Tetradecanoyl-L-  homoserine lactone | C_18_H_33_NO_3_ | 312 | 6.8 | ZQAYHOXXVBVXPZ-INIZCTEOSA-N |
